# The sustainability and the survivability of Kyoto’s traditional craft industry revealed from supplier-customer network

**DOI:** 10.1371/journal.pone.0240618

**Published:** 2020-11-09

**Authors:** Daisuke Sato, Yuichi Ikeda, Shuichi Kawai, Maxmilian Schich

**Affiliations:** 1 Graduate School of Advanced Integrated Studies in Human Survivability, Kyoto University, Kyoto, Japan; 2 The School of Arts, Technology, and Emerging Communication, The University of Texas at Dallas, Richardson, Texas, United States of America; Unviersity of Burgundy, FRANCE

## Abstract

Due to the changes in consumer demand and generational transformations, Kyoto’s traditional craft industry has suffered substantial revenue losses in recent years. This research aimed to characterize Kyoto’s traditional craft industry by analyzing the supplier-customer network involving individual firms within the Kyoto region. In the process, we clarify the community structure, key firms, network topological characteristics, bow-tie structure, robustness, the vulnerability of the supplier-customer network as crucial factors for sustainable growth. The community and bow-tie structure analysis became clear that the traditional craft industry continues to occupy an important position in Kyoto’s industrial network. Furthermore, we clarify the relationship between modern and traditional craft industries’ network characteristics and their relative profitability and productivity. It became evident that the traditional craft industry has a different network structure from the modern consumer games and electric machinery industries. The modern industries have the strongly coupled component, and the attendant firms there create high value-added and play a significant role in driving the entire industry, while more traditional craft industries, such as the Nishijin silk fabrics and Kyoto doll industries, do not have this strongly coupled component. Moreover, the traditional crafts industry does not have a central firm or a dense network for integrating information, which is presumed to be a factor in the decline of the traditional craft industry.

## Introduction

In a highly developed industrialized society, mass production is at the center of economic activity. This mass production arguably stands in stark contrast to production methods such as the “flexible division of labor” system [1] that characterizes the collaboration among firms and the manufacturing system of traditional craft industries. As early as 1980, such a traditional flexible division of labor attracted a certain amount of attention as a substitute model for the American-style mass production system in regional economies, such as those in and around Kyoto. However, with the improvement in the functions of the software and the development of Internet-based communication, the global supply chain has developed, and an international horizontal division of labor has been established over the last few decades [2]. Despite this, the flexible and regional-based production methods have recently begun to attract renewed interest as a developmental concept of redistributed manufacturing [3, 4]. Modern global market manufacturing tends to be characterized by centralized production and a complex global supply chain, has resulted in homogenized materials, cost volatility, and some uncertainty regarding energy and transportation costs [5–7]. Meanwhile, localized, flexible, and community-centered traditional craft manufacturing methods are expected to democratize the production and the markets and as well as simplify the supply chains [8].

Kyoto, the region of interest for this research, has a long history of crafts and is highly representative of Japanese culture as Japan’s long-time capital. The traditional crafts industry in Kyoto produces so-called “Kyoto products,” which are characterized by sophisticated sensibilities grounded in over a thousand years of history and a high-quality build produced through an extreme subdivision of labor system [9–11], where each manufacturing process is based on advanced craftsmanship that has been handed down from generation to generation. However, while the industry remains deeply rooted in the lives of the Japanese, the changes in consumer demand and the generational transformations have resulted in a substantial decrease in revenue for the industry [12–15]. It would appear that the formerly effective flexible division of labor, as well as the production equipment, know-how, and conventional procedures of a skilled labor force, have become increasingly unable to cope with the market fluctuations and technological changes brought about by economic globalization. Thus, it is crucial to clarify the operational forms of traditional craft industries and identify the attendant issues.

Here, we address this by establishing a method for clarifying the supplier-customer network structure among the individual firms within the current traditional craft industry in the Kyoto region. To this end, we regard the production activity in terms of complex networks consisting of firms (as nodes) and transaction relationships (as links). Recently, numerous studies have suggested that analyzing farm-level transaction relationships as a supplier-customer network could prove useful for understanding specific economic issues [16–18]. By taking Kyoto’s traditional crafts industry as a supplier-customer network formed by interfirm transaction relations, we can elucidate its characteristics in relation to other industries.

Our main goal is to understand the underlying structures and dynamics to nurture sustainability and ensure the traditional craft industry’s survival within the existing broader and ever-changing market environment. To present a detailed discussion, we examine the traditional craft industry’s problems from the viewpoint of sustainability and survivability of Kyoto’s traditional craft industry using actual data. We assume that a firm’s sustainability is the capability to operate the firm in a healthy state under normal economic conditions, measured by the productivity and profitability of firms. We also assume that the survivability of a firm is the capability to keep the firm functioning under exposure to critical situations, measured by firms’ robustness. Our research questions are 1) to what extent can the supplier-customer network’s characteristics explain productivity, profitability, robustness? 2) what is the difference between traditional craft industries and other industries? More specifically, the aim is to explain the profitability, productivity, and robustness of the industry in terms of the bowtie structure components and various centrality indicators for the traditional craft industry. As a result of our research, we expect to make a valuable contribution toward raising production efficiency while preserving the desired quality in terms of the traditional procedures and products.

The paper is organized as follows. In the next sections, we explain the dataset and the method of network analysis used in this paper. Following this, the results section, the main part of the paper, outlines the main characteristics and issues of the supplier-customer network of Kyoto’s traditional craft industry before the last section reviews the results and provides a conclusion.

## Materials and methods

### Data

The data analyzed in this paper is a subset of 5,943,072 supply chain transaction relationships among 1,668,567 individual Japanese firms (including the firm name, location, industry, number of employees, etc.), as investigated by Tokyo Shoko Research, Ltd (TSR) in 2016. From this data, we constructed a supplier-customer network, where each individual firm is a node, and specific links connect pairs of firms with at least one supplier-customer relationship, such that we could construct a directed graph. Due to the dataset limitation, the links have no weights. Focusing on firms in Kyoto and those that have business relationships with firms in the region, our analysis involves 79,678 nodes and 153,684 links.

### Data analysis procedure

First, we conduct a community analysis to identify representative industrial sectors in Kyoto. The identified industrial sectors are three traditional craft industries and three modern industries.

Secondly, in terms of the characterization of the industries identified as communities in the supplier-customer network in Kyoto, we estimate the centrality indices and the site of the Bow-tie structure. We focus on the three centrality indices here. Firms with a high degree centrality are considered advantageous for capturing the consumers’ needs and immediately respond to them in a rapidly changing market. The clustering coefficient is considered as a measure of exchanging information. A higher clustering coefficient means more efficient information exchange. Firms with a high betweenness centrality act as connectors to connect network groups with different economic roles. Strongly coupled component (SCC) in the Bow-tie structure is especially important. Because it is considered that the SCC works a feedback loop for the entire industry. Firms in the SCC create high value-added and play a major role in driving the industry.

Thirdly, we estimate the productivity and profitability for each firm as surrogate variables of the firms’ sustainability. Here, profitability and productivity are the ratio of profit to equity (Return on Equity) and the profit per employee, respectively. We note that more appropriate variables are value-added per employee as productivity. However, the value-added was not available in the current dataset used in this study. We also estimate the robustness for each firm as a surrogate variable of the survivability of the firm. The robustness is measured as the removal rate at which a node is removed from the maximum connected component by removing the firm’s links.

Finally, we develop an econometric model to explain the profitability, the productivity, and the robustness of firms in terms of sites of the bowtie structure and three centrality indicators for the traditional craft industry. The following questions 1) to what extent can productivity, profitability, robustness be explained by supplier-customer network characteristics? 2) what is the difference between traditional craft industries and other industries? will be answered based on the results of econometric analysis.

### Detection of community structure

Community analysis divides the network into closely related groups, and we used this method to distinguish the traditional craft industry network from the supplier-customer network in Kyoto. The most popular method for community detection is the modularity maximization approach [19]. However, as is well known, this method is not suitable for dividing large networks due to the significant resolution limitation issue. More importantly, we intend to identify communities of specific traditional craft industries. In general, each community consists of many subcommunities, and these subcommunities correspond to different industry sectors. The specific traditional craft industry might be identified as a subcommunity in a community of the supplier-customer network. In modularity maximization, it is necessary to analyze each community recursively to obtain the hierarchical community structure. However, in that case, the number of nodes in each community is different, so the modularity, which represents the goodness of sub-community division, cannot be compared between communities. The advantage of the map equation method is that such a hierarchical structure can be obtained naturally. Therefore, we used the map equation method [20, 21] for our analysis, which is one of the more accurate methods among those available [22]. A set of nodes with a high probability of staying when a random walker randomly walks on a network was regarded as a community when we applied the method to a given network. The map equation method involves encoding based on information theory, which ensures a reduction of the information’s overall breadth without losing the original information. When encoding, attention must be paid to the information frequency, with short codes assigned to the information with a high frequency of appearance and long codes assigned to that with a low frequency of appearance. A random walker is also highly likely to stay in a node set with a high link density and is also highly likely to move to another node set from one with a low density. In a well-divided community, the occurrence probability of a code indicating movement between communities is considered low. However, if some movement between communities is unlikely to occur, regarding the network as one community presents the best division. Therefore, in the map equation method, the average code length *L* in the community division *M* that divides the network into *C* is defined by

$$\begin{array}{c} L\left(M\right)=q_{⤼}H\left(Q\right)+\sum_{i=1}^{c}p_{⥁}^{i}H\left(P^{i}\right), \end{array}$$ (1)

where, *q*_⤼_ is the probability that a random walker moves to another community, *H*(*Q*) is the average description length of the community index codewords given by the Shannon entropy, $p_{⥁}^{i}$ is the probability that a random walker in community *i* will stay in the same community in the next step, *H*(*P*^*i*^) is the entropy of the codewords in the module codebook *i*, and *c* is the number of communities.

### Topological characteristics and centrality indices

We explain topological characteristics and centrality briefly to analyze the structure and the dynamics of each industry’s supplier-customer network.

The average shortest path length is given by the average number of the shortest paths for all possible pairs of nodes in the network. Given that the larger the average shortest path length is, the more firms are involved in manufacturing. The average shortest path length *R* is defined by $R=\sum_{i=1}^{N}\sum_{j=1(i\neq j)}^{N}d_{ij}/N(N-1)$. Here *d*_*ij*_ is the average shortest path length between nodes *i* and *j*, and *N* is the number of nodes in the network.

The clustering coefficient is used to measure how connected a node’s neighbors are to one another [23]. The clustering coefficient of a certain node *i* defined by *c*_*i*_ = *L*_*i*_/*k*_*i*_(*k*_*i*_ − 1). Here *k*_*i*_(*k*_*i*_ − 1) is total number of possible connections between the neighbors of node *i*, and *L*_*i*_ is the actual number of links among the neighbors of node *i*. The clustering coefficient *C* of an entire network is the average of the clustering coefficient of all nodes *i*, defined by $C=\sum_{i=1}^{N}c_{i}/N$.

Assortativity indicates the similarity among the connections in the graph concerning the node degree. If a node is connected to nodes with degree values similar to its own, the network is assortative, and its assortativity is close to 1, while if the opposite is the case, the network is disassortative, and its assortativity is close to -1. It is well known that biological networks tend to be disassortative, while social networks tend to be assortative. In the case of an assortative network, the damage to the network due to the removal of the hub node will be small, but in the case of a disassortative network, removing the hub node may cause significant damage to the network [24].

The density is calculated according to the proportion of the actual number of links in a graph within the maximum possible number of links and indicates how closely the entire network is connected. The density *D* of a network is defined by *D* = *M*/*N*(*N* − 1). Here *M* is the actual number of links in a graph.

Degree centrality is a measure according to the number of links on each node [25]. Firms with a high degree centrality are likely to play a role in aggregating information in the supplier-customer network because they hold numerous business relationships. To compare the degree centrality of different sized networks, normalization must be carried out by dividing the total degree of nodes by the maximum possible number of adjacent connections 2(*N* − 1). The degree centrality of a node *i* is defined by *x*_*i*_ = *k*_*i*_/2(*N* − 1). Here *k*_*i*_ is the number of degrees of node *i*.

Betweenness centrality is a measure according to the number of shortest paths passing through a node [26]. Firms with a high betweenness centrality act as connectors to connect network groups, and, in a supplier-customer network, they play the role of connecting firms with different economic roles. The betweenness centrality of a node *i* is defined by $x_{i}=1/N(N-1)\cdot \sum_{j=1(j\neq i)}^{N}\sum_{k=1(k\neq i)}^{N}n_{jk}\left(i\right)/n_{jk}$. Here *n*_*jk*_ is the number of shortest paths between nodes *j* and *k*, and *n*_*jk*_(*i*) is the number of paths that pass through node *i* among the shortest paths between nodes *j* and *k*. This was normalized by dividing the maximum number of pairs of nodes, excluding the node itself.

### Bow-tie structure

Bow-tie decomposition is widely used to understand the flow structure of various complex networks, such as hyperlink networks on the web [27] and metabolic networks [28]. Here, macroscopic flow structures of the graph were obtained to decompose the graph into components according to its nodes’ connectivity, i.e., each node was assigned to a given component according to its reachable nodes set. To clarify the bow-tie structure from the directed network *G*, we first referred to the undirected graph obtained by *G* without the direction of its links. Assuming this graph is G*, all nodes in *G* are classified as one of the following components:

- SCC: nodes in the largest strongly connected component of *G*.
- IN: nodes reachable to SCC, but not from SCC.
- OUT: nodes reachable from SCC, but not to SCC.
- TENDRIL: nodes not in the previous categories, reachable to OUT (OUT TENDRIL), or reachable to IN (IN TENDRIL), but not both.
- TUBE: nodes reachable from IN and to OUT, but not SCC.
- OTHER: nodes not connected to G*

Each component makes different contributions to the network in terms of economic activity, while the SCC plays a central role here. All industries in the specific economy are closely connected, and the growth of the core industries may have greater significance for the economy’s growth compared with that of the peripheral industries [29].

### Robustness

To measure network robustness, we applied the percolation theory to the supplier-customer network concept. Percolation theory informs us how many nodes or links must be removed for breaking down the network into isolated elements and is used to describe the stability of a system in terms of disturbances [24]. In the case of a supplier-customer network, the robustness corresponds with measuring the system’s stability in terms of economic crisis. In this study, we simulated two types of attacks: random failure and target attack. In the random failure simulation, we randomly selected and removed certain sets of nodes or links from the network, while for the target attack simulation, we removed specific sets of nodes or links in decreasing order of centrality. Here, two types of centrality, degree centrality, and betweenness centrality, were simulated. We could then measure the change in the size of the largest connected component to determine the robustness.

## Results

### Kyoto’s traditional craft industries identified as cubcommunities

**Fig 1:**
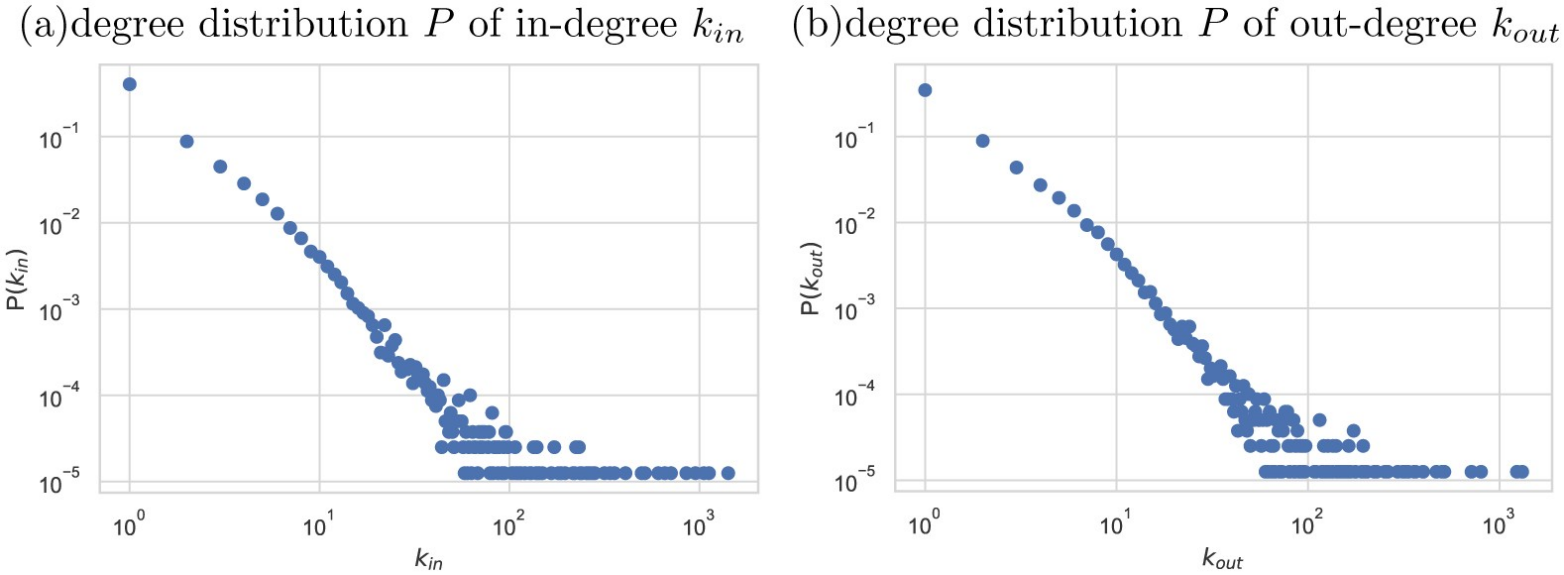
In- and out- degree distribution of Kyoto’s supplier-customer network. Logarithmic binning of the horizontal and vertical axes is used in (a) and (b). The power law tail of the degree distribution can be observed here.

First, we calculated the degree distribution to better understand the trends of the Kyoto supplier-customer network. As shown in Fig 1, it was confirmed that both in and out-degree distribution is a scale-free network where the tail of the distributions is characterized by a power law of the form $P\left(k_{in/out}\right)∼k^{-\gamma_{in/out}}$ with *γ*_*in*_ = 2.34 and *γ*_*out*_ = 2.38 respectively. The power-law tail of the degree distribution is also presented in the past research of the empirical supply chain network [30, 31].

**Fig 2:**
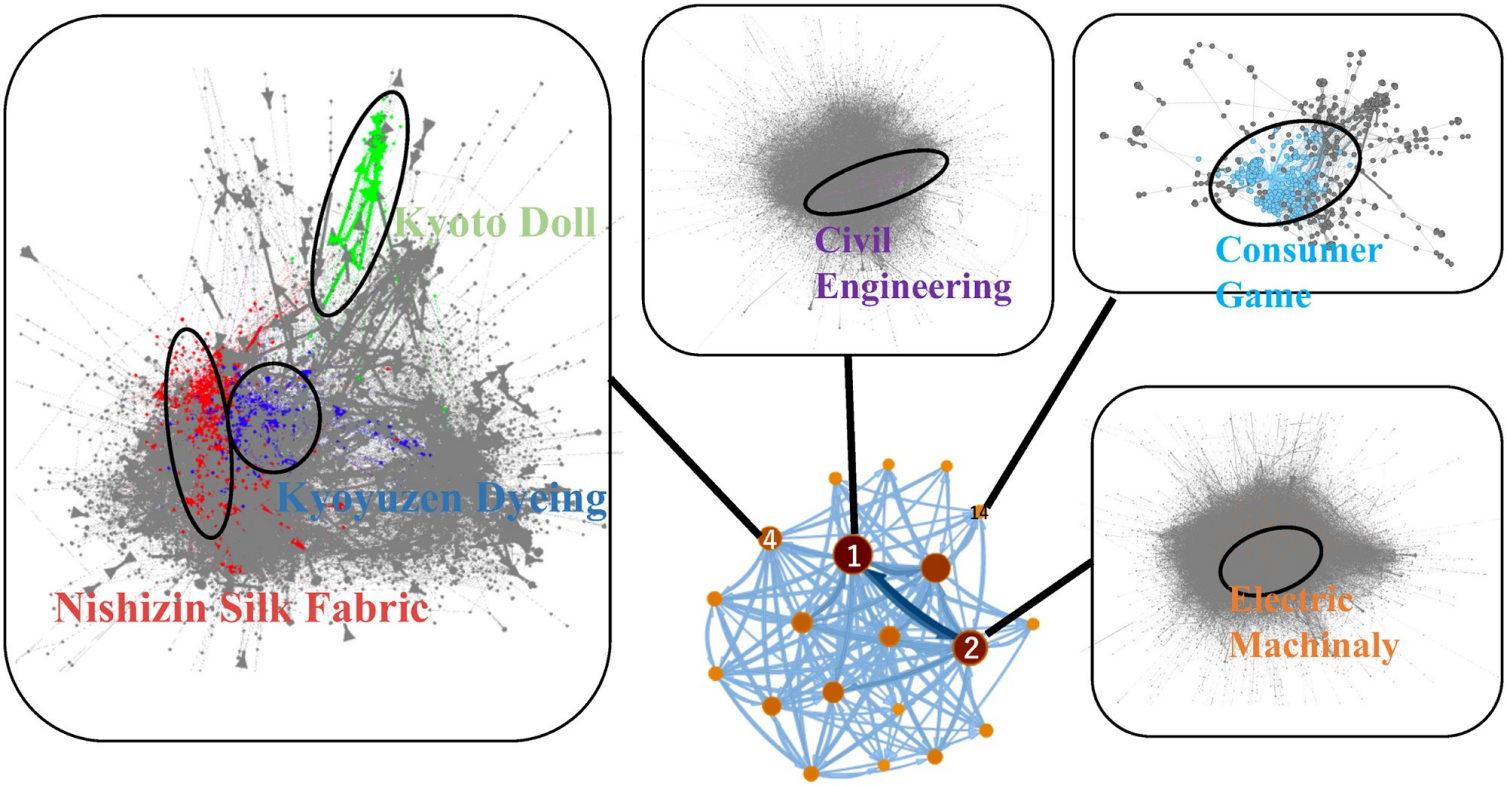
Community structure of Kyoto’s supplier-customer network. The network below the center represents Kyoto’s supplier-customer network. Here, the nodes represent a first-layer community. Of the 1,212 communities, only the largest 20 are described. The highlighted community is the community we analyzed. The nodes of the highlighted community network are firms.

Following this, we identified the supplier-customer network communities in Kyoto’s traditional craft industry using a map equation and subsequently, the 79,678 firms related to Kyoto into 1,212 communities. Each community has a hierarchical structure, which consists of subcommunities in a lower hierarchical layer. Here subcommunity a-b means that community a includes subcommunity b. Table 1 shows the detailed results of the 20th largest communities in 1st level communities. Two communities are by far the largest. The community of index 2 is the largest, and the community’s primary sector is the manufacturing sector. In the second-largest community, mainly the construction sector was observed. From the hierarchical structure, we chose three communities as representatives of Kyoto’s traditional craft industry: the Nishijin silk fabrics industry, the Kyoyuzen dyeing industry, and the Kyoto doll industry. The traditional craft industry communities were established by identifying the communities to which the firms of each of the traditional craft association list belong [32–39]. For instance, as shown in S1 to S6 Tables of the S1 Appendix, subcommunity 4-1 consists of 174 firms, and among these firms, 133 firms (76%) are included in the traditional craft association list of the Nishijin silk fabrics industry. We assume that 41 firms not included in the association list are relatively unknown firms but are deeply involved in the industry. Thus, subcommunity 4-1 is identified as the Nishijin silk fabric industry. Similarly, subcommunity 4-6 consists of 94 firms, and among these firms, 53 firms (56%) are included in the traditional craft association list of the Kyoyuzen dyeing industry. We assume that 41 firms not included in the association list are relatively unknown firms but are deeply involved in the industry. Thus, subcommunity 4-6 is identified as the Kyoyuzen dyeing industry. Similarly, subcommunity 4-25 consists of 71 firms, and among these firms, 15 firms (21%) are included in the traditional craft association list of the Kyoto doll industry. We assume that 56 firms not included in the association list are relatively unknown firms but are deeply involved in the industry. Thus, subcommunity 4-25 is identified as the Kyoto doll industry. It can be seen that the traditional craft industry exists within the same first-level community. Fig 2 shows the embedding of all three industries within Kyoto’s supplier-customer network as a whole. To compare the traditional craft communities, we also analyzed the supplier-customer network of Kyoto’s leading industries, including the consumer games industry (subcommunity 14-1), the electric machinery industry (subcommunity 2-18), and the civil engineering industry (subcommunity 1-115).

**Table 1:** Overview of the 1st level communities.

Index	Size	Sector
1	16911	Construction(0.57), Wholesale and retail trade(0.16)
2	17892	Manufacturing(0.44), Wholesale and retail trade(0.3)
3	9664	Wholesale and retail trade(0.44), Manufacturing(0.27)
4	5911	Wholesale and retail trade(0.6), Manufacturing(0.25)
5	2866	Medical, health care and welfare(0.32), Wholesale and retail trade(0.3), Manufacturing(0.18)
6	3630	Wholesale and retail trade(0.37), Transport and postal activities(0.21), Manufacturing(0.12), Services, N.E.C.(0.1)
7	4262	Manufacturing(0.39), Wholesale and retail trade(0.27)
8	5089	Wholesale and retail trade(0.36), Transport and postal activities(0.23), Manufacturing(0.13)
9	837	Wholesale and retail trade(0.42), Manufacturing(0.27), Construction(0.11)
10	1220	Wholesale and retail trade(0.41), Manufacturing(0.4)
11	2003	Construction(0.44), Wholesale and retail trade(0.32), Manufacturing(0.15)
12	677	Manufacturing (0.44), Wholesale and retail trade (0.35)
13	1189	Wholesale and retail trade(0.72), Manufacturing(0.11)
14	547	Wholesale and retail trade(0.35), Information and communications(0.3)
15	437	Living-related and personal services and amusement services (0.27), Wholesale and retail trade(0.24), Manufacturing (0.15), Construction(0.1)
16	285	Construction (0.28), Real estate and goods rental and leasing (0.2), Services, N.E.C.(0.14), Wholesale and retail trade(0.11)
17	340	Construction (0.38), Wholesale and retail trade (0.2), Manufacturing (0.13)
18	522	Wholesale and retail trade(0.46), Living-related and personal services and amusement services(0.26), Manufacturing (0.1)
19	413	Wholesale and retail trade(0.52), Living-related and personal services and amusement services(0.28), Manufacturing (0.12)
20	124	Wholesale and retail trade (0.26), Manufacturing (0.14), Information and communications(0.1), Scientific research, professional and technical services(0.1), Services, N.E.C.(0.1)

The overview of the 20th largest communities in the 1st level. “Size” is the number of firms that the community has. The percentage of nodes classified with a particular industry sector is shown in parentheses. This classification is based on Japan Standard Industrial Classification, November 2007, Revision 12. Those with less than 0.1 are not listed.

### Key firms in the selected industries

**Fig 3:**
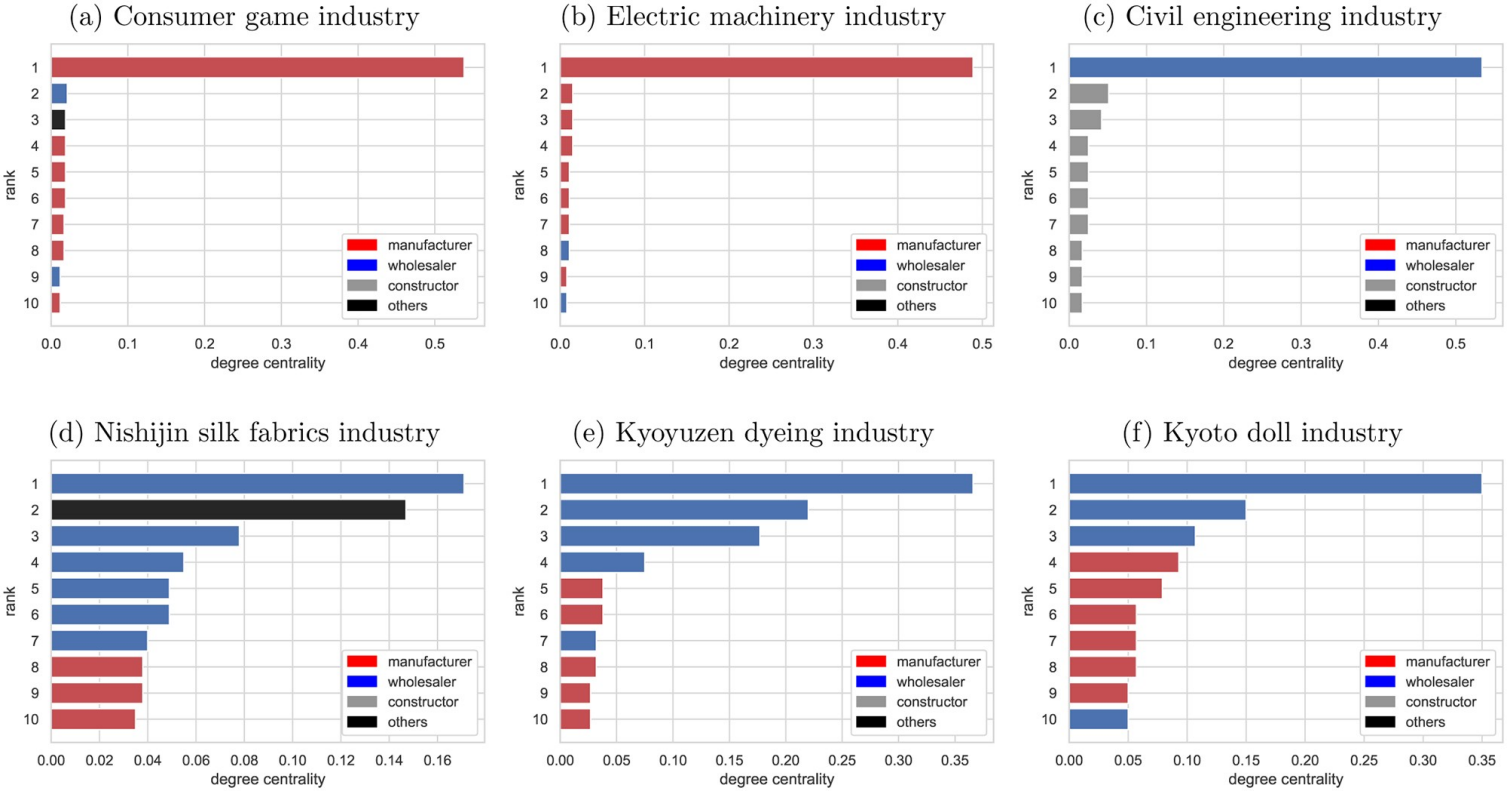
Degree centrality of Kyoto’s supplier-customer network. The color of the bar distinguishes the business type, with red, blue, gray, and black designating manufacturer, wholesaler, constructor, and others, respectively.

**Fig 4:**
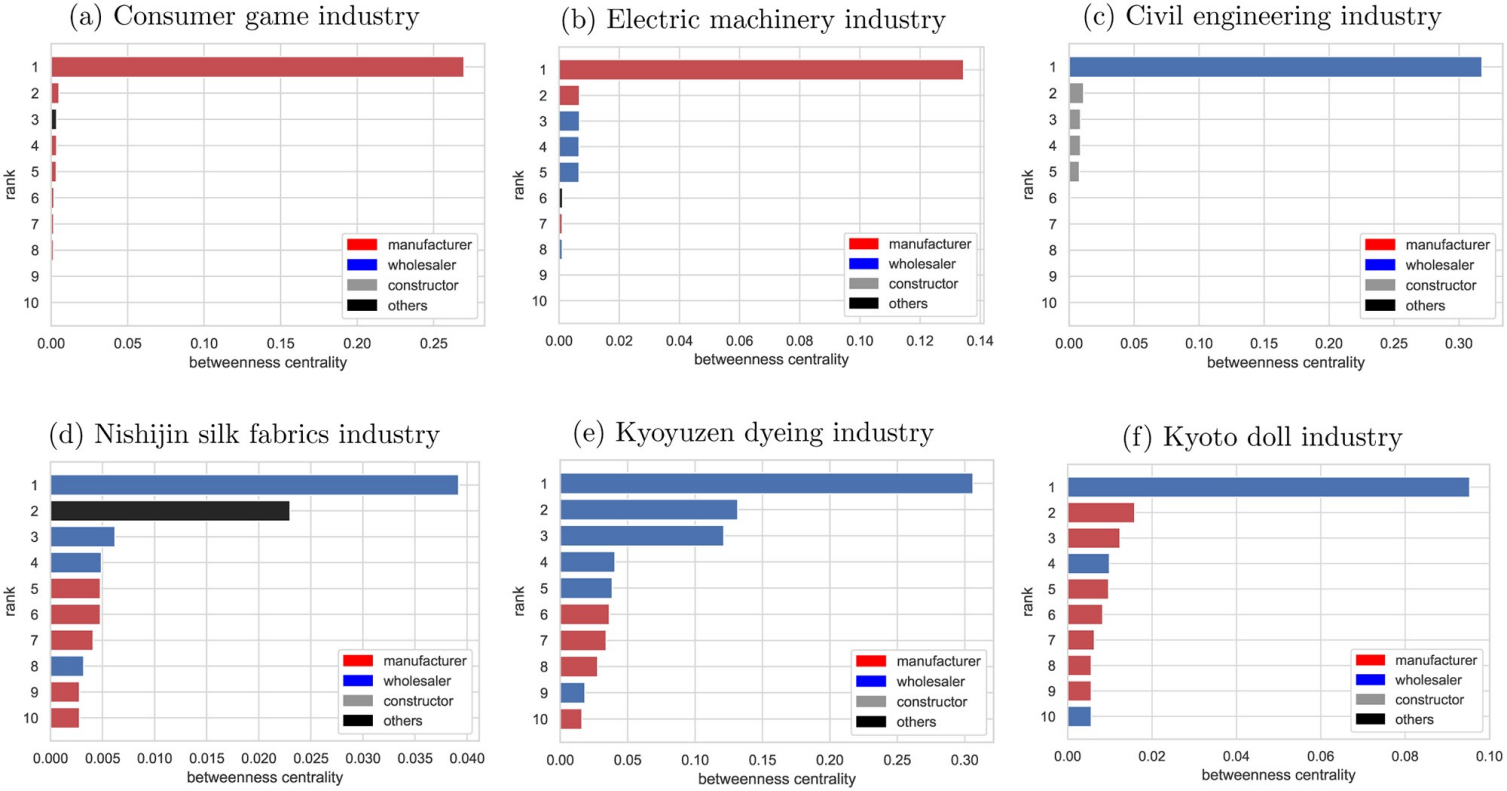
Betweenness centrality of Kyoto’s supplier-customer network. The color of the bar distinguishes the business type as with Fig 3.

By identifying the nodes that play a central role in the network, we could clarify the key firms in each of the selected industries’ supplier-customer networks as detected as subcommunities. Figs 3 and 4 show the centrality and business type of the top 10 firms with high degree centrality and betweenness centrality. The results indicated that the consumer games, electric machinery, and civil engineering industries are the networks where the most central players play a significant role. These industries are representative of modern industries in Kyoto. The consumer games and electric machinery industries include some of the world’s most well-known firms. The civil engineering industry is one of the largest subcommunities in Kyoto. The most central firm of these subcommunities has around 0.5 degree centrality, indicating more than half of firms in the subcommunity have a supplier or customer relationship with the firm. However, the business type of central players is different. In the consumer games and electrical machinery industries, the most central player is the manufacturer, while in the civil engineering industry, the wholesaler that sells the equipment and materials is the central player. It is also clear that the consumer games and electric machinery industries have a centralized production system. Compared to such industries, the traditional craft industries have no outstanding central firms and represent a decentralized supply chain. Characteristically, the most central firm in all the traditional craft industries is the wholesaler in contrast to consumer games and electric machinery industries. The fact that the wholesalers have many business relationships indicates that the roles of wholesale and manufacturing are divided in Kyoto’s traditional craft industry. As for betweenness centrality, there are only firms with less than 0.1 betweenness centrality in Nishijin silk fabrics and Kyoto doll industries, suggesting these subcommunities have no firms responsible for aggregating information and managing the entire supply chain.

### Topological characteristics of the selected industries

Table 2 presents the topological characteristics of the selected industries. The assortativity is negative in all the subcommunities, i.e., the nodes with many links tend to be connected to nodes with fewer links. The supplier-customer network is considered a network with relatively low robustness in removing any hub nodes from the network. Concerning the average shortest path length, all subcommunities we analyzed have a value between 2.0 and 3.0, and there seems to be little difference between subcommunities. However, the feature of the network of each subcommunity varies; we had to be cautious when comparing the topological characteristics. For this reason, the network quantities were calculated for networks with degree-preserving randomization of each industry; these are shown in parentheses. Compared with the networks with degree-preserving randomization, modern industries’ subcommunities have almost the same average shortest path length. In contrast, those of the Nishijin silk fabrics and Kyoto doll industries are short, indicating that the Nishijin silk fabrics and Kyoto doll industries have low-cost supply chain structures. Besides, the traditional craft industries have a reasonably low clustering coefficient compared to the networks with degree-preserving randomization, suggesting these industries do not have a structure in which information is efficiently shared, but are divided.

**Table 2:** Comparison of traditional craft industries with modern industries.

	Kyoto traditional craft industries			Modern industries		
	Nishijin silk fabrics	Kyoyuzen dyeing	Kyoto doll	Consumer games	Electric machinery	Civil engineering
Number of nodes	174	94	71	212	132	60
Number of links	416	186	161	325	150	76
Density	0.0137	0.0208	0.0315	0.0072	0.00854	0.0207
Average path length	2.38 (4.19)	2.99(2.66)	2.24(3.05)	2.05(2.07)	2.15(2.04)	2.04(2.054)
Clustering coefficient	0.00344 (0.086)	0.0301 (0.254)	0.0518 (0.216)	0.101 (0.191)	0.0344 (0.0565)	0.0394 (0.12)
Average betweenness	0.00083 (0.00706)	0.00934 (0.00709)	0.0027 (0.01191)	0.00135 (0.00151)	0.00122 (0.00108)	0.00563 (0.00582)
Assoratativity	-0.350 (-0.209)	-0.595 (-0.463)	-0.454 (-0.409)	-0.564 (-0.564)	-0.681 (-0.677)	-0.741(-0.738)
Average profitability	7.8	25.6	32.5	14.1	10.5	56.1
Average productivity	35.5	36.3	27.6	93.2	65.9	69.6

The network quantities averaged for 1000 sampled networks with degree-preserving randomization are shown in parentheses. Note that the density is equal to the average degree centrality. The unit of productivity is million JPY.

### Profitability and productivity in bow-tie structure

**Fig 5:**
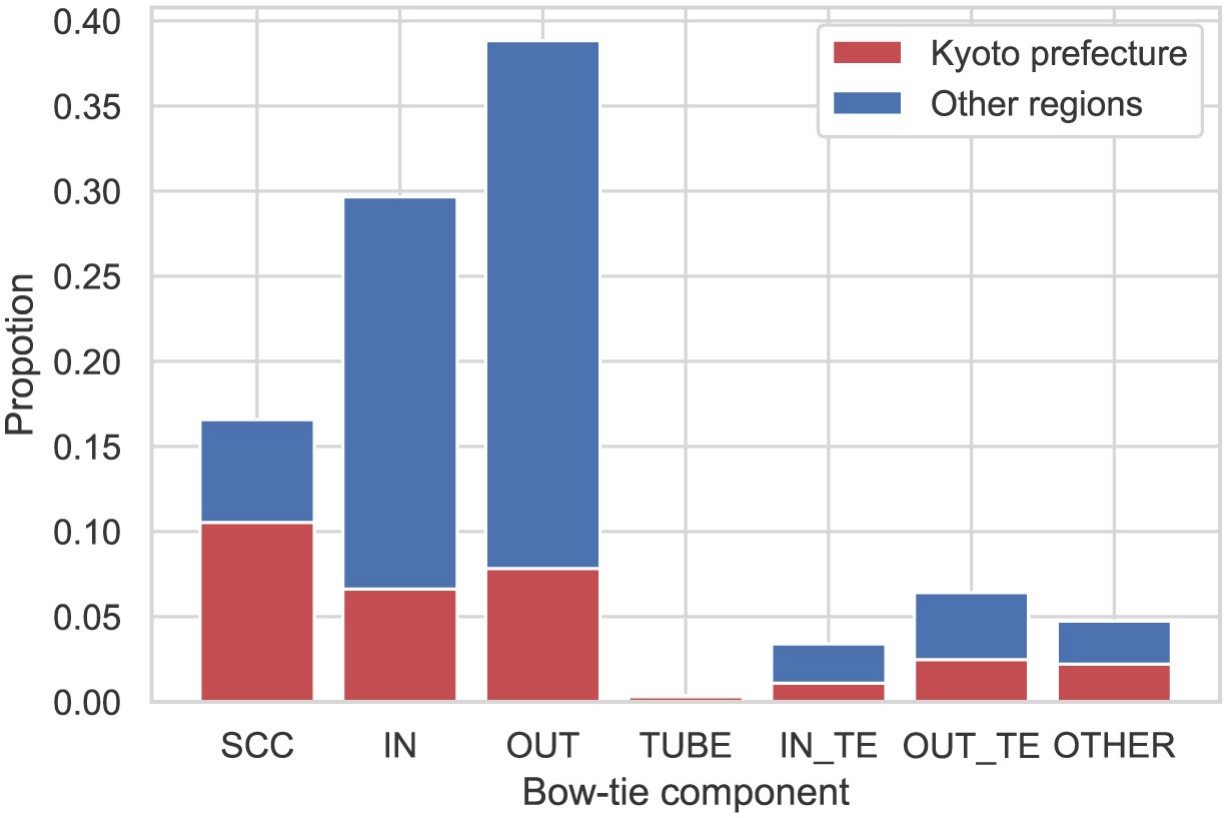
Distribution of firms in each bow-tie component of Kyoto’s supplier-customer network. “Proportion” refers to the ratio of the number of firms to the total number of firms in the largest weekly connected component in Kyoto’s supplier-customer network. The color of the bars denotes where the firms are located, with red designating Kyoto prefecture and blue the other regions.

Fig 5 presents the distribution of the firms in each bow-tie component when Kyoto supplier-customer network is decomposed into a bow-tie structure. The distribution is significantly different from the well-known distribution of firms in the bow-tie components of the hyperlink network on the web. The hyperlink network has 27.74% of nodes in the SCC category and 8.24% of nodes in the OTHER category, while IN, OUT, and TENDRIL have similar figures (21.29%, 21.29%, and 21.52%, respectively) [27]. Meanwhile, the largest component of Kyoto’s supplier-customer network is the OUT component (38.84%), followed by IN (29,67%), SCC (16,57%), OUT TENDRIL (6.41%), OTHER (4.74%), IN TENDRIL (3.39%), and TUBE (0.36%). These figures also differ from those related to the Japanese supplier-customer network analyzed in a previous paper [18], which indicated that half of the firms were in the SCC category. This difference is due to the extraction of Kyoto’s firms, where we not only used the firms in Kyoto but also those that have a supplier-customer relationship with these firms. As shown in Fig 5, the SCC category accounts for the largest number of firms in Kyoto, while IN and OUT account for many firms in the other regions. It is clear that Kyoto firms hold a significant number of transaction relationships with the OUT side and that they play a role in supplying many products to other prefectures.

**Fig 6:**
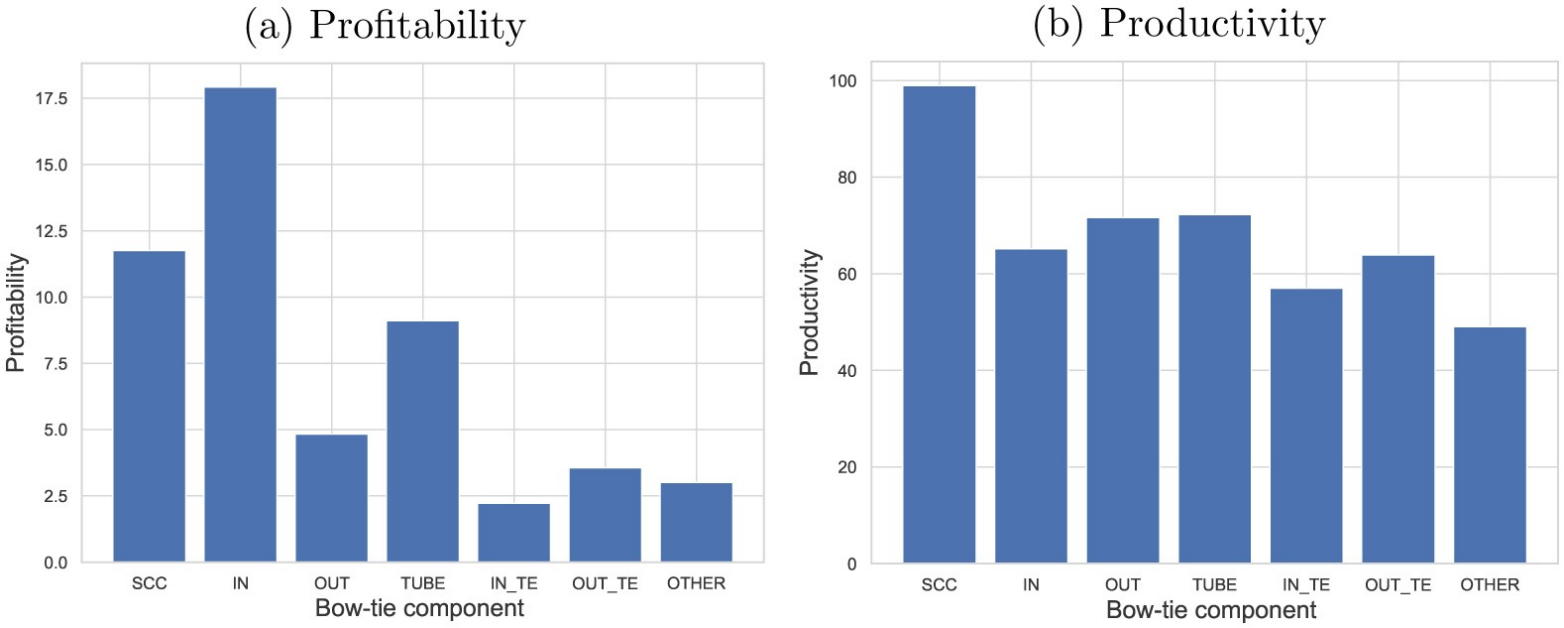
Profitability and productivity of the bow-tie components in Kyoto’s supplier-customer network. The unit of productivity is million JPY. In terms of productivity, SCC has the highest and IN the lowest value among SCC, IN, and OUT.

Fig 6 presents the profitability and productivity of the bow-tie components in Kyoto’s supplier-customer network. Profitability is the ratio of profit to equity (Return on Equity), while productivity is the profit per employee. We calculated the values using the data related to profit, sales, and the number of employees in the TSR dataset. In terms of profitability, there was a difference between the components, but the values for SCC were slightly lower, and that for IN was the highest among SCC, IN, and OUT. In contrast, in terms of productivity, SCC has the highest and IN the lowest value among SCC, IN, and OUT. In Kyoto’s supplier-customer network, the firms that distribute in SCC that forms the core of the network operate more productive than firms in the IN category that supply products to Kyoto.

**Fig 7:**
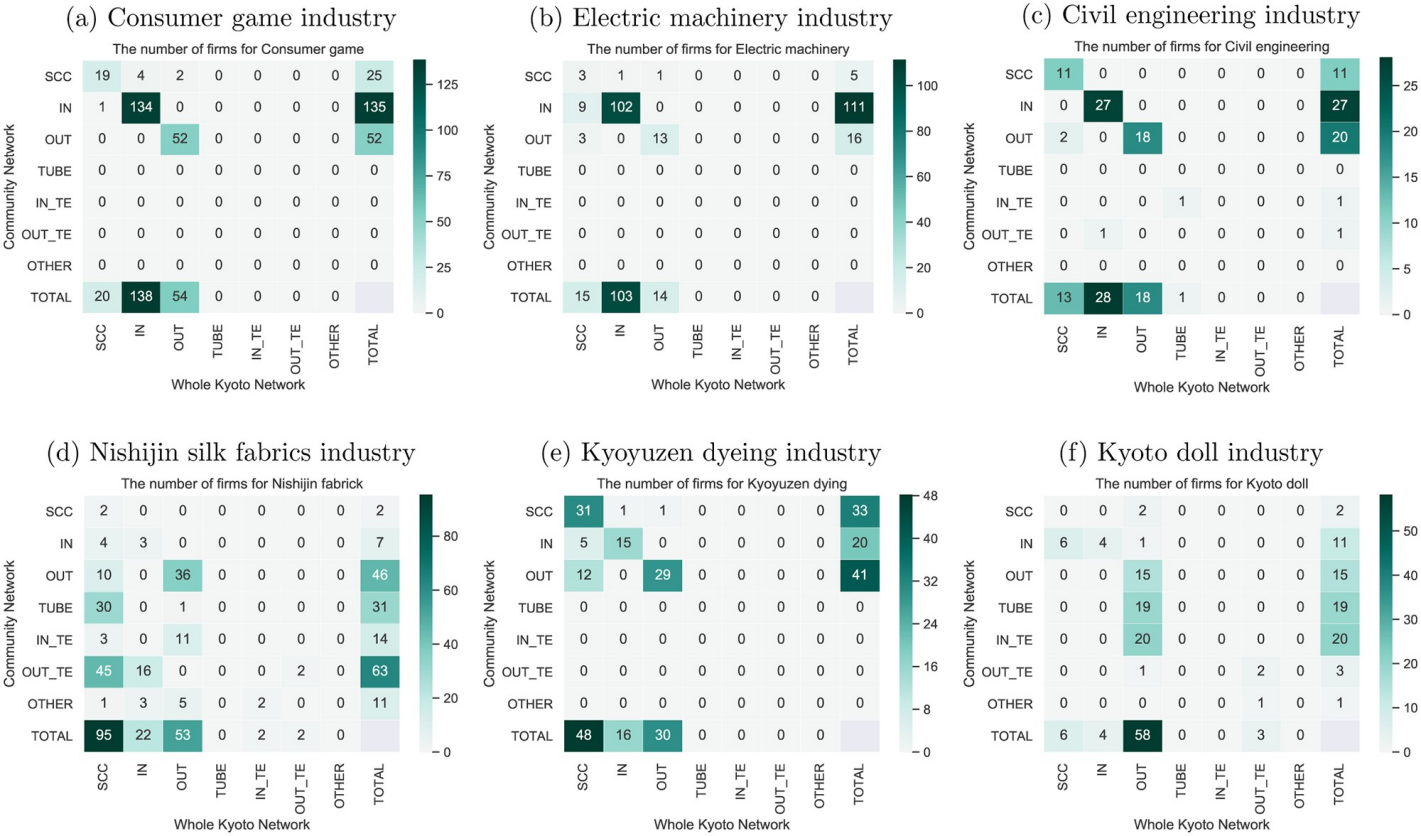
Distribution of firms in each bow-tie component of the selected subcommunities. The numbers represent the number of firms. The Nishijin silk fabrics and Kyoto doll industries have a relatively small number of firms in SCC.

Fig 7 presents the distribution of the firms in each bow-tie component of the selected industries. This analysis aimed to infer each selected industry’s role within Kyoto’s supplier-customer network and to reveal the flow structure of each community’s supplier-customer network. The horizontal axis is the bow-tie components of the selected industry in Kyoto’s supplier-customer network, and the vertical axis the bow-tie components in the selected industry. Here, bow-tie decomposition was applied again for each selected industry to obtain the selected industry’s bow-tie components. The results indicate that more nodes are distributed in the IN category for the consumer games, electric machinery, and civil engineering industries, the SCC for the Nishijin silk fabrics and Kyoyuzen dyeing industries, and the OUT for the Kyoto doll industry. The Nishijin silk fabrics and Kyoyuzen dyeing industries are located in the SCC of Kyoto’s supplier-customer network, indicating that these industries play an essential role in Kyoto’s economy relatively independent of other regions. This suggests that the decline of these industries could have a negative effect on Kyoto’s economy as a whole. Meanwhile, the bow-tie structure of each industry reveals that while almost all the nodes in consumer games, electric machinery, civil engineering, the Nishijin silk fabrics, and Kyoyuzen dyeing industries are located in the SCC, IN and OUT components, The Kyoto doll industry nodes have an extremely small SCC; that is, there is no feedback loop among the firms in this industry.

**Fig 8:**
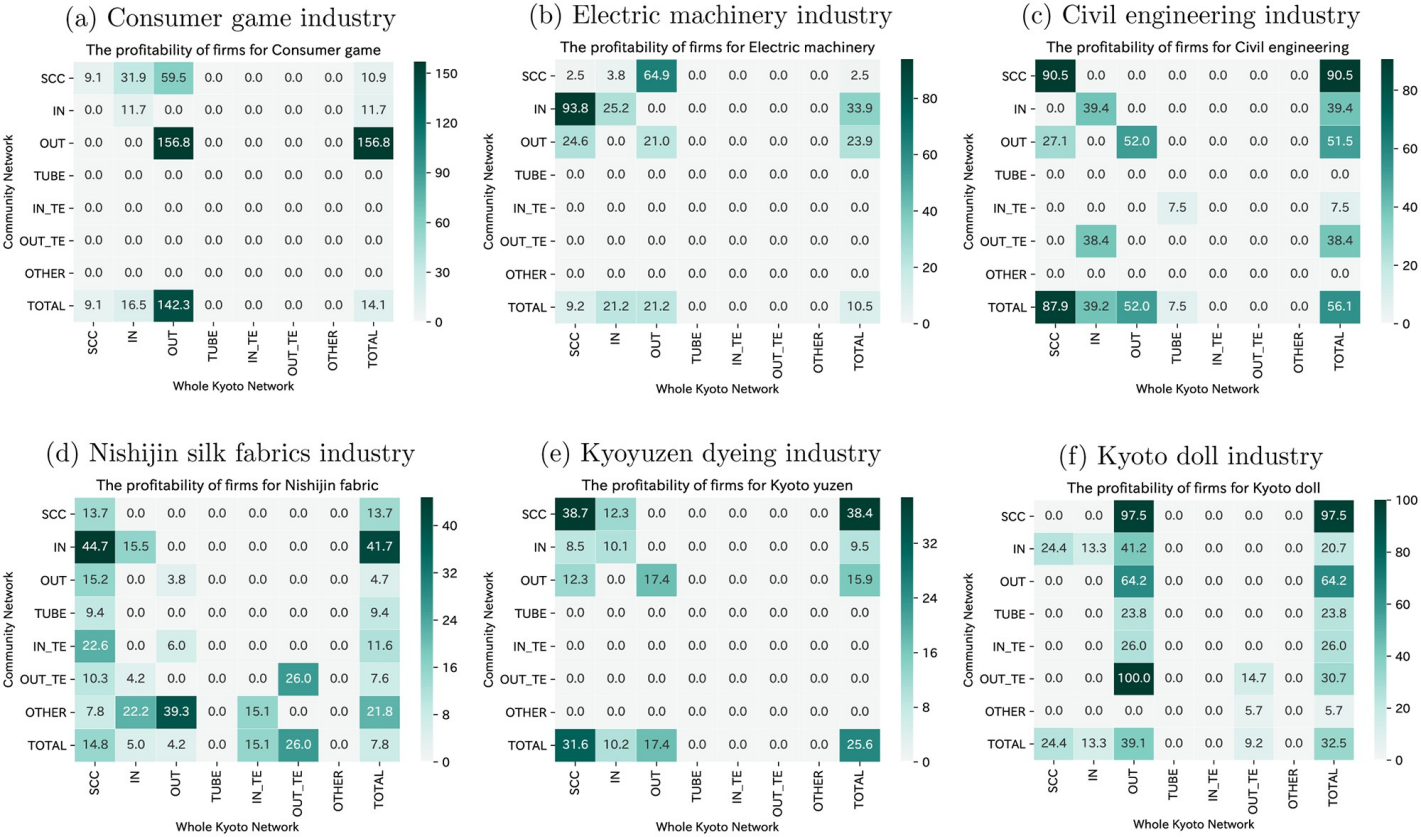
Profitability of each bow-tie component of the selected industries. The firms in SCC have high profitability within the consumer games and electric machinery industries.

**Fig 9:**
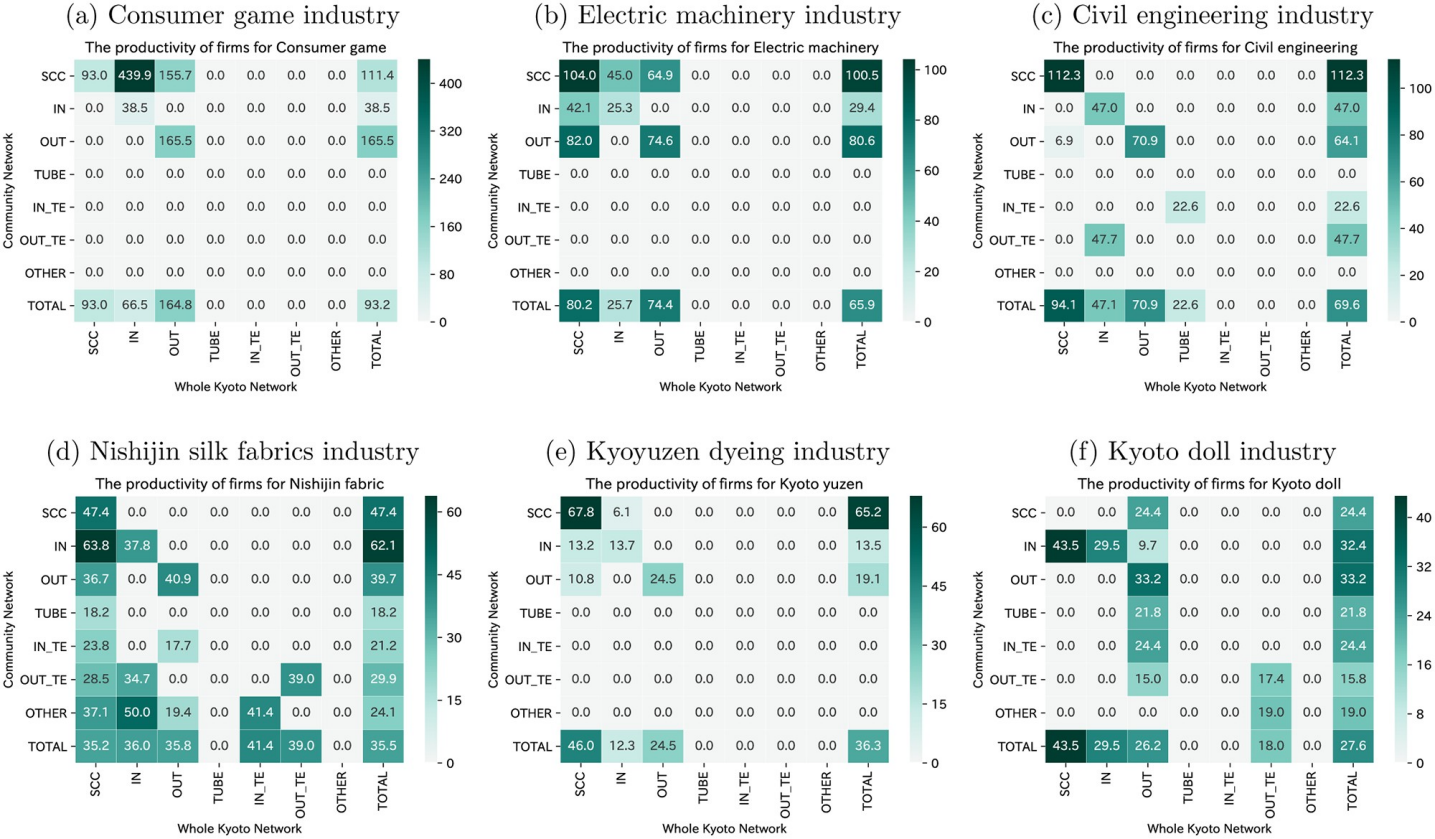
Productivity of each bow-tie component of the selected industries. The unit of productivity is million JPY. The firms in SCC have high productivity within the consumer games and electric machinery industries.

The profitability and productivity of each bow-tie component of the selected industries are shown in Figs 8 and 9, respectively. On examining each industry’s profitability and productivity as a whole, it is clear that the consumer games industry has the highest profitability and productivity, followed by the electric machinery industry. These industries present relatively efficient economic activities and are producing high value-added compared to other industries. These two industries have in common that the nodes in the SCC category have high profitability and productivity within the industry. The industry’s core firms create a great deal of value-added, which has a positive effect on the entire industry. However, it cannot be argued that the traditional craft industry has nodes that have high profitability and productivity in the SCC category, and it is clear that they may not be able to add value to the SCC of the economy. Besides, even though many firms of the Nishijin silk fabrics industry are located in the SCC category in Kyoto’s supplier-customer network, its total profitability, and productivity are low, which indicates that improving the Nishijin silk fabrics industry’s business situation is essential for Kyoto’s economy as a whole.

### Robustness of the selected industries

**Fig 10:**
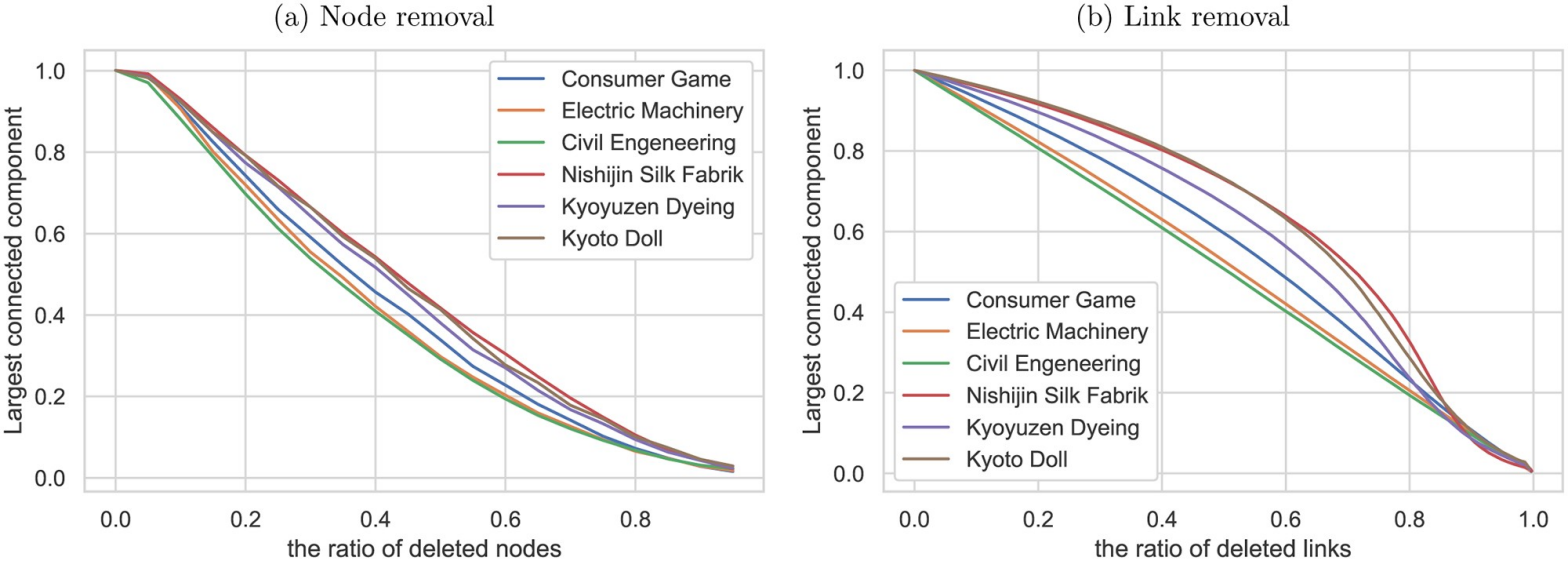
Random failure simulation. Response of the selected industries to random failure; the size of the largest connected component is plotted against (a) the percentage of nodes removed and (b) the percentage of nodes or links removed from each network.

The robustness of the network is an extremely important factor for the traditional craft industries, where the number of firms is declining rapidly. Fig 10 presents the results of the random failure simulation. Here, random failure is assumed to emulate bankruptcy in an economic crisis. The simulation shows the change in the largest connected component’s size when sets of nodes or links are randomly removed from the network. The size of the largest connected component is the average number of 1,000 trials of random failure simulation. The supplier-customer networks of the Nishijin silk fabrics and Kyoto doll industries are the most robust, followed by that of the Kyoyuzen dyeing industry. The traditional crafts industry’s supplier-customer network makes it possible to build a resilient supply chain to economic crises and bankruptcy.

**Fig 11:**
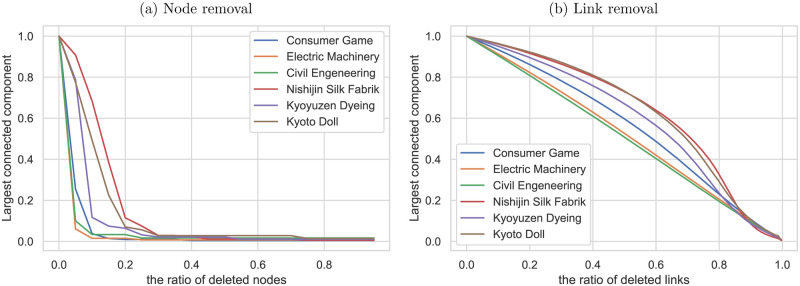
Target attack simulation (Degree centrality). Response of the selected industries to target attack; the size of the largest connected component is plotted against (a) the percentage of links removed and (b) the percentage of nodes or links removed in order of decreasing degree centrality from each network.

**Fig 12:**
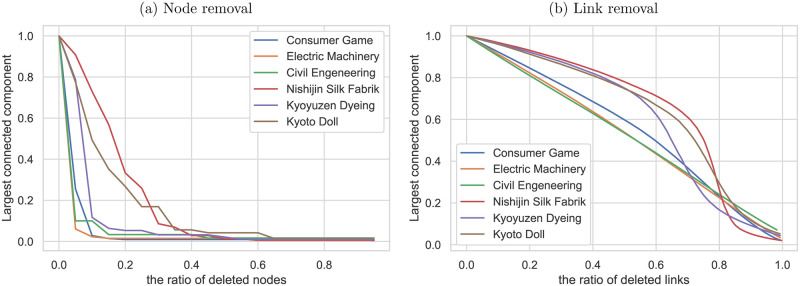
Target attack simulation (Betweenness centrality). Response of the selected industries to target attack; the size of the largest connected component is plotted against (a) the percentage of links removed and (b) the percentage of nodes or links removed in order of decreasing betweenness centrality from each network.

Figs 11 and 12 show the results of the target attack simulation. Here, the target attack is assumed to emulate the bankruptcy due to successor issues and corporate takeover involving subsequent corporate restructuring. The simulation shows the change in the size of the largest connected component when sets of nodes or links are removed to decrease degree centrality or betweenness centrality from the network. Compared to the random failure simulation, the largest component’s size decreased much faster in the target attack simulation. The analysis also demonstrates that the traditional craft industries’ supplier-customer networks are more robust than those of more modern industries. The decentralized supplier-customer network of the traditional craft industries may have contributed to the robustness of these industries.

## Discussion and implication

The division of labor, manufacturing equipment, know-how, and skilled workers of the traditional craft industry in Kyoto has not allowed the industry to respond to market structure changes or technological innovation. We designated Kyoto’s traditional crafts industry as a supplier-customer network formed by interfirm transaction relations to elucidate its characteristics and the reasons behind the industry’s decline from a network theory perspective.

The extensive dataset of interfirm supplier-customer relations related to Kyoto allowed us to analyze the traditional craft industry structure concerning those of other industries. Specifically, we focused on the community structure, the bow-tie structure, the robustness, and the supplier-customer network’s vulnerability as key factors for sustainable growth. From the community analysis and the bow-tie structure analysis, it became clear that the traditional craft industry continues to play an important role in Kyoto’s supplier-customer network.

We constructed econometric models that consider the various structural characteristics variables simultaneously to realize the inter-dependencies among the structural characteristics, robustness, profitability, and productivity. The robustness *R*_*lr*_, *D*_*lr*_, *B*_*lr*_ are average link removal rates when each node is out of the largest connected component for 1000 samples in random link removal, link removal proportional to the degree, link removal proportional to the betweenness centrality, respectively. The profit to equity (Return on Equity) and the profit per employee are used as surrogate variables of the profitability and productivity, respectively.

The regression analyses of the robustness *R*_*lr*_, *D*_*lr*_, *B*_*lr*_, the profitability, and the productivity was conducted using the explanatory variables: degree centrality, betweenness centrality, clustering coefficient, and part of bow-tie structure (IN, OUT, SCC). For the modern industry sectors, the results of the regression analyses of *R*_*lr*_, *D*_*lr*_, *B*_*lr*_, the profitability, and the productivity are shown in Tables 3–7, respectively. For the traditional craft industry sectors, the results of the regression analyses of *R*_*lr*_, *D*_*lr*_, *B*_*lr*_, the profitability, and the productivity are shown in Tables 8–12, respectively. The results of the regression analyses for each industry sectors: consumer game industry, electric machinery industry, civil engineering industry, Nishijin fabrics industry, Kyoyuzen dyeing industry, Kyoto doll industry, are shown in S7 to S36 Tables of the S1 Appendix.

**Table 3:** Regression of *R*_*lr*_ for the modern industries. Multiple *R*^2^: 0.4774, Adjusted *R*^2^: 0.4621, F-statistic: 31.26 on 9 and 308 DF, p-value: <2.2 × 10^−16^.

Variable	Estimate	Std. Error	t value	*Pr*(>|*t*|) 0 ‘***’ 0.001 ‘**’ 0.01 0.01 ‘*’ 0.05 ‘.’ 0.1
(Intercept)	4.937 × 10^−1^	9.085 × 10^−3^	54.340	<2 × 10^−16^ ***
Degree	1.460 × 10^−3^	3.875 × 10^−4^	3.769	0.000196 ***
Betweenness	9.370 × 10^−1^	2.345 × 10^−1^	3.995	8.09 × 10^−5^ ***
Clustering	1.348 × 10^−1^	2.466 × 10^−2^	5.466	9.54 × 10^−8^ ***
Profitability	−2.897 × 10^−5^	1.651 × 10^−5^	-1.755	0.080306 .
Productivity	7.082 × 10^−5^	3.813 × 10^−5^	1.857	0.064226 .
IN	−5.506 × 10^−3^	8.036 × 10^−3^	-0.685	0.493789
SCC	3.574 × 10^−2^	1.165 × 10^−2^	3.068	0.002347 **
SectorCivil	−5.986 × 10^−3^	9.884 × 10^−3^	-0.606	0.545215
SectorGame	−7.885 × 10^−3^	7.306 × 10^−3^	-1.079	0.281352

**Table 4:** Regression of *D*_*lr*_ for the modern industries. Multiple *R*^2^: 0.6003, Adjusted *R*^2^: 0.5886, F-statistic: 51.4 on 9 and 308 DF, p-value: <2.2 × 10^−16^.

Variable	Estimate	Std. Error	t value	*Pr*(>|*t*|) 0 ‘***’ 0.001 ‘**’ 0.01 0.01 ‘*’ 0.05 ‘.’ 0.1
(Intercept)	4.754 × 10^−1^	1.020 × 10^−2^	46.623	<2 × 10^−16^ ***
Degree	1.767 × 10^−3^	4.349 × 10^−4^	4.063	6.15 × 10^−5^ ***
Betweenness	7.842 × 10^−1^	2.632 × 10^−1^	2.979	0.003118 **
Clustering	4.416 × 10^−1^	2.768 × 10^−2^	15.955	<2 × 10^−16^ ***
Profitability	−3.279 × 10^−5^	1.853 × 10^−5^	-1.770	0.077758 .
Productivity	8.946 × 10^−5^	4.280 × 10^−5^	2.090	0.037410 *
IN	1.683 × 10^−3^	9.020 × 10^−3^	0.187	0.852139
SCC	2.520 × 10^−2^	1.308 × 10^−2^	1.927	0.054870 .
SectorCivil	−3.534 × 10^−2^	1.109 × 10^−2^	-3.186	0.001592 **
SectorGame	−2.983 × 10^−2^	8.200 × 10^−3^	-3.638	0.000323 ***

**Table 5:** Regression of *B*_*lr*_ for the modern industries. Multiple *R*^2^: 0.6447, Adjusted *R*^2^: 0.6343, F-statistic: 62.09 on 9 and 308 DF, p-value: <2.2 × 10^−16^.

Variable	Estimate	Std. Error	t value	*Pr*(>|*t*|) 0 ‘***’ 0.001 ‘**’ 0.01 0.01 ‘*’ 0.05 ‘.’ 0.1
(Intercept)	4.807 × 10^−1^	8.808 × 10^−3^	54.580	<2 × 10^−16^ ***
Degree	1.586 × 10^−3^	3.757 × 10^−4^	4.222	3.20 × 10^−5^ ***
Betweenness	8.401 × 10^−1^	2.274 × 10^−1^	3.695	0.000260 ***
Clustering	3.327 × 10^−1^	2.390 × 10^−2^	13.920	<2 × 10^−16^ ***
Profitability	−5.858 × 10^−5^	1.600 × 10^−5^	-3.661	0.000296 ***
Productivity	1.543 × 10^−4^	3.697 × 10^−5^	4.174	3.90 × 10^−5^ ***
IN	−1.212 × 10^−3^	7.791 × 10^−3^	-0.156	0.876497
SCC	6.291 × 10^−2^	1.130 × 10^−2^	5.570	5.56 × 10^−8^ ***
SectorCivil	−9.884 × 10^−3^	9.583 × 10^−3^	-1.031	0.303147
SectorGame	−2.341 × 10^−2^	7.083 × 10^−3^	-3.305	0.001061 **

**Table 6:** Regression of profitability for the modern industries. Multiple *R*^2^: 0.03523, Adjusted *R*^2^: 0.003801, F-statistic: 1.121 on 10 and 307 DF, p-value: 0.3458.

Variable	Estimate	Std. Error	t value	*Pr*(>|*t*|) 0 ‘***’ 0.001 ‘**’ 0.01 0.01 ‘*’ 0.05 ‘.’ 0.1
(Intercept)	197.462	271.055	0.728	0.46687
Degree	-2.550	3.283	-0.777	0.43783
Betweenness	1782.111	1971.283	0.904	0.36669
Clustering	-226.287	281.216	-0.805	0.42163
*R*_*lr*_	-256.856	586.130	-0.438	0.66153
*D*_*lr*_	94.602	562.030	0.168	0.86644
*B*_*lr*_	218.468	716.871	0.305	0.76076
IN	-182.258	64.194	-2.839	0.00482 **
SCC	-155.198	99.576	-1.559	0.12013
SectorCivil	-49.613	82.563	-0.601	0.54834
SectorGame	45.819	61.263	0.748	0.45509

**Table 7:** Regression of productivity for the modern industries. Multiple *R*^2^: 0.07086, Adjusted *R*^2^: 0.04059, F-statistic: 2.341 on 10 and 307 DF, p-value: 0.0113.

Variable	Estimate	Std. Error	t value	*Pr*(>|*t*|) 0 ‘***’ 0.001 ‘**’ 0.01 0.01 ‘*’ 0.05 ‘.’ 0.1
(Intercept)	-19.5715	116.5492	-0.168	0.866753
Degree	-0.5346	1.4116	-0.379	0.705158
Betweenness	236.5766	847.6194	0.279	0.780351
Clustering	-132.6638	120.9183	-1.097	0.273442
*R*_*lr*_	-178.1672	252.0265	-0.707	0.480141
*D*_*lr*_	-51.0545	241.6639	-0.211	0.832823
*B*_*lr*_	547.7298	308.2428	1.777	0.076568 .
IN	-105.0540	27.6025	-3.806	0.000171 ***
SCC	-106.4957	42.8162	-2.487	0.013403 *
SectorCivil	-18.9103	35.5009	-0.533	0.594648
SectorGame	28.9234	26.3419	1.098	0.273064

**Table 8:** Regression of *R*_*lr*_ for the traditional craft industries. Multiple *R*^2^: 0.6253, Adjusted *R*^2^: 0.6142, F-statistic: 56.73 on 8 and 272 DF, p-value: <2.2 × 10^−16^.

Variable	Estimate	Std. Error	t value	*Pr*(>|*t*|) 0 ‘***’ 0.001 ‘**’ 0.01 0.01 ‘*’ 0.05 ‘.’ 0.1
(Intercept)	4.664 × 10^−1^	9.233 × 10^−3^	50.510	< 2 × 10^−16^ ***
Degree	1.431 × 10^−2^	9.638 × 10^−4^	14.843	< 2 × 10^−16^ ***
Betweenness	-1.639	3.265 × 10^−1^	-5.021	9.30 × 10^−7^ ***
Clustering	2.541 × 10^−1^	7.765 × 10^−2^	3.272	0.0012 **
OUT	−5.730 × 10^−2^	1.064 × 10^−2^	-5.385	1.57 × 10^−7^ ***
Profitability	1.707 × 10^−4^	1.536 × 10^−4^	1.111	0.2675
Productivity	−4.281 × 10^−6^	1.070 × 10^−4^	-0.040	0.9681
SectorDoll	3.079 × 10^−2^	1.379 × 10^−2^	2.233	0.0263 *
SectorDyeing	1.811 × 10^−2^	1.139 × 10^−2^	1.590	0.1129

**Table 9:** Regression of *D*_*lr*_ for the traditional craft industries. Multiple *R*^2^: 0.4365, Adjusted *R*^2^: 0.4177, F-statistic: 23.32 on 9 and 271 DF, p-value: < 2.2 × 10^−16^.

Variable	Estimate	Std. Error	t value	*Pr*(>|*t*|) 0 ‘***’ 0.001 ‘**’ 0.01 0.01 ‘*’ 0.05 ‘.’ 0.1
(Intercept)	0.3052770	0.0234214	13.034	< 2 × 10^−16^ ***
Degree	0.0087009	0.0015866	5.484	9.53 × 10^−8^ ***
Betweenness	-0.4801660	0.5307414	-0.905	0.3664
Clustering	0.2197414	0.1262494	1.741	0.0829 .
OUT	-0.0264025	0.0247809	-1.065	0.2876
SCC	-0.0102008	0.0242510	-0.421	0.6744
Profitability	0.0001287	0.0002496	0.516	0.6066
Productivity	0.0003381	0.0001738	1.945	0.0528 .
SectorDoll	-0.0184324	0.0225025	-0.819	0.4134
SectorDyeing	0.1727953	0.0185106	9.335	< 2 × 10^−16^ ***

**Table 10:** Regression of *B*_*lr*_ for the traditional craft industries. Multiple *R*^2^: 0.577, Adjusted *R*^2^: 0.563, F-statistic: 41.08 on 9 and 271 DF, p-value: < 2.2 × 10^−16^.

Variable	Estimate	Std. Error	t value	*Pr*(>|*t*|) 0 ‘***’ 0.001 ‘**’ 0.01 0.01 ‘*’ 0.05 ‘.’ 0.1
(Intercept)	0.3534590	0.0203119	17.402	< 2 × 10^−16^ ***
Degree	0.0155917	0.0013760	11.331	< 2 × 10^−16^ ***
Betweenness	-2.5569835	0.4602794	-5.555	6.62 × 10^−8^ ***
Clustering	0.3988484	0.1094883	3.643	0.000323 ***
OUT	0.0282121	0.0214909	1.313	0.190379
SCC	0.1513769	0.0210314	7.198	6.03 × 10^−12^ ***
Profitability	0.0001668	0.0002165	0.771	0.441669
Productivity	0.0001198	0.0001507	0.795	0.427480
SectorDoll	0.0497356	0.0195151	2.549	0.011368 *
SectorDyeing	0.0262411	0.0160531	1.635	0.103285

**Table 11:** Regression of profitability for the traditional craft industries. Multiple *R*^2^: 0.1374, Adjusted *R*^2^: 0.1054, F-statistic: 4.299 on 10 and 270 DF, p-value: 1.397 × 10^−5^.

Variable	Estimate	Std. Error	t value	*Pr*(>|*t*|) 0 ‘***’ 0.001 ‘**’ 0.01 0.01 ‘*’ 0.05 ‘.’ 0.1
(Intercept)	-4.6539	13.8393	-0.336	0.73692
Degree	0.1805	0.5455	0.331	0.74101
Betweenness	220.5631	140.9536	1.565	0.11880
Clustering	-20.8184	32.7608	-0.635	0.52566
*R*_*lr*_	5.1157	54.7777	0.093	0.92566
*D*_*lr*_	15.0200	15.5045	0.969	0.33354
*B*_*lr*_	13.5085	35.9865	0.375	0.70767
OUT	11.2296	6.2817	1.788	0.07495 .
SCC	0.8790	6.7094	0.131	0.89587
SectorDoll	16.8283	5.6801	2.963	0.00332 **
SectorDyeing	2.3994	5.3509	0.448	0.65422

**Table 12:** Regression of productivity for the traditional craft industries. Multiple *R*^2^: 0.0832, Adjusted *R*^2^: 0.04924, F-statistic: 2.45 on 10 and 270 DF, p-value: 0.008157.

Variable	Estimate	Std. Error	t value	*Pr*(>|*t*|) 0 ‘***’ 0.001 ‘**’ 0.01 0.01 ‘*’ 0.05 ‘.’ 0.1
(Intercept)	29.9812	19.6651	1.525	0.1285
Degree	0.5813	0.7751	0.750	0.4540
Betweenness	265.9797	200.2896	1.328	0.1853
Clustering	-46.6428	46.5519	-1.002	0.3173
*R*_*lr*_	-123.7206	77.8370	-1.589	0.1131
*D*_*lr*_	50.8987	22.0313	2.310	0.0216 *
*B*_*lr*_	91.9314	51.1355	1.798	0.0733 .
OUT	5.3547	8.9261	0.600	0.5491
SCC	-2.6067	9.5338	-0.273	0.7847
SectorDoll	-7.8166	8.0712	-0.968	0.3337
SectorDyeing	-13.2364	7.6034	-1.741	0.0829 .

The values of *R*^2^ of the robustness *R*_*lr*_, *D*_*lr*_, *B*_*lr*_ are high, but the profitability and the productivity are low for both the modern industry sectors and the traditional craft industry sectors. The statistically significant explanatory variables of the robustness in the modern industry sectors are Degree (positive), Betweenness (positive), Clustering (positive), Profitability (negative), Productivity (positive), and SCC (positive), where the directions of effect are shown in parentheses. The robustness of the electric machinery industry is higher than the consumer game industry and civil engineering industry. The statistically significant explanatory variables of the robustness in the traditional craft industry sectors are Degree (positive), Betweenness (positive), and Clustering (positive). In addition to these variables, the part of the bow-tie structure is significant; OUT (negative) for *R*_*lr*_, and SCC (positive) for *B*_*lr*_. For *R*_*lr*_ and *B*_*lr*_, the Kyoto doll industry is higher than in other industries. For *D*_*lr*_, the Kyoyuzen dyeing industry is higher than in other industries. The statistically significant explanatory variables of the profitability and the productivity in the modern industry sectors are the part of the bow-tie structure; IN (negative) for profitability and productivity, and SCC (negative) for productivity. The statistically significant explanatory variables of the profitability in the traditional craft industry sectors are OUT (positive). The profitability of the Kyoto doll industry is higher than in other traditional craft industries. The statistically significant explanatory variables of the productivity in the traditional craft industry sectors are *D*_*lr*_ (positive) and *B*_*lr*_ (positive). The productivity of the Kyoyuzen dyeing industry is lower than other traditional craft industries.

Based on the network and econometric analyses, it was clarified that the traditional craft industry has a markedly different network characteristics to more modern industries such as the consumer games and electric machinery industries. According to the centrality analysis, the consumer games and electrical machinery industries, which are the leading industries in Kyoto, have established a central production structure in which a single manufacturer has an unparalleled centrality. Such a structure is considered an advantageous structure for responding to a rapidly changing market because one firm has a consensus on manufacturing and manages every aspect from production to sales, enabling the manufacturers to directly capture the consumers’ needs and immediately respond to them. In contrast, the traditional craft industry has a clear division of labor throughout the process, where wholesale firms continue to play a central role. Such a structure is regarded as having been formed in an age in which good-quality products always sold out. On examining the results related to the network topology characteristics, it became clear that the traditional craft communities have low clustering coefficients than networks with degree-preserving randomization. The traditional craft industry also has issues in exchanging information, while the modern industry has a dense structure with a high clustering coefficient [40]. Furthermore, more modern industries have an SCC within the industrial community, with the attendant firms creating high value-added and playing a major role in driving the entire industry. In contract, the Nishijin silk fabrics and Kyoto doll industries do not have an SCC and have thus not been able to create a feedback loop for the entire industry.

## Conclusion

This research aimed to characterize Kyoto’s traditional craft industry by analyzing the supplier-customer network involving individual firms within the Kyoto region. It became evident that the traditional craft industry has a different network structure from the modern consumer games and electric machinery industries. The modern industries have the strongly coupled component, and the attendant firms there create high value-added and play a significant role in driving the entire industry, while more traditional craft industries, such as the Nishijin silk fabrics and Kyoto doll industries, do not have this strongly coupled component. We constructed econometric models that simultaneously consider the various structural characteristics variables to realize the inter-dependencies among structural characteristics, robustness, profitability, and productivity. The mode revealed that the statistically significant explanatory variables of the robustness, the profitability, the productivity differ in the traditional craft industry and the modern industry. Moreover, the traditional crafts industry does not have a central firm or a dense network for integrating information, which is presumed to be a factor in the decline of the traditional craft industry. The results of this analysis suggest that the main issue lies in the fact that the traditional craft industry in Kyoto does not have a structure to integrate information that will allow it to respond to the markets. In order to take advantage of the strength of a region-based industry, Kyoto’s traditional craft industry must create a production system that accurately reflects the consumer’s voice and carries out it’s manufacturing in close collaboration with the industry as a whole.

## Supporting information

S1 Appendix (PDF)
