# Supplementary material for: The sustainability and the survivability of Kyoto’s traditional craft industry revealed from supplier-customer network
Source: PLoS One. 2020 Nov 9;15(11):e0240618. doi: 10.1371/journal.pone.0240618 (PMC7652274; doi:10.1371/journal.pone.0240618)
Supplement: S1 Appendix — (PDF) [file pone.0240618.s001.pdf]

S1 Appendix: The firms' information of each selected community

- S1,S2,S3,S4,S5,S6 Tables represents the firms' information of each selected community. S1 Table is for Nishijin silk fabrics, S2 Table is for Kyoyuzen dyeing, S3 Table is for Kyoto doll, S4 Table is for consumer games, S5 Table is for electric machinery,and S6 Table is for civil engineering community. firms' information include no.,sector,the number of degree,betweenness centrality,R_{lr},D_{lr},B_{lr},bow-tie component in Kyoto's supplier-customer network,profitability,and productivity of firms in each communities. Only traditional craft industries have "In association" column,which indicates whether the sector of the firm is same as the firm that is belongs to association of each industry. If the value of "In association" column is 1,the firm belongs to the same sector as the firm that is belongs to association of each industry.

S1 TABLE: the firms' information of the Nishijin silk fabric industry (subcommunity 4-1). "-" in some fields indicates missing information

Firm no.	Sector	Degree	Betweenness	Clustering	R_{lr}	D_{lr}	B_{lr}	Bow-tie component (Kyoto)	Profitability	Productivity	In association
1	Textiles, except interior decoration	59	0.0392	0.0	0.92	0.7	0.93	SCC	25.66	40.95	1
2	Office lessors	51	0.023	0.001	0.94	0.77	0.95	SCC	0.27	26.89	1
3	Textiles, except interior decoration	27	0.0062	0.0	0.81	0.61	0.9	SCC	-	-	1
4	Yarn	19	0.0	0.0	0.76	0.68	0.83	IN	50.86	172.08	1
5	Textiles, except interior decoration	17	0.0049	0.0	0.73	0.51	0.81	SCC	-	70.0	1
6	Fiber materials	17	0.0	0.0	0.78	0.76	0.82	SCC	25.12	251.25	0
7	Miscellaneous apparel accessories and notions	14	0.0018	0.0	0.72	0.58	0.78	SCC	-	63.89	1
8	Woven silk and rayon fabrics	13	0.0	0.0	0.71	0.24	0.8	SCC	68.0	97.14	1
9	Woven silk and rayon fabrics	13	0.0048	0.0032	0.7	0.43	0.83	SCC	37.51	12.1	1
10	Yarn	12	0.0032	0.0	0.73	0.32	0.74	SCC	20.0	40.0	1
11	Fabricated metal products, n.e.c.	12	0.001	0.0	0.7	0.09	0.76	SCC	9.97	33.25	1
12	Woven silk and rayon fabrics	12	0.0041	0.0	0.69	0.62	0.77	SCC	61.42	63.54	1
13	Miscellaneous crude textile products	11	0.001	0.0	0.71	0.09	0.75	SCC	3.45	11.5	1
14	Woven silk and rayon fabrics	11	0.0048	0.0	0.7	0.53	0.81	SCC	5.49	11.78	1
15	Miscellaneous apparel accessories and notions	11	0.0017	0.0	0.67	0.57	0.74	SCC	56.0	80.0	1
16	Textiles, except interior decoration	10	0.0012	0.0222	0.67	0.58	0.75	SCC	37.43	44.91	1
17	Woven silk and rayon fabrics	10	0.0028	0.0	0.7	0.54	0.78	SCC	10.65	8.19	1
18	Woven silk and rayon fabrics	10	0.0022	0.0	0.71	0.47	0.79	SCC	2.15	14.33	1
19	Miscellaneous apparel accessories and notions	9	0.0007	0.0	0.67	0.15	0.73	SCC	19.0	38.0	1
20	Japanese style apparel, including Japanese "tabi"-socks	9	0.002	0.0	0.68	0.57	0.75	SCC	40.02	14.29	1
21	Japanese style apparel, including Japanese "tabi"-socks	9	0.0028	0.0	0.69	0.43	0.79	SCC	19.05	33.33	1

22	Miscellaneous woven fabrics	9	0.001	0.0	0.68	0.37	0.73	SCC	34.0	37.78	1
23	Real estate managers	8	0.0006	0.0	0.65	0.14	0.73	SCC	0.17	11.0	0
24	Japanese style apparel, including Japanese "tabi"-socks	8	0.0012	0.0	0.68	0.49	0.76	SCC	12.0	20.0	1
25	Japanese style apparel, including Japanese "tabi"-socks	7	0.0014	0.0	0.64	0.29	0.75	SCC	8.0	16.0	1
26	Japanese style apparel, including Japanese "tabi"-socks	7	0.0007	0.0	0.64	0.33	0.76	SCC	1.67	10.0	1
27	Japanese style apparel, including Japanese "tabi"-socks	7	0.0023	0.0	0.62	0.27	0.69	SCC	6.5	21.67	1
28	Woven silk and rayon fabrics	7	0.001	0.0	0.62	0.43	0.73	SCC	7.12	9.5	1
29	Dyed and finished quasi-cotton fibers and yarns	7	0.0013	0.0	0.6	0.31	0.67	SCC	29.75	11.9	0
30	Woven silk and rayon fabrics	6	0.0006	0.0167	0.64	0.51	0.75	SCC	5.09	15.0	1
31	Woven silk and rayon fabrics	6	0.0004	0.0167	0.61	0.38	0.69	SCC	8.0	6.15	1
32	Textiles, except interior decoration	6	0.0008	0.0	0.62	0.42	0.72	SCC	28.5	47.5	1
33	Japanese style apparel, including Japanese "tabi"-socks	6	0.0007	0.0	0.59	0.4	0.63	SCC	44.37	34.13	1
34	Fulling and dyeing plants	6	0.0024	0.0	0.54	0.33	0.55	SCC	50.5	50.5	0
35	Textiles, except interior decoration	6	0.0	0.0	0.64	0.24	0.71	OUT	7.5	18.76	1
36	Woven silk and rayon fabrics	6	0.0015	0.0	0.65	0.44	0.72	SCC	-	-	1
37	Woven silk and rayon fabrics	6	0.0005	0.0	0.62	0.34	0.73	SCC	44.0	29.33	1
38	Woven silk and rayon fabrics	6	0.0006	0.0	0.57	0.43	0.63	SCC	9.29	17.69	1
39	Twisting yarns, except bulky yarns	6	0.0004	0.0	0.6	0.31	0.61	SCC	-	-	1
40	Woven silk and rayon fabrics	6	0.0002	0.0	0.66	0.34	0.76	SCC	3.5	3.5	1
41	Japanese style apparel, including Japanese "tabi"-socks	6	0.0006	0.0	0.68	0.27	0.75	SCC	-	22.5	1
42	Woven silk and rayon fabrics	5	0.0007	0.0	0.65	0.21	0.7	SCC	2.0	46.0	1
43	Textiles, except interior decoration	5	0.0009	0.0	0.52	0.38	0.58	SCC	13.55	27.1	1
44	Japanese style apparel, including Japanese "tabi"-socks	5	0.0012	0.0	0.62	0.36	0.67	SCC	6.7	6.7	1
45	Miscellaneous wholesale trade, n.e.c.	5	0.0	0.0	0.57	0.09	0.59	SCC	28.58	85.75	1
46	Spinning, man-made staple fiber	5	0.0005	0.0	0.65	0.31	0.71	SCC	3.93	9.82	0

47	Woven silk and rayon fabrics	5	0.0015	0.0	0.62	0.35	0.69	SCC	3.21	27.58	1
48	Miscellaneous apparel accessories and notions	5	0.0003	0.0	0.47	0.27	0.47	SCC	7.81	37.14	1
49	Woven silk and rayon fabrics	5	0.0008	0.0	0.62	0.34	0.71	SCC	16.55	20.69	1
50	Miscellaneous apparel accessories and notions	4	0.0006	0.0	0.61	0.23	0.65	SCC	9.5	47.5	1
51	Textiles, except interior decoration	4	0.0	0.0	0.64	0.54	0.66	SCC	-	-	1
52	Yarn	4	0.0002	0.0	0.58	0.43	0.61	SCC	14.0	35.0	1
53	Japanese style apparel, including Japanese "tabi"-socks	4	0.0002	0.0	0.64	0.65	0.67	SCC	-	3.0	1
54	Woven silk and rayon fabrics	4	0.0004	0.0	0.47	0.31	0.48	SCC	8.3	27.67	1
55	Miscellaneous apparel accessories and notions	4	0.0003	0.0	0.6	0.13	0.63	SCC	15.33	15.33	1
56	Fiber materials	4	0.0	0.0	0.56	0.47	0.56	IN	17.0	42.5	0
57	Japanese style apparel, including Japanese "tabi"-socks	4	0.0002	0.0	0.53	0.3	0.62	SCC	9.05	18.1	1
58	Japanese style apparel, including Japanese "tabi"-socks	4	0.0001	0.0	0.63	0.57	0.66	SCC	3.23	16.15	1
59	Japanese style apparel, including Japanese "tabi"-socks	4	0.0002	0.0	0.56	0.3	0.62	SCC	3.42	13.67	1
60	Yarn	4	0.0004	0.0	0.56	0.13	0.55	SCC	-	23.5	1
61	Woven silk and rayon fabrics	4	0.0002	0.0	0.6	0.24	0.69	SCC	30.0	30.0	1
62	Natural resin and wood chemical products	4	0.0	0.0	0.57	0.16	0.58	SCC	47.34	79.79	0
63	Textiles, except interior decoration	4	0.0002	0.0	0.61	0.2	0.67	SCC	5.0	25.0	1
64	Supplementary tutorial schools	4	0.0	0.0	0.52	0.43	0.61	OUT	41.67	62.5	0
65	Textiles, except interior decoration	4	0.0	0.0	0.53	0.26	0.56	OUT	19.33	48.33	1
66	Japanese style apparel, including Japanese "tabi"-socks	4	0.0001	0.0	0.63	0.24	0.66	SCC	2.03	15.25	1
67	Japanese style apparel, including Japanese "tabi"-socks	4	0.0004	0.0417	0.57	0.58	0.63	SCC	-	40.0	1
68	Textiles, except interior decoration	3	0.0005	0.0	0.38	0.22	0.37	SCC	2.75	6.88	1
69	Textiles, except interior decoration	3	0.0002	0.0	0.56	0.1	0.6	SCC	5.0	16.68	1
70	Japanese style apparel, including Japanese "tabi"-socks	3	0.0002	0.0	0.6	0.22	0.6	SCC	0.6	2.0	1

71	Textiles, except interior decoration	3	0.0001	0.0	0.57	0.22	0.58	SCC	3.01	19.83	1
72	Woven silk and rayon fabrics	3	0.0	0.0	0.55	0.19	0.57	OUT	4.3	21.5	1
73	Miscellaneous apparel accessories and notions	3	0.0	0.0	0.51	0.33	0.55	SCC	-	19.0	1
74	Miscellaneous apparel accessories and notions	3	0.0004	0.0	0.49	0.39	0.5	OUT	-	42.0	1
75	Japanese style apparel, including Japanese "tabi"-socks	3	0.0001	0.0	0.58	0.23	0.62	SCC	36.67	27.5	1
76	Textiles, except interior decoration	3	0.0004	0.0	0.61	0.46	0.62	SCC	-	-	1
77	Japanese style apparel, including Japanese "tabi"-socks	3	0.0001	0.0	0.58	0.34	0.59	SCC	-	10.0	1
78	Yarn	3	0.0004	0.0	0.53	0.12	0.56	SCC	-	14.17	1
79	Agents and brokers	3	0.0017	0.0	0.47	0.48	0.48	SCC	10.62	43.22	0
80	Miscellaneous apparel accessories and notions	3	0.0	0.0	0.4	0.32	0.43	OUT	17.83	23.32	1
81	Sporting goods	3	0.0	0.0	0.46	0.27	0.46	IN	2.57	8.57	0
82	Woven silk and rayon fabrics	3	0.0001	0.0	0.6	0.52	0.62	IN	0.21	2.15	1
83	Textiles, except interior decoration	3	0.0	0.0	0.53	0.49	0.58	SCC	-	-	1
84	House lessors	3	0.0012	0.0	0.46	0.24	0.44	IN	1.21	36.25	1
85	Textiles, except interior decoration	3	0.0	0.0	0.5	0.34	0.54	SCC	1.14	10.01	1
86	Woven silk and rayon fabrics	3	0.0	0.0	0.57	0.54	0.58	SCC	1.38	5.5	1
87	Japanese style apparel, including Japanese "tabi"-socks	3	0.0	0.0	0.61	0.45	0.6	SCC	-	16.25	1
88	Textiles, except interior decoration	3	0.0009	0.0	0.57	0.44	0.59	SCC	9.0	30.0	1
89	Japanese style apparel, including Japanese "tabi"-socks	3	0.0001	0.0	0.56	0.57	0.63	SCC	33.35	333.5	1
90	Fiber materials	3	0.0	0.0	0.49	0.33	0.5	SCC	15.83	79.17	0
91	Woven silk and rayon fabrics	2	0.0	0.0	0.44	0.33	0.44	SCC	6.0	9.0	1
92	Woven silk and rayon fabrics	2	0.0	0.0	0.56	0.58	0.52	SCC	26.68	13.34	1
93	Miscellaneous apparel accessories and notions	2	0.0	0.0	0.56	0.48	0.54	SCC	-	12.25	1
94	Woven silk and rayon fabrics	2	0.0002	0.0	0.56	0.44	0.51	SCC	5.01	15.03	1
95	Textiles, except interior decoration	2	0.0001	0.0	0.46	0.46	0.43	IN	1.5	7.5	1
96	Japanese style apparel, including Japanese "tabi"-socks	2	0.0006	0.0	0.37	0.21	0.37	SCC	5.0	7.14	1
97	Bags and "fukuromono" (small cases)	2	0.0001	0.0	0.41	0.34	0.42	OUT	-	-	0

98	Miscellaneous apparel accessories and notions	2	0.0001	0.0	0.4	0.37	0.33	IN	-	-	1
99	Woven cotton and staple fiber fabrics	2	0.0	0.0	0.44	0.4	0.4	IN	22.22	50.0	1
100	Fiber materials	2	0.0	0.0	0.47	0.55	0.46	IN	-	-	0
101	Woven silk and rayon fabrics	2	0.0	0.0	0.52	0.57	0.52	SCC	3.0	30.0	1
102	Japanese style apparel, including Japanese ”tabi”-socks	2	0.0	0.25	0.5	0.52	0.5	SCC	16.4	20.5	1
103	Miscellaneous apparel	2	0.0002	0.0	0.36	0.21	0.31	SCC	6.29	7.87	1
104	Woven silk and rayon fabrics	2	0.0026	0.0	0.41	0.22	0.36	OUT	-	-	1
105	Textiles, except interior decoration	2	0.0	0.25	0.48	0.45	0.49	OUT	5.6	29.86	1
106	Pure holding companies	2	0.0	0.0	0.48	0.44	0.51	OUT	0.81	73.59	0
107	Textiles, except interior decoration	2	0.0	0.0	0.48	0.19	0.5	OUT	12.54	25.98	1
108	Miscellaneous apparel	2	0.0	0.0	0.37	0.35	0.36	OUT	16.0	53.33	1
109	Yarn	2	0.0001	0.0	0.48	0.1	0.45	SCC	4.0	13.33	1
110	Miscellaneous crude textile products	2	0.0	0.0	0.42	0.33	0.41	SCC	2.39	8.6	1
111	Miscellaneous apparel	2	0.0	0.0	0.37	0.33	0.34	OUT	21.54	29.17	1
112	Miscellaneous apparel	2	0.0	0.0	0.47	0.17	0.48	OUT	17.7	14.16	1
113	Woven silk and rayon fabrics	2	0.0002	0.0	0.49	0.32	0.48	SCC	2.38	5.96	1
114	Dry goods and cloth stores	2	0.0	0.0	0.55	0.46	0.5	OUT	-	13.0	0
115	Ladies’ and children’ s clothing (occidental style)	2	0.0022	0.0	0.4	0.33	0.41	OUT	34.3	40.99	0
116	Textiles, except interior decoration	2	0.0	0.0	0.47	0.5	0.49	OUT	-	-	1
117	Miscellaneous industry machinery and equipment	2	0.0	0.0	0.48	0.38	0.48	SCC	14.83	44.5	0
118	Japanese style apparel, including Japanese ”tabi”-socks	1	0.0	0.0	0.36	0.38	0.34	IN	1.0	4.5	1
119	Japanese style apparel, including Japanese ”tabi”-socks	1	0.0	0.0	0.45	0.5	0.34	OUT	4.55	22.75	1
120	Dry goods and cloth stores	1	0.0	0.0	0.32	0.17	0.21	OUT	3.6	7.2	0
121	Textiles, except interior decoration	1	0.0	0.0	0.38	0.39	0.37	OUT	3.6	18.0	1
122	Sporting goods stores	1	0.0	0.0	0.28	0.21	0.24	IN TE	8.75	35.0	0
123	Textiles, except interior decoration	1	0.0	0.0	0.47	0.39	0.35	OUT	3.6	9.0	1
124	Woven silk and rayon fabrics	1	0.0	0.0	0.36	0.41	0.33	IN TE	22.65	45.3	1
125	Woven silk and rayon fabrics	1	0.0	0.0	0.45	0.35	0.34	IN	4.5	11.25	1
126	Miscellaneous apparel	1	0.0	0.0	0.33	0.43	0.32	OUT	-	16.0	1

127	Fur skins	1	0.0	0.0	0.5	0.31	0.38	IN	0.5	5.0	0
128	Fur skins	1	0.0	0.0	0.42	0.31	0.35	IN	2.3	4.6	0
129	Miscellaneous crude textile products	1	0.0	0.0	0.42	0.26	0.33	IN	3.9	39.0	1
130	Woven silk and rayon fabrics	1	0.0	0.0	0.46	0.38	0.36	SCC	3.62	9.06	1
131	Woven silk and rayon fabrics	1	0.0	0.0	0.41	0.25	0.32	IN	-	-	1
132	Miscellaneous industry machinery and equipment	1	0.0	0.0	0.42	0.33	0.34	IN	19.84	42.52	0
133	Braids	1	0.0	0.0	0.24	0.16	0.2	IN	26.3	12.52	0
134	Dry goods and cloth stores	1	0.0	0.0	0.31	0.18	0.25	OUT	129.87	17.32	0
135	Miscellaneous apparel	1	0.0	0.0	0.31	0.32	0.28	OUT TE	26.0	39.0	1
136	Wooden building work	1	0.0	0.0	0.33	0.2	0.24	IN	-	5.5	0
137	Woven cotton and staple fiber fabrics	1	0.0	0.0	0.32	0.18	0.26	IN	-	-	1
138	Office lessors	1	0.0	0.0	0.35	0.41	0.34	IN	0.81	73.8	1
139	Woven silk and rayon fabrics	1	0.0	0.0	0.24	0.25	0.24	OUT TE	-	-	1
140	Miscellaneous apparel	1	0.0	0.0	0.35	0.34	0.35	OUT	62.1	517.5	1
141	Miscellaneous rolling of non-ferrous metals and alloys, including drawing and extruding	1	0.0	0.0	0.3	0.26	0.25	IN	-	-	0
142	Miscellaneous apparel	1	0.0	0.0	0.36	0.3	0.37	OUT	9.31	21.6	1
143	Textiles, except interior decoration	1	0.0	0.0	0.37	0.32	0.39	OUT	-	50.0	1
144	Miscellaneous apparel	1	0.0	0.0	0.4	0.38	0.35	OUT	6.03	32.17	1
145	Miscellaneous apparel	1	0.0	0.0	0.39	0.31	0.37	OUT	10.0	25.0	1
146	Japanese style apparel, including Japanese ”tabi”-socks	1	0.0	0.0	0.37	0.27	0.3	SCC	12.5	10.5	1
147	Miscellaneous apparel accessories and notions	1	0.0	0.0	0.4	0.31	0.34	SCC	3.0	15.0	1
148	Miscellaneous apparel	1	0.0	0.0	0.38	0.42	0.34	OUT	6.67	28.57	1
149	Textiles, except interior decoration	1	0.0	0.0	0.36	0.28	0.35	OUT	1.25	25.0	1
150	Hand dyed and finished woven fabrics	1	0.0	0.0	0.38	0.31	0.31	OUT	2.05	10.25	0
151	Dry goods and cloth stores	1	0.0	0.0	0.46	0.35	0.34	OUT	6.67	10.0	0
152	Miscellaneous apparel accessories and notions	1	0.0	0.0	0.4	0.34	0.38	OUT	1.25	12.5	1
153	Dry goods and cloth stores	1	0.0	0.0	0.39	0.32	0.36	OUT	-	-	0
154	Miscellaneous apparel	1	0.0	0.0	0.37	0.31	0.37	OUT	44.0	19.13	1
155	Miscellaneous apparel	1	0.0	0.0	0.42	0.32	0.36	OUT	3.0	10.0	1

156	Nonstore retailers (woven fabrics, apparel, apparel accessories and notions)	1	0.0	0.0	0.39	0.3	0.34	OUT	7.0	17.5	0
157	Ladies' and children' s clothing (occidental style)	1	0.0	0.0	0.53	0.39	0.36	OUT	14.0	29.17	0
158	Miscellaneous apparel	1	0.0	0.0	0.42	0.39	0.37	OUT	4.0	13.33	1
159	Miscellaneous apparel	1	0.0	0.0	0.39	0.32	0.35	OUT	6.68	28.63	1
160	Dry goods and cloth stores	1	0.0	0.0	0.29	0.25	0.27	OUT	9.0	45.0	0
161	Dry goods and cloth stores	1	0.0	0.0	0.35	0.28	0.36	OUT	-	-	0
162	Dry goods and cloth stores	1	0.0	0.0	0.3	0.28	0.27	OUT	-	9.0	0
163	Embroidery	1	0.0	0.0	0.3	0.16	0.19	OUT	-	-	0
164	Woven fabrics, apparel, apparel accessories and notions stores, n.e.c.	1	0.0	0.0	0.31	0.24	0.29	OUT	17.5	13.12	1
165	Agents and brokers	1	0.0	0.0	0.44	0.31	0.34	OUT	-	-	0
166	Textiles, except interior decoration	1	0.0	0.0	0.38	0.29	0.34	OUT	-	10.5	1
167	Dry goods and cloth stores	1	0.0	0.0	0.24	0.17	0.22	OUT	6.67	10.0	0
168	Handbags	1	0.0	0.0	0.36	0.28	0.31	OUT	37.5	50.0	0
169	Woven silk and rayon fabrics	1	0.0	0.0	0.43	0.31	0.31	OUT	8.85	11.06	1
170	Textiles, except interior decoration	1	0.0	0.0	0.38	0.38	0.37	OUT	10.0	25.0	1
171	Dry goods and cloth stores	1	0.0	0.0	0.4	0.38	0.35	OUT	-	-	0
172	Common motor trucking, except special group cargo motor trucking	1	0.0	0.0	0.37	0.24	0.29	OUT	-	-	0
173	Miscellaneous apparel accessories and notions	1	0.0	0.0	0.43	0.32	0.38	SCC	4.85	48.5	1
174	Machine dyed and finished woolen and worsted fabrics	1	0.0	0.0	0.39	0.24	0.29	IN	-	-	0

S2 TABLE: the firms' information of the Kyoyuzen dyeing industry (subcommunity 4-6). "-" in some fields indicates missing information

Firm no.	Sector	Degree	Betweenness	Clustering	R_{lr}	D_{lr}	B_{lr}	Bow-tie component (Kyoto)	Profitability	Productivity	In association
1	Fiber materials	68	0.3061	0.0015	0.93	0.91	0.81	SCC	134.0	138.14	0
2	Yarn	41	0.1214	0.0009	0.91	0.86	0.92	SCC	45.12	167.11	1
3	Fiber materials	33	0.1317	0.0019	0.85	0.8	0.88	SCC	42.42	68.42	0
4	Textiles, except interior decoration	14	0.0406	0.0083	0.73	0.62	0.78	SCC	113.11	102.83	1
5	Woven silk and rayon fabrics	7	0.0342	0.0833	0.58	0.6	0.71	SCC	18.04	24.06	1
6	Woven silk and rayon fabrics	7	0.0366	0.0	0.66	0.53	0.72	SCC	4.03	8.06	1
7	Woven silk and rayon fabrics	6	0.0278	0.0	0.59	0.48	0.7	SCC	19.98	16.2	1

8	Textiles, except interior decoration	6	0.0185	0.125	0.54	0.48	0.71	SCC	20.0	33.33	1
9	Hand dyed and finished woven fabrics	5	0.0086	0.1	0.58	0.53	0.64	SCC	15.58	8.2	0
10	Woven silk and rayon fabrics	5	0.0109	0.0	0.57	0.42	0.69	SCC	11.82	130.0	1
11	Machine dyed and finished silk and rayon fabrics	5	0.0149	0.0	0.62	0.34	0.73	SCC	7.33	12.21	0
12	Textiles, except interior decoration	5	0.0113	0.0833	0.61	0.58	0.68	SCC	26.74	77.0	1
13	Textiles, except interior decoration	5	0.0069	0.025	0.59	0.46	0.67	SCC	3.33	50.0	1
14	Woven cotton and staple fiber fabrics	5	0.0125	0.0	0.62	0.51	0.73	SCC	14.67	62.86	1
15	Miscellaneous apparel accessories and notions	5	0.014	0.0	0.63	0.57	0.67	SCC	12.83	48.12	1
16	Miscellaneous apparel	5	0.0387	0.0	0.57	0.46	0.67	SCC	6.0	20.01	1
17	Hand dyed and finished woven fabrics	5	0.0162	0.125	0.57	0.69	0.64	SCC	14.25	8.91	0
18	Hand dyed and finished woven fabrics	4	0.0002	0.0417	0.59	0.64	0.65	SCC	5.25	9.19	0
19	Woven silk and rayon fabrics	4	0.0024	0.1	0.62	0.64	0.6	SCC	11.95	8.53	1
20	Japanese style apparel, including Japanese "tabi"-socks	4	0.0	0.0833	0.58	0.56	0.65	SCC	13.28	44.28	1
21	Miscellaneous woven fabrics	4	0.0	0.0833	0.56	0.7	0.59	SCC	46.5	29.06	1
22	Hand dyed and finished woven fabrics	4	0.0004	0.0417	0.56	0.62	0.6	SCC	-	10.5	0
23	Plastic profile extrusions	4	0.0122	0.0	0.57	0.58	0.63	SCC	16.82	42.04	0
24	Japanese style apparel, including Japanese "tabi"-socks	4	0.0	0.0833	0.58	0.6	0.62	SCC	3.72	24.8	1
25	Woven silk and rayon fabrics	4	0.0017	0.2	0.59	0.66	0.64	SCC	10.17	12.21	1
26	Japanese style apparel, including Japanese "tabi"-socks	4	0.0037	0.0833	0.61	0.57	0.66	SCC	7.08	42.48	1
27	Yarn	3	0.0084	0.0	0.52	0.49	0.49	SCC	-	-	1
28	Textiles, except interior decoration	3	0.0	0.0	0.51	0.62	0.55	OUT	63.23	109.44	1
29	Weaving and knitting machinery	3	0.0016	0.1667	0.58	0.66	0.62	SCC	5.88	6.54	0
30	Surface-active agents, except soaps and synthetic detergent	3	0.0	0.0833	0.6	0.64	0.63	IN	6.17	9.74	0
31	Woven silk and rayon fabrics	3	0.0	0.0	0.58	0.56	0.67	OUT	10.16	9.56	1
32	Woven cotton and staple fiber fabrics	3	0.0008	0.0	0.59	0.56	0.57	OUT	-	-	1
33	Woven silk and rayon fabrics	3	0.0017	0.0	0.54	0.57	0.57	SCC	4.24	10.59	1
34	Woven cotton and staple fiber fabrics	3	0.0017	0.0	0.54	0.56	0.57	SCC	6.67	11.43	1

35	Hand dyed and finished woven fabrics	3	0.0	0.0833	0.58	0.63	0.6	SCC	7.5	18.75	0
36	Hand dyed and finished woven fabrics	3	0.0084	0.25	0.49	0.65	0.52	SCC	23.25	17.88	0
37	Narrow woven fabrics	2	0.0	0.0	0.43	0.45	0.42	SCC	3.03	13.65	1
38	Dry goods and cloth stores	2	0.0	0.0	0.52	0.59	0.57	OUT	40.83	9.42	0
39	Machine dyed and finished silk and rayon fabrics	2	0.0012	0.0	0.52	0.56	0.54	SCC	110.0	47.83	0
40	Dry goods and cloth stores	2	0.0	0.0	0.52	0.57	0.57	OUT	10.6	26.5	0
41	Miscellaneous spinning	2	0.0016	0.0	0.5	0.53	0.49	IN	12.29	6.14	0
42	Dry goods and cloth stores	2	0.0	0.0	0.51	0.6	0.6	OUT	47.5	23.75	0
43	Japanese style apparel, including Japanese ”tabi”-socks	2	0.0	0.25	0.53	0.69	0.49	IN	6.36	9.54	1
44	Textiles, except interior decoration	2	0.0	0.0	0.43	0.43	0.4	SCC	1.57	47.0	1
45	Woven silk and rayon fabrics	2	0.0	0.0	0.52	0.54	0.57	OUT	5.25	10.5	1
46	Woven silk and rayon fabrics	2	0.0	0.0	0.56	0.58	0.58	SCC	29.17	3.62	1
47	Woven silk and rayon fabrics	2	0.0	0.0	0.44	0.44	0.4	SCC	-	40.0	1
48	Miscellaneous woven fabrics	2	0.0	0.0	0.56	0.58	0.58	SCC	17.62	16.58	1
49	Fulling and dyeing plants	2	0.0	0.0	0.48	0.53	0.46	SCC	2.29	8.74	0
50	Textiles, except interior decoration	2	0.0101	0.0	0.53	0.55	0.58	SCC	-	217.85	1
51	Woven silk and rayon fabrics	2	0.0	0.0	0.57	0.59	0.57	SCC	10.5	7.0	1
52	Miscellaneous woven fabrics	2	0.0	0.0	0.49	0.42	0.42	SCC	1.1	18.33	1
53	Costume jewelry and costume accessories, except precious metals and jewelry	2	0.0	0.5	0.55	0.64	0.52	SCC	10.0	16.67	0
54	Japanese style apparel, including Japanese ”tabi”-socks	2	0.0	0.0	0.52	0.41	0.41	SCC	116.67	50.0	1
55	Fulling and dyeing plants	2	0.0	0.25	0.52	0.69	0.51	IN	4.5	11.26	0
56	Garment sewing services and repair shops	1	0.0	0.0	0.41	0.43	0.41	IN	4.5	4.85	0
57	Miscellaneous woven fabrics	1	0.0	0.0	0.48	0.43	0.4	OUT	8.89	40.0	1
58	Textile apparel and accessories, n.e.c.	1	0.0	0.0	0.4	0.44	0.4	OUT	11.25	16.07	0
59	Woven silk and rayon fabrics	1	0.0	0.0	0.43	0.44	0.41	OUT	3.33	6.67	1
60	Japanese style apparel, including Japanese ”tabi”-socks	1	0.0	0.0	0.48	0.45	0.4	SCC	22.0	27.5	1
61	Real estate managers	1	0.0	0.0	0.34	0.31	0.35	IN	2.12	42.5	0

62	Japanese style apparel, including Japanese "tabi"-socks	1	0.0	0.0	0.47	0.42	0.4	IN	10.0	30.0	1
63	Textile apparel and accessories, n.e.c.	1	0.0	0.0	0.51	0.45	0.4	IN	21.0	35.0	0
64	Fiber materials	1	0.0	0.0	0.43	0.43	0.4	IN	65.8	54.83	0
65	Machine dyed and finished silk and rayon fabrics	1	0.0	0.0	0.41	0.42	0.42	IN	22.8	7.12	0
66	Fiber materials	1	0.0	0.0	0.52	0.44	0.42	IN	-	-	0
67	Fiber materials	1	0.0	0.0	0.53	0.45	0.4	OUT	10.0	33.33	0
68	Japanese style apparel, including Japanese "tabi"-socks	1	0.0	0.0	0.46	0.44	0.4	IN	22.5	22.5	1
69	Woven silk and rayon fabrics	1	0.0	0.0	0.33	0.33	0.34	IN	-	-	1
70	Garment sewing services and repair shops	1	0.0	0.0	0.47	0.42	0.4	IN	3.83	11.5	0
71	Hand dyed and finished woven fabrics	1	0.0	0.0	0.38	0.22	0.35	IN	-	5.0	0
72	Japanese style apparel, including Japanese "tabi"-socks	1	0.0	0.0	0.44	0.44	0.4	SCC	9.0	10.0	1
73	Woven cotton and staple fiber fabrics	1	0.0	0.0	0.45	0.43	0.41	OUT	52.89	17.63	1
74	Woven silk and rayon fabrics	1	0.0	0.0	0.42	0.45	0.41	OUT	1.47	26.42	1
75	Woven silk and rayon fabrics	1	0.0	0.0	0.41	0.42	0.41	OUT	22.5	28.12	1
76	Hand dyed and finished woven fabrics	1	0.0	0.0	0.51	0.44	0.39	OUT	2.2	11.0	0
77	Costume rental, except otherwise classified	1	0.0	0.0	0.47	0.39	0.37	OUT	12.25	13.12	0
78	Electrical machinery, equipment and supplies, except electrical household appliances	1	0.0	0.0	0.39	0.39	0.36	SCC	15.01	30.01	0
79	Woven silk and rayon fabrics	1	0.0	0.0	0.41	0.31	0.36	SCC	1.18	5.5	1
80	Textiles, except interior decoration	1	0.0	0.0	0.41	0.43	0.4	OUT	4.56	20.72	1
81	Lace	1	0.0	0.0	0.45	0.42	0.4	OUT	26.0	15.6	0
82	Advertising	1	0.0	0.0	0.53	0.42	0.42	OUT	-	-	0
83	Canvas products	1	0.0	0.0	0.41	0.43	0.42	OUT	35.0	23.33	0
84	Miscellaneous apparel accessories and notions	1	0.0	0.0	0.38	0.43	0.4	OUT	31.85	35.39	1
85	Woven silk and rayon fabrics	1	0.0	0.0	0.38	0.45	0.41	OUT	4.8	16.0	1
86	Dry goods and cloth stores	1	0.0	0.0	0.41	0.45	0.39	OUT	-	-	0
87	Textiles, except interior decoration	1	0.0	0.0	0.55	0.45	0.4	OUT	6.67	10.0	1
88	Blankets	1	0.0	0.0	0.43	0.42	0.41	OUT	34.06	14.81	0
89	Nonstore retailers (general merchandise)	1	0.0	0.0	0.44	0.43	0.4	OUT	134.49	168.12	0

90	Miscellaneous business services, n.e.c.	1	0.0	0.0	0.32	0.27	0.35	OUT	8.0	8.0	0
91	Yarn	1	0.0	0.0	0.4	0.45	0.4	OUT	9.5	23.75	1
92	Yarn	1	0.0	0.0	0.38	0.45	0.4	OUT	8.99	59.93	1
93	Woven silk and rayon fabrics	1	0.0	0.0	0.48	0.45	0.41	OUT	8.0	24.0	1
94	Printing, bookbinding and paper converting machinery	1	0.0	0.0	0.38	0.4	0.34	IN	9.02	9.02	0

S3 TABLE: the firms' information of the Kyoto doll industry (subcommunity 4-25). "-" in some fields indicates missing information

Firm no.	Sector	Degree	Betweenness	Clustering	R_{lr}	D_{lr}	B_{lr}	Bow-tie component (Kyoto)	Profitability	Productivity	In association
1	Textiles, except interior decoration	49	0.0953	0.006	0.95	0.74	0.75	SCC	36.48	32.23	1
2	Amusement goods and toys	21	0.0099	0.0071	0.81	0.65	0.87	OUT	-	-	0
3	Amusement goods and toys	15	0.0055	0.0571	0.76	0.74	0.85	OUT	180.0	41.54	0
4	Woven silk and rayon fabrics	13	0.0159	0.0227	0.71	0.51	0.75	OUT	55.0	36.67	1
5	Dolls	11	0.0009	0.0591	0.71	0.04	0.8	OUT	115.0	28.75	0
6	Dolls	8	0.0054	0.0268	0.66	0.52	0.7	OUT	25.67	12.83	0
7	Dolls	8	0.0	0.0625	0.62	0.05	0.73	OUT	23.0	31.94	0
8	Dolls	8	0.0063	0.0179	0.65	0.42	0.74	OUT	17.0	17.0	0
9	Amusement goods and toys	7	0.0	0.0714	0.66	0.09	0.72	OUT	37.88	25.25	0
10	Dolls	7	0.0027	0.0595	0.66	0.47	0.74	OUT	23.6	18.88	0
11	Miscellaneous crude textile products	6	0.0124	0.0	0.52	0.29	0.54	SCC	8.22	15.34	1
12	Toy and amusement goods stores	5	0.0001	0.125	0.62	0.11	0.71	OUT	58.33	29.17	0
13	Textiles, except interior decoration	5	0.0037	0.0	0.55	0.37	0.59	OUT	-	-	1
14	Dolls	5	0.0008	0.1	0.6	0.38	0.71	OUT	16.95	22.6	0
15	Dolls	5	0.0034	0.025	0.6	0.4	0.65	OUT	-	2.25	0
16	Dolls	5	0.0097	0.1111	0.58	0.19	0.62	OUT	140.05	21.55	0
17	Dolls	4	0.0	0.125	0.53	0.02	0.62	OUT	14.55	14.55	0
18	Dolls	4	0.0001	0.1667	0.6	0.24	0.67	OUT	26.74	17.83	0
19	Dolls	4	0.0	0.1667	0.54	0.02	0.62	OUT	10.5	6.3	0
20	Dolls	4	0.0003	0.1667	0.56	0.03	0.64	OUT	-	-	0
21	Toy and amusement goods stores	3	0.0	0.1667	0.56	0.22	0.57	OUT	125.5	24.13	0
22	Dolls	3	0.0001	0.0833	0.55	0.1	0.61	OUT	14.29	100.0	0
23	Games and toys, except dolls	3	0.0	0.1667	0.59	0.58	0.6	OUT	45.45	30.3	0
24	Toy and amusement goods stores	3	0.0	0.0	0.54	0.51	0.54	OUT	78.5	23.79	0
25	Dolls	3	0.0001	0.0833	0.53	0.5	0.61	OUT	31.0	77.5	0
26	Dolls	3	0.0	0.0833	0.49	0.53	0.55	OUT TE	17.0	21.25	0
27	Toy and amusement goods stores	3	0.0001	0.0833	0.53	0.11	0.6	OUT	4.0	40.0	0
28	Dolls	3	0.0001	0.0833	0.5	0.51	0.58	OUT	33.0	41.25	0
29	Toy and amusement goods stores	3	0.0	0.1667	0.54	0.23	0.57	OUT	40.0	16.8	0
30	Games and toys, except dolls	3	0.0083	0.1667	0.59	0.26	0.6	OUT	41.25	9.71	0
31	Dolls	3	0.0008	0.0	0.57	0.48	0.59	OUT	22.0	12.22	0
32	Dolls	3	0.0	0.1667	0.56	0.22	0.6	OUT	13.5	33.75	0

33	Dolls	3	0.0001	0.0833	0.54	0.24	0.6	OUT	13.0	14.44	0
34	Dolls	3	0.0006	0.1667	0.52	0.03	0.59	OUT	3.07	11.5	0
35	Dolls	2	0.0001	0.0	0.51	0.48	0.55	OUT	20.0	22.22	0
36	Woven silk and rayon fabrics	2	0.0	0.0	0.44	0.18	0.38	OUT	28.5	28.5	1
37	Amusement goods and toys	2	0.0	0.0	0.53	0.4	0.5	OUT	20.0	25.0	0
38	Woven silk and rayon fabrics	2	0.0056	0.0	0.46	0.27	0.47	SCC	7.38	29.5	1
39	Woven silk and rayon fabrics	2	0.0056	0.0	0.45	0.25	0.46	SCC	15.0	15.0	1
40	Woven silk and rayon fabrics	2	0.0056	0.0	0.45	0.24	0.47	SCC	4.62	52.35	1
41	Toy and amusement goods stores	2	0.0	0.0	0.52	0.46	0.49	OUT	37.5	50.0	0
42	Dolls	2	0.0001	0.0	0.51	0.48	0.52	OUT	16.67	30.0	0
43	Toy and amusement goods stores	2	0.0	0.0	0.5	0.39	0.47	OUT	24.26	38.81	0
44	Dolls	2	0.0	0.25	0.5	0.46	0.51	OUT	46.67	70.0	0
45	Woven silk and rayon fabrics	2	0.0	0.0	0.46	0.32	0.49	IN	9.03	36.1	1
46	Dolls	2	0.0	0.25	0.53	0.07	0.5	OUT	6.77	11.28	0
47	Toy and amusement goods stores	2	0.0	0.0	0.54	0.49	0.54	OUT	75.0	28.85	0
48	Dolls	2	0.0001	0.0	0.5	0.48	0.53	OUT	7.0	23.33	0
49	Toy and amusement goods stores	2	0.0	0.0	0.49	0.4	0.51	OUT	23.06	15.37	0
50	Advertising	1	0.0	0.0	0.41	0.31	0.33	OUT	-	-	0
51	Woven silk and rayon fabrics	1	0.0	0.0	0.35	0.27	0.3	OUT	100.0	15.0	1
52	Toy and amusement goods stores	1	0.0	0.0	0.41	0.27	0.31	OUT	-	-	0
53	Woven silk and rayon fabrics	1	0.0	0.0	0.36	0.3	0.34	OUT	20.8	26.0	1
54	Miscellaneous woven fabrics	1	0.0	0.0	0.31	0.21	0.27	OUT	-	-	1
55	Miscellaneous crude textile products	1	0.0	0.0	0.32	0.21	0.28	IN	5.0	12.5	1
56	Building reform work	1	0.0	0.0	0.43	0.33	0.36	IN	2.44	12.2	0
57	Dolls	1	0.0	0.0	0.42	0.32	0.33	OUT TE	7.17	7.17	0
58	Amusement goods and toys	1	0.0	0.0	0.38	0.25	0.33	OUT	24.0	12.0	0
59	Textiles, except interior decoration	1	0.0	0.0	0.38	0.35	0.34	IN	20.0	32.26	1
60	Dolls	1	0.0	0.0	0.41	0.33	0.38	OUT	-	-	0
61	Blinds for windows and doors and Japanese"byobu"-screens	1	0.0	0.0	0.4	0.34	0.37	OUT	30.0	20.0	0
62	Haberdashery, apparel accessories and notions stores	1	0.0	0.0	0.39	0.34	0.38	OUT	3.69	5.27	0
63	Haberdashery, apparel accessories and notions stores	1	0.0	0.0	0.42	0.34	0.37	OUT	333.33	71.43	0
64	Dolls	1	0.0	0.0	0.41	0.34	0.38	OUT	10.0	8.33	0
65	Games and toys, except dolls	1	0.0	0.0	0.41	0.34	0.37	OUT	6.7	13.4	0
66	Dolls	1	0.0	0.0	0.53	0.33	0.38	OUT	7.0	35.0	0
67	Finishing	1	0.0	0.0	0.42	0.33	0.37	OUT	8.07	8.07	0
68	Toy and amusement goods stores	1	0.0	0.0	0.5	0.35	0.36	OUT	31.15	31.15	0
69	Textiles, except interior decoration	1	0.0	0.0	0.44	0.35	0.38	SCC	63.02	86.79	1

70	Dolls	1	0.0	0.0	0.45	0.33	0.37	OUT	15.2	10.86	0
71	Bags and "fukuromono" (small cases)	1	0.0	0.0	0.36	0.13	0.2	OUT TE	5.69	18.96	0

S4 TABLE: the firms' information of the consumer games industry (subcommunity 14-1). "-" in some fields indicates missing information

Firm no.	Sector	Degree	Betweenness	Clustering	R_{lr}	D_{lr}	B_{lr}	Bow-tie component (Kyoto)	Profitability	Productivity
1	Games and toys, except dolls	227	0.2698	0.0012	0.99	0.98	0.99	SCC	37.1	180.74
2	Amusement goods and toys	9	0.0002	0.0694	0.66	0.65	0.76	OUT	914.79	522.74
3	Embedded software services	8	0.0019	0.1111	0.64	0.59	0.74	SCC	2.92	26.59
4	Miscellaneous business services, n.e.c.	8	0.0036	0.0926	0.62	0.64	0.75	OUT	60.21	169.23
5	Game software services	8	0.0	0.1296	0.66	0.64	0.73	SCC	7.25	17.44
6	Miscellaneous electronic equipment	8	0.0034	0.0741	0.66	0.47	0.66	SCC	7.36	149.92
7	Game software services	7	0.0052	0.1579	0.64	0.62	0.71	SCC	-	-
8	Information recording materials, except newspapers, books, other printed products, etc.	7	0.0	0.125	0.63	0.64	0.73	SCC	22.7	47.52
9	Miscellaneous services incidental to video picture information, sound information, character information production and distribution	5	0.0	0.15	0.62	0.61	0.72	IN	43.06	7.6
10	Game software services	5	0.0	0.2222	0.58	0.68	0.7	SCC	24.72	26.49
11	Game software services	5	0.0	0.1667	0.6	0.64	0.66	SCC	2.47	11.71
12	Worker dispatching services	5	0.0	0.15	0.61	0.64	0.69	IN	98.28	39.31
13	Information recording materials, except newspapers, books, other printed products, etc.	5	0.0016	0.1944	0.58	0.68	0.69	IN	-	-
14	Amusement goods and toys	5	0.0	0.2222	0.61	0.58	0.69	IN	136.59	556.48
15	Amusement goods and toys	5	0.0	0.125	0.58	0.61	0.7	OUT	696.24	729.4
16	Internet support services	5	0.0	0.125	0.62	0.61	0.7	IN	29.73	431.33
17	Game software services	4	0.0036	0.125	0.6	0.65	0.65	OUT	17.83	8.92
18	Embedded software services	4	0.0	0.2	0.58	0.71	0.64	SCC	0.55	60.0
19	Professional services, n.e.c.	4	0.0	0.2	0.5	0.64	0.58	SCC	-	-
20	Game software services	4	0.0	0.2	0.54	0.64	0.62	SCC	45.7	65.29

21	Sporting goods	4	0.0	0.125	0.57	0.6	0.66	OUT	226.38	565.96
22	Information recording materials, except newspapers, books, other printed products, etc.	4	0.0	0.125	0.62	0.64	0.67	IN	14.71	23.29
23	Translation services, except authors	4	0.0	0.2083	0.62	0.66	0.65	IN	51.35	15.1
24	Information recording materials, except newspapers, books, other printed products, etc.	4	0.0	0.2	0.57	0.68	0.6	SCC	32.24	97.86
25	Design services	4	0.0	0.1667	0.61	0.66	0.68	IN	123.62	18.54
26	Information recording materials, except newspapers, books, other printed products, etc.	4	0.0017	0.1667	0.59	0.57	0.68	IN	-	-
27	Games and toys, except dolls	4	0.0	0.125	0.57	0.63	0.67	IN	-	-
28	Wiring devices and supplies	3	0.0	0.25	0.48	0.6	0.52	SCC	3.62	56.92
29	Game software services	3	0.0	0.3333	0.57	0.71	0.62	IN	-	-
30	Game software services	3	0.0	0.1667	0.59	0.7	0.63	IN	-	-
31	Amusement machines	3	0.0	0.1667	0.59	0.7	0.61	IN	2.58	-
32	Information recording materials, except newspapers, books, other printed products, etc.	3	0.0	0.25	0.53	0.64	0.6	IN	17.53	14.61
33	Games and toys, except dolls	3	0.0	0.25	0.6	0.71	0.63	IN	100.0	11.63
34	Game software services	3	0.0	0.1667	0.57	0.69	0.64	IN	27.33	18.14
35	Toy and amusement goods stores	3	0.0	0.25	0.56	0.62	0.64	OUT	5.07	8.44
36	Game software services	3	0.0	0.1667	0.55	0.66	0.59	IN	108.0	23.04
37	Custom software services	2	0.0	0.25	0.52	0.61	0.52	IN	0.63	17.11
38	Custom software services	2	0.0	0.25	0.55	0.64	0.56	IN	-	-
39	Game software services	2	0.0	0.25	0.52	0.62	0.57	IN	21.31	15.83
40	Miscellaneous peripheral equipment	2	0.0	0.5	0.56	0.51	0.56	IN	63.41	10.57
41	Custom software services	2	0.0	0.5	0.52	0.6	0.58	IN	25.77	6.29
42	Game software services	2	0.0	0.5	0.52	0.6	0.58	IN	2.03	10.14
43	Electrical machinery, equipment and supplies, except electrical household appliances	2	0.0	0.25	0.52	0.61	0.52	IN	11.16	89.1
44	Game software services	2	0.0	0.25	0.5	0.6	0.53	IN	-	-

45	Information recording materials, except newspapers, books, other printed products, etc.	2	0.0	0.25	0.52	0.66	0.55	IN	5.08	20.0
46	Pattern manufactured of industrial use	2	0.0	0.5	0.57	0.5	0.57	IN	129.23	25.85
47	Information services, except marketing or opinion research services	2	0.0	0.25	0.51	0.62	0.54	IN	-	-
48	Game software services	2	0.0	0.5	0.52	0.67	0.54	IN	-	-
49	Game software services	2	0.0	0.5	0.49	0.61	0.59	IN	37.0	8.69
50	Data processing services	2	0.0	0.5	0.53	0.61	0.57	IN	37.26	33.18
51	Furniture and fixtures, n.e.c.	2	0.0	0.0	0.53	0.43	0.44	SCC	8.45	-
52	Game software services	2	0.0	0.25	0.51	0.61	0.53	IN	291.74	29.17
53	Electrical machinery, equipment and supplies, except electrical household appliances	2	0.0	0.25	0.49	0.63	0.58	IN	109.72	219.43
54	Game software services	2	0.0	0.25	0.47	0.6	0.53	IN	17.23	19.8
55	Miscellaneous electronic parts, devices and electronic circuits	2	0.0	0.5	0.51	0.6	0.53	IN	-	-
56	Information recording materials, except newspapers, books, other printed products, etc.	2	0.0	0.25	0.49	0.6	0.52	IN	2.3	3.0
57	Custom software services	2	0.0	0.25	0.52	0.6	0.52	IN	21.11	5.63
58	Custom software services	2	0.0	0.25	0.48	0.61	0.53	IN	6.05	8.07
59	Amusement goods and toys	2	0.0	0.25	0.48	0.59	0.57	IN	68.0	48.57
60	Embedded software services	2	0.0	0.25	0.51	0.64	0.56	IN	-	-
61	Bags and "fukuromono" (small cases)	2	0.0	0.5	0.55	0.52	0.58	IN	55.68	83.53
62	Game software services	2	0.0	0.5	0.53	0.58	0.59	IN	3.0	40.0
63	Information recording materials, except newspapers, books, other printed products, etc.	2	0.0	0.5	0.52	0.61	0.58	IN	33.09	33.09
64	Game software services	2	0.0	0.0	0.43	0.44	0.44	SCC	9.55	18.85
65	Game software services	2	0.0	0.25	0.53	0.6	0.59	IN	4.3	19.53
66	Toy and amusement goods stores	2	0.0	0.25	0.56	0.61	0.53	OUT	100.0	30.0
67	Custom software services	2	0.0	0.5	0.53	0.61	0.58	IN	6.24	7.63

68	Embedded software services	2	0.0	0.25	0.53	0.62	0.53	IN	12.0	18.62
69	Amusement goods and toys	2	0.0	0.25	0.56	0.6	0.59	OUT	-	-
70	Custom software services	2	0.0	0.0	0.46	0.43	0.45	SCC	13.56	7.14
71	Professional services, n.e.c.	2	0.0	0.0	0.46	0.45	0.44	SCC	-	-
72	Miscellaneous wholesale trade, n.e.c.	2	0.0	0.25	0.48	0.61	0.57	OUT	53.4	90.55
73	Amusement goods and toys	2	0.0	0.5	0.46	0.63	0.54	OUT	60.11	156.07
74	Amusement goods and toys	2	0.0	0.25	0.55	0.61	0.52	OUT	95.5	73.46
75	Toy and amusement goods stores	2	0.0	0.25	0.57	0.6	0.58	OUT	25.75	36.79
76	Custom software services	2	0.0	0.25	0.52	0.63	0.55	OUT	2.95	12.5
77	Fabrication of foamed and reinforced plastic products	2	0.0	0.5	0.57	0.53	0.56	IN	82.01	32.8
78	Personal computer	2	0.0	0.0	0.54	0.43	0.46	SCC	70.0	28.0
79	Games and toys, except dolls	2	0.0	0.0	0.51	0.44	0.45	SCC	23.15	-
80	Toy and amusement goods stores	2	0.0	0.25	0.56	0.59	0.59	OUT	52.6	51.55
81	Amusement goods and toys	2	0.0	0.5	0.51	0.62	0.57	OUT	1.33	13.28
82	Amusement goods and toys	2	0.0	0.25	0.55	0.64	0.57	OUT	146.84	869.06
83	Amusement goods and toys	2	0.0	0.5	0.47	0.64	0.54	OUT	94.15	76.89
84	Custom software services	1	0.0	0.0	0.43	0.45	0.43	IN	-	-
85	Game software services	1	0.0	0.0	0.45	0.43	0.45	IN	-	-
86	Information recording materials, except newspapers, books, other printed products, etc.	1	0.0	0.0	0.42	0.43	0.45	IN	-	-
87	Custom software services	1	0.0	0.0	0.45	0.44	0.43	IN	35.53	6.75
88	Custom software services	1	0.0	0.0	0.46	0.44	0.44	IN	24.13	35.84
89	Game software services	1	0.0	0.0	0.41	0.43	0.43	IN	-	-
90	Common motor trucking, except special group cargo motor trucking	1	0.0	0.0	0.47	0.44	0.44	IN	55.62	10.11
91	Game software services	1	0.0	0.0	0.46	0.44	0.44	IN	-	-
92	Embedded software services	1	0.0	0.0	0.44	0.44	0.45	IN	0.32	9.5
93	Professional services, n.e.c.	1	0.0	0.0	0.45	0.43	0.44	IN	0.14	3.06
94	Connectors, switches and relays	1	0.0	0.0	0.48	0.43	0.44	IN	23.54	101.23
95	Game software services	1	0.0	0.0	0.49	0.43	0.44	IN	36.0	10.8
96	Edge tools for machinery	1	0.0	0.0	0.52	0.43	0.46	IN	-	-

97	Miscellaneous electronic parts, devices and electronic circuits	1	0.0	0.0	0.5	0.44	0.45	IN	22.25	10.59
98	General goods leasing	1	0.0	0.0	0.51	0.43	0.45	IN	20.8	20.39
99	Custom software services	1	0.0	0.0	0.41	0.43	0.45	IN	20.65	17.07
100	Offset printing in paper	1	0.0	0.0	0.52	0.43	0.45	IN	159.0	26.5
101	Wiring devices and supplies	1	0.0	0.0	0.47	0.42	0.45	IN	-	-
102	Custom software services	1	0.0	0.0	0.42	0.44	0.45	IN	60.15	55.7
103	Information recording materials, except newspapers, books, other printed products, etc.	1	0.0	0.0	0.46	0.43	0.44	IN	5.0	5.0
104	Optical discs and magnetic tapes and discs	1	0.0	0.0	0.51	0.43	0.45	IN	-	-
105	Custom software services	1	0.0	0.0	0.47	0.43	0.45	IN	13.1	13.75
106	Professional services, n.e.c.	1	0.0	0.0	0.43	0.45	0.47	IN	0.33	7.41
107	Game software services	1	0.0	0.0	0.47	0.44	0.45	IN	3.95	9.63
108	Package software services	1	0.0	0.0	0.48	0.4	0.46	IN	43.95	6.98
109	Games and toys, except dolls	1	0.0	0.0	0.51	0.45	0.46	IN	3.33	8.33
110	Custom software services	1	0.0	0.0	0.43	0.43	0.46	IN	-	-
111	Custom software services	1	0.0	0.0	0.48	0.44	0.43	IN	40.9	409.0
112	Information recording materials, except newspapers, books, other printed products, etc.	1	0.0	0.0	0.51	0.44	0.45	IN	33.33	8.89
113	Commodity inspection services	1	0.0	0.0	0.47	0.33	0.39	IN	4.15	13.83
114	Custom software services	1	0.0	0.0	0.4	0.43	0.45	IN	2.0	6.67
115	Game software services	1	0.0	0.0	0.45	0.44	0.44	IN	66.73	14.3
116	Game software services	1	0.0	0.0	0.42	0.4	0.46	IN	30.02	16.45
117	Miscellaneous wholesale trade, n.e.c.	1	0.0	0.0	0.52	0.44	0.46	IN	53.66	67.08
118	Entertainment and sports companies	1	0.0	0.0	0.43	0.41	0.44	IN	2.42	4.84
119	Machine and equipment installation work, except lifting-carrier equipment work	1	0.0	0.0	0.51	0.44	0.44	IN	62.44	36.02
120	Electric measuring instruments, except otherwise classified	1	0.0	0.0	0.42	0.44	0.42	IN	23.35	15.57
121	Custom software services	1	0.0	0.0	0.45	0.42	0.46	IN	-	-

122	Custom software services	1	0.0	0.0	0.44	0.42	0.44	IN	7.36	11.04
123	Custom software services	1	0.0	0.0	0.4	0.44	0.44	IN	1.5	7.5
124	Package software services	1	0.0	0.0	0.4	0.43	0.45	IN	-	-
125	Custom software services	1	0.0	0.0	0.45	0.45	0.44	IN	-	-
126	Custom software services	1	0.0	0.0	0.5	0.44	0.43	IN	10.33	3.88
127	Office machinery of electric appliance stores, except secondhand goods	1	0.0	0.0	0.46	0.43	0.45	IN	-	-
128	Custom software services	1	0.0	0.0	0.48	0.43	0.45	IN	-	-
129	Information recording materials, except newspapers, books, other printed products, etc.	1	0.0	0.0	0.5	0.43	0.43	IN	-	-
130	Embedded software services	1	0.0	0.0	0.44	0.44	0.46	IN	-	-
131	Miscellaneous electronic parts, devices and electronic circuits	1	0.0	0.0	0.49	0.43	0.45	IN	1.06	6.37
132	Patent attorneys' offices	1	0.0	0.0	0.47	0.43	0.44	IN	5.77	9.16
133	Paperboard boxes, cups and plates	1	0.0	0.0	0.53	0.43	0.45	IN	79.55	23.03
134	Miscellaneous electronic parts, devices and electronic circuits	1	0.0	0.0	0.48	0.44	0.45	IN	80.55	80.55
135	Games and toys, except dolls	1	0.0	0.0	0.49	0.44	0.45	IN	37.03	11.82
136	Miscellaneous welfare services for the aged and care services	1	0.0	0.0	0.55	0.43	0.43	IN	1.88	7.5
137	General sawing and planing wood	1	0.0	0.0	0.53	0.43	0.45	IN	10.0	5.88
138	Newspaper publishers	1	0.0	0.0	0.44	0.43	0.47	IN	7.0	15.56
139	Toy and amusement goods stores	1	0.0	0.0	0.42	0.44	0.47	OUT	47.11	47.11
140	Books and magazines	1	0.0	0.0	0.4	0.45	0.46	OUT	17.75	29.58
141	Amusement goods and toys	1	0.0	0.0	0.42	0.44	0.46	OUT	-	-
142	Game centers	1	0.0	0.0	0.42	0.43	0.43	OUT	-	-
143	Amusement goods and toys	1	0.0	0.0	0.42	0.42	0.46	OUT	10.0	33.33
144	Amusement goods and toys	1	0.0	0.0	0.56	0.43	0.44	OUT	381.77	20.64
145	Fuel stores, except gasoline stations	1	0.0	0.0	0.56	0.42	0.43	OUT	16.92	56.41
146	Amusement goods and toys	1	0.0	0.0	0.42	0.42	0.43	OUT	8.29	13.82
147	Cultural organizations	1	0.0	0.0	0.45	0.43	0.45	OUT	-	38.94
148	Amusement goods and toys	1	0.0	0.0	0.53	0.44	0.44	OUT	12.5	125.0

149	Amusement goods and toys	1	0.0	0.0	0.49	0.43	0.44	OUT	1150.0	86.25
150	Amusement goods and toys	1	0.0	0.0	0.51	0.43	0.43	OUT	359.0	143.6
151	Game software services	1	0.0	0.0	0.47	0.44	0.45	OUT	27.4	27.4
152	Bags and "fukuromono" (small cases)	1	0.0	0.0	0.52	0.45	0.46	OUT	21.88	40.51
153	"Aramono"(kitchenware)	1	0.0	0.0	0.54	0.43	0.44	OUT	28.0	280.0
154	Design services	1	0.0	0.0	0.42	0.43	0.44	OUT	32.34	34.4
155	Miscellaneous business services, n.e.c.	1	0.0	0.0	0.41	0.44	0.44	OUT	200.0	50.0
156	Amusement goods and toys	1	0.0	0.0	0.55	0.42	0.45	OUT	-	-
157	Office lessors	1	0.0	0.0	0.55	0.45	0.45	OUT	59.0	59.0
158	Amusement goods and toys	1	0.0	0.0	0.42	0.43	0.46	OUT	841.67	420.83
159	Toy and amusement goods stores	1	0.0	0.0	0.56	0.44	0.46	OUT	-	4.25
160	Miscellaneous wholesale trade, n.e.c.	1	0.0	0.0	0.55	0.45	0.44	OUT	61.47	75.32
161	Toy and amusement goods stores	1	0.0	0.0	0.42	0.44	0.43	OUT	-	36.25
162	Game software services	1	0.0	0.0	0.42	0.44	0.45	OUT	31.76	12.7
163	Amusement goods and toys	1	0.0	0.0	0.56	0.43	0.44	OUT	29.5	24.58
164	Amusement goods and toys	1	0.0	0.0	0.5	0.43	0.47	OUT	12.0	60.0
165	Miscellaneous wholesale trade, n.e.c.	1	0.0	0.0	0.46	0.44	0.44	OUT	7963.65	3185.46
166	Amusement goods and toys	1	0.0	0.0	0.58	0.42	0.44	OUT	232.38	101.04
167	Games and toys, except dolls	1	0.0	0.0	0.41	0.43	0.44	OUT	94.07	45.68
168	Publishers, except newspapers	1	0.0	0.0	0.43	0.45	0.44	OUT	-	-
169	Amusement goods and toys	1	0.0	0.0	0.42	0.42	0.44	OUT	33.3	48.34
170	Amusement goods and toys	1	0.0	0.0	0.59	0.42	0.44	OUT	20.0	25.0
171	Soft drinks and carbonated water, except otherwise classified	1	0.0	0.0	0.56	0.43	0.45	OUT	41.0	51.25
172	Toy and amusement goods stores	1	0.0	0.0	0.42	0.43	0.44	OUT	22.11	49.75
173	Sporting and athletic goods	1	0.0	0.0	0.48	0.43	0.44	OUT	18.0	18.0
174	Toy and amusement goods stores	1	0.0	0.0	0.41	0.43	0.44	OUT	-	2.75
175	Non-life insurance agents and brokers	1	0.0	0.0	0.55	0.44	0.44	SCC	-	-
176	Embedded software services	1	0.0	0.0	0.46	0.43	0.46	IN	14.05	8.78
177	Information recording materials, except newspapers, books, other printed products, etc.	1	0.0	0.0	0.46	0.43	0.44	IN	6.09	9.14
178	Amusement goods and toys	1	0.0	0.0	0.4	0.45	0.43	OUT	13.5	16.88
179	Game centers	1	0.0	0.0	0.55	0.44	0.43	IN	20.69	105.5

180	Amusement goods and toys	1	0.0	0.0	0.48	0.43	0.44	IN	12.59	4.26
181	Information recording materials, except newspapers, books, other printed products, etc.	1	0.0	0.0	0.57	0.43	0.45	IN	21.54	7.43
182	Plastic profile extrusions	1	0.0	0.0	0.49	0.42	0.45	IN	-	-
183	Custom software services	1	0.0	0.0	0.44	0.4	0.45	IN	-	-
184	Professional services, n.e.c.	1	0.0	0.0	0.45	0.44	0.44	IN	5.87	25.68
185	Electrical machinery, equipment and supplies, except electrical household appliances	1	0.0	0.0	0.41	0.44	0.45	IN	1.78	70.86
186	Amusement goods and toys	1	0.0	0.0	0.44	0.45	0.43	IN	2.79	15.4
187	Information recording materials, except newspapers, books, other printed products, etc.	1	0.0	0.0	0.42	0.42	0.43	IN	8.95	9.94
188	Nonstore retailers (miscellaneous goods)	1	0.0	0.0	0.43	0.44	0.44	IN	-	-
189	Data processing services	1	0.0	0.0	0.5	0.42	0.44	IN	8.52	9.46
190	Custom software services	1	0.0	0.0	0.4	0.42	0.45	IN	34.75	18.99
191	Information recording materials, except newspapers, books, other printed products, etc.	1	0.0	0.0	0.42	0.44	0.45	IN	-	-
192	Package software services	1	0.0	0.0	0.42	0.43	0.43	IN	3.0	6.0
193	Advertising	1	0.0	0.0	0.49	0.42	0.44	IN	29.41	26.74
194	Motion picture and video production, except television program and animation production	1	0.0	0.0	0.45	0.44	0.43	IN	21.67	-
195	Game software services	1	0.0	0.0	0.52	0.44	0.44	IN	141.97	10.52
196	Radio program production	1	0.0	0.0	0.47	0.44	0.45	IN	21.8	13.7
197	Information recording materials, except newspapers, books, other printed products, etc.	1	0.0	0.0	0.44	0.46	0.45	IN	-	-
198	Custom software services	1	0.0	0.0	0.44	0.43	0.45	IN	19.75	14.89
199	Custom software services	1	0.0	0.0	0.43	0.44	0.44	IN	-	-
200	Internet support services	1	0.0	0.0	0.55	0.45	0.44	IN	5.28	11.12
201	Game software services	1	0.0	0.0	0.48	0.43	0.45	IN	15.07	5.65

202	Information services, except marketing or opinion research services	1	0.0	0.0	0.51	0.44	0.45	IN	3.57	2.75
203	Non-profit organizations, n.e.c.	1	0.0	0.0	0.42	0.43	0.44	IN	-	61.31
204	Custom software services	1	0.0	0.0	0.45	0.44	0.43	IN	105.0	13.12
205	Custom software services	1	0.0	0.0	0.44	0.44	0.45	IN	19.64	11.79
206	Mechanical design services	1	0.0	0.0	0.48	0.32	0.37	IN	14.38	14.38
207	Miscellaneous data processing and information services	1	0.0	0.0	0.47	0.44	0.45	IN	23.33	17.5
208	Custom software services	1	0.0	0.0	0.48	0.43	0.44	IN	31.33	4.27
209	Information recording materials, except newspapers, books, other printed products, etc.	1	0.0	0.0	0.42	0.44	0.42	IN	8.0	13.33
210	Electrical appliance stores, except secondhand goods	1	0.0	0.0	0.47	0.42	0.44	IN	212.67	38.67
211	Game software services	1	0.0	0.0	0.59	0.44	0.45	IN	90.18	17.34
212	Electrical household appliances	1	0.0	0.0	0.43	0.43	0.45	IN	10.99	24.43

S5 TABLE: the firms' information of the electric machinery industry (subcommunity 2-18). "-" in some fields indicates missing information

Firm no.	Sector	Degree	Betweenness	Clustering	R_{lr}	D_{lr}	B_{lr}	Bow-tie component (Kyoto)	Profitability	Productivity
1	Generators, motors and other rotating electrical machinery	128	0.1345	0.0005	0.99	0.96	0.99	SCC	2.34	103.67
2	Mechanical power transmission equipment, except ball and roller bearings	4	0.0001	0.125	0.6	0.65	0.67	SCC	110.56	24.63
3	Industrial process controlling instruments	4	0.0	0.125	0.63	0.68	0.67	SCC	35.28	45.23
4	Computer, except personal computer	4	0.0	0.125	0.56	0.58	0.66	SCC	38.48	90.21
5	Printer	3	0.0068	0.0833	0.52	0.37	0.59	SCC	4.86	40.38
6	Fabricated plate work and sheet metal work	3	0.0001	0.1667	0.58	0.65	0.64	IN	-	-
7	Generators, motors and other rotating electrical machinery	3	0.0	0.1667	0.59	0.7	0.62	SCC	51.0	16.13
8	Miscellaneous industry machinery and equipment	3	0.0	0.1667	0.61	0.68	0.65	SCC	46.21	66.01
9	Electrical machinery, equipment and supplies, except electrical household appliances	2	0.0067	0.25	0.5	0.64	0.56	SCC	121.69	128.01

10	Fabricated metal products, n.e.c.	2	0.0	0.25	0.57	0.66	0.57	IN	22.21	22.21
11	Metal cutting machine tools	2	0.0	0.25	0.53	0.64	0.61	IN	124.78	51.99
12	Stamped and pressed metal products, except aluminum and aluminum alloys	2	0.0	0.25	0.55	0.68	0.59	IN	50.0	20.83
13	Molds and dies, parts and accessories for metal products	2	0.0	0.25	0.57	0.69	0.58	IN	43.32	14.44
14	Stamped and pressed metal products, except aluminum and aluminum alloys	2	0.0	0.25	0.52	0.61	0.61	IN	39.84	44.26
15	Miscellaneous electronic equipment	2	0.0011	0.25	0.52	0.64	0.58	SCC	19.0	14.62
16	Non-life insurance agents and brokers	2	0.0012	0.0	0.49	0.31	0.5	SCC	31.61	31.27
17	Production machinery and machine parts, n.e.c	2	0.0011	0.25	0.52	0.66	0.57	IN	3.81	44.99
18	Transportation equipment, except motor vehicles	2	0.0068	0.0	0.48	0.29	0.47	OUT	26.45	53.98
19	Mechanical power transmission equipment, except ball and roller bearings	2	0.0	0.25	0.56	0.66	0.61	IN	43.1	17.24
20	Fabricated metal products, n.e.c.	2	0.0	0.25	0.57	0.66	0.57	IN	-	-
21	Electrical machinery, equipment and supplies, except electrical household appliances	2	0.0067	0.25	0.51	0.66	0.57	OUT	64.93	64.93
22	Stamped and pressed metal products, except aluminum and aluminum alloys	2	0.0	0.25	0.5	0.59	0.61	OUT	153.92	13.87
23	Miscellaneous wholesale trade, n.e.c.	2	0.0	0.25	0.55	0.64	0.59	SCC	115.0	88.46
24	Electrical machinery, equipment and supplies, except electrical household appliances	2	0.0	0.25	0.56	0.66	0.6	IN	29.0	48.33
25	Generators, motors and other rotating electrical machinery	1	0.0	0.0	0.44	0.49	0.48	IN	55.51	18.75
26	Metal cutting machine tools	1	0.0	0.0	0.48	0.48	0.49	IN	66.19	36.77
27	Secondary smelting and refining of lead, including lead alloys	1	0.0	0.0	0.46	0.49	0.5	IN	128.9	49.58
28	Coating metal products	1	0.0	0.0	0.5	0.49	0.46	IN	5.29	10.35
29	Plastic profile extrusions	1	0.0	0.0	0.52	0.48	0.48	IN	176.95	73.73
30	Motor vehicles parts and accessories	1	0.0	0.0	0.45	0.5	0.48	IN	82.4	12.12

31	Electrical machinery, equipment and supplies, except electrical household appliances	1	0.0	0.0	0.45	0.47	0.47	IN	12.35	42.73
32	Robots	1	0.0	0.0	0.44	0.49	0.5	IN	-	-
33	Custom software services	1	0.0	0.0	0.45	0.48	0.46	IN	12.53	9.14
34	Machinery for fabrication of plastic and its equipment	1	0.0	0.0	0.53	0.48	0.5	IN	42.21	35.17
35	Electrical machinery, equipment and supplies, except electrical household appliances	1	0.0	0.0	0.52	0.48	0.5	IN	-	-
36	Electrical welding equipment	1	0.0	0.0	0.54	0.49	0.5	IN	26.0	18.57
37	Motor vehicles parts and accessories	1	0.0	0.0	0.44	0.49	0.48	IN	45.25	16.45
38	Paints	1	0.0	0.0	0.5	0.48	0.48	IN	5.77	11.8
39	Robots	1	0.0	0.0	0.6	0.48	0.5	IN	-	-
40	Package software services	1	0.0	0.0	0.51	0.49	0.49	IN	51.86	51.86
41	Steel castings	1	0.0	0.0	0.59	0.46	0.49	IN	32.11	21.41
42	Metal cutting machine tools	1	0.0	0.0	0.52	0.48	0.49	IN	-	-
43	Stamped and pressed aluminum products and aluminum alloys	1	0.0	0.0	0.48	0.48	0.5	IN	11.86	27.49
44	Stamped and pressed metal products, except aluminum and aluminum alloys	1	0.0	0.0	0.5	0.49	0.49	IN	23.44	15.62
45	Generators, motors and other rotating electrical machinery	1	0.0	0.0	0.5	0.48	0.5	IN	37.98	13.45
46	Optical discs and magnetic tapes and discs	1	0.0	0.0	0.55	0.48	0.5	IN	-	-
47	Motor vehicle parts and accessories, except used parts and accessories	1	0.0	0.0	0.46	0.48	0.48	IN	57.84	91.33
48	Miscellaneous industrial electrical apparatus, including those for vehicles and vessels	1	0.0	0.0	0.58	0.48	0.49	IN	11.78	14.72
49	Travel agency, except travel sub-agency	1	0.0	0.0	0.45	0.49	0.5	IN	321.1	71.36
50	Industrial process controlling instruments	1	0.0	0.0	0.53	0.47	0.49	IN	53.55	65.03
51	Metal cutting machine tools	1	0.0	0.0	0.46	0.49	0.49	IN	11.96	10.17
52	Machinists' tools, except powder metallurgy products	1	0.0	0.0	0.51	0.47	0.48	IN	34.35	26.43
53	Miscellaneous retail trade, n.e.c.	1	0.0	0.0	0.45	0.47	0.48	IN	-	-
54	Stamped and pressed aluminum products and aluminum alloys	1	0.0	0.0	0.49	0.49	0.47	IN	-	-

55	Worker dispatching services	1	0.0	0.0	0.47	0.47	0.48	IN	19.25	32.08
56	Business consultants	1	0.0	0.0	0.45	0.47	0.48	IN	34.54	16.39
57	Miscellaneous industrial electrical apparatus, including those for vehicles and vessels	1	0.0	0.0	0.45	0.49	0.49	IN	-	-
58	Lawyers' offices	1	0.0	0.0	0.45	0.49	0.47	IN	-	-
59	Reinforced plastic containers, bathtubs, etc.	1	0.0	0.0	0.45	0.48	0.47	IN	47.09	44.58
60	Non-ferrous die castings, except aluminum and aluminum alloy die castings	1	0.0	0.0	0.56	0.47	0.49	IN	163.52	14.78
61	Optical lenses and prisms	1	0.0	0.0	0.48	0.47	0.49	IN	80.0	24.0
62	Machinists' tools, except powder metallurgy products	1	0.0	0.0	0.46	0.47	0.48	IN	170.0	14.57
63	Non-ferrous die castings, except aluminum and aluminum alloy die castings	1	0.0	0.0	0.52	0.47	0.49	IN	9.33	7.47
64	Coating metal products	1	0.0	0.0	0.48	0.47	0.49	IN	21.67	8.12
65	Stamped and pressed aluminum products and aluminum alloys	1	0.0	0.0	0.5	0.47	0.47	IN	52.34	15.38
66	Electric measuring instruments, except otherwise classified	1	0.0	0.0	0.48	0.49	0.48	IN	3.02	15.12
67	Package software services	1	0.0	0.0	0.45	0.47	0.49	IN	2.3	9.44
68	Miscellaneous wholesale trade, general merchandise	1	0.0	0.0	0.45	0.46	0.49	IN	8.09	32.92
69	Paper	1	0.0	0.0	0.58	0.49	0.49	IN	41.7	29.79
70	Mechanical design services	1	0.0	0.0	0.55	0.48	0.48	IN	-	-
71	Plastic profile extrusions	1	0.0	0.0	0.56	0.47	0.49	IN	35.67	23.78
72	Miscellaneous business services, n.e.c.	1	0.0	0.0	0.41	0.25	0.32	IN	64.8	6.5
73	Custom software services	1	0.0	0.0	0.52	0.49	0.49	IN	3.45	11.5
74	Switchboards and electrical control equipment	1	0.0	0.0	0.44	0.5	0.47	IN	26.33	19.8
75	Miscellaneous household electric appliances	1	0.0	0.0	0.49	0.48	0.49	IN	31.61	15.81
76	Wiring devices and supplies	1	0.0	0.0	0.48	0.48	0.48	IN	-	-
77	Professional services, n.e.c.	1	0.0	0.0	0.45	0.51	0.49	IN	14.95	20.21
78	Architectural design services	1	0.0	0.0	0.55	0.47	0.48	IN	6.04	10.57
79	Molds and dies, parts and accessories for metal products	1	0.0	0.0	0.58	0.49	0.49	IN	30.5	21.79

80	Stamped and pressed aluminum products and aluminum alloys	1	0.0	0.0	0.54	0.47	0.48	IN	-	9.83
81	Professional services, n.e.c.	1	0.0	0.0	0.46	0.47	0.49	IN	-	-
82	Metal cutting machine tools	1	0.0	0.0	0.58	0.48	0.51	IN	18.33	9.17
83	Miscellaneous electronic parts, devices and electronic circuits	1	0.0	0.0	0.54	0.5	0.48	IN	47.27	33.61
84	Miscellaneous industry machinery and equipment	1	0.0	0.0	0.53	0.47	0.49	IN	63.8	13.67
85	Precision measuring machines and instruments	1	0.0	0.0	0.46	0.47	0.49	IN	4.67	4.67
86	Metalworking machinery and equipment	1	0.0	0.0	0.51	0.49	0.48	IN	6.45	12.9
87	Electrical machinery, equipment and supplies, except electrical household appliances	1	0.0	0.0	0.45	0.48	0.48	IN	13.91	269.59
88	Tobacco and smoking article specialty stores	1	0.0	0.0	0.4	0.29	0.38	OUT	24.92	24.92
89	Measuring instruments, physical and chemical instruments and optical instruments	1	0.0	0.0	0.55	0.47	0.49	SCC	126.83	91.78
90	Motorcycle stores	1	0.0	0.0	0.4	0.23	0.31	OUT	-	4.0
91	Miscellaneous industry machinery and equipment	1	0.0	0.0	0.53	0.48	0.47	SCC	38.62	193.11
92	Electrical machinery, equipment and supplies, except electrical household appliances	1	0.0	0.0	0.49	0.48	0.49	OUT	67.18	179.14
93	"Aramono" (kitchenware)	1	0.0	0.0	0.46	0.49	0.48	OUT	-	-
94	Miscellaneous electronic parts, devices and electronic circuits	1	0.0	0.0	0.45	0.49	0.49	OUT	90.29	19.59
95	Electrical machinery, equipment and supplies, except electrical household appliances	1	0.0	0.0	0.44	0.48	0.48	OUT	15.0	-
96	Refrigerating machines and air conditioning apparatus	1	0.0	0.0	0.59	0.48	0.49	OUT	42.1	15.04
97	Electrical machinery, equipment and supplies, except electrical household appliances	1	0.0	0.0	0.44	0.47	0.49	SCC	209.99	101.69
98	External storages	1	0.0	0.0	0.54	0.47	0.48	OUT	217.3	153.16
99	Motor vehicles parts and accessories	1	0.0	0.0	0.54	0.47	0.5	OUT	9.84	71.94

100	Common motor trucking, except special group cargo motor trucking	1	0.0	0.0	0.5	0.48	0.49	SCC	111.82	41.29	
101	Electrical machinery, equipment and supplies, except electrical household appliances	1	0.0	0.0	0.45	0.49	0.48	OUT	76.63	76.63	
102	Electrical machinery, equipment and supplies, except electrical household appliances	1	0.0	0.0	0.45	0.46	0.47	OUT	411.01	345.25	
103	Metalworking machinery and equipment	1	0.0	0.0	0.47	0.47	0.49	IN	105.55	81.19	
104	Eating places, except specialty restaurants	1	0.0	0.0	0.46	0.48	0.46	IN	-	-	
105	Miscellaneous communication equipment and related products	1	0.0	0.0	0.47	0.5	0.49	IN	6.8	10.61	
106	Mechanical power transmission equipment, except ball and roller bearings	1	0.0	0.0	0.44	0.47	0.5	IN	27.5	22.0	
107	Mechanical power transmission equipment, except ball and roller bearings	1	0.0	0.0	0.6	0.49	0.49	IN	16.27	13.2	
108	Plastic profile extrusions	1	0.0	0.0	0.48	0.49	0.47	IN	-	-	
109	Stamped and pressed metal products, except aluminum and aluminum alloys	1	0.0	0.0	0.46	0.47	0.48	IN	73.87	13.43	
110	Bolts, nuts, rivets, machine screws and wood screws	1	0.0	0.0	0.45	0.47	0.48	IN	12.7	10.92	
111	Mechanical power transmission equipment, except ball and roller bearings	1	0.0	0.0	0.59	0.49	0.49	IN	33.15	11.05	
112	Miscellaneous wholesale trade, general merchandise	1	0.0	0.0	0.46	0.48	0.49	IN	7.0	17.5	
113	Motor vehicles parts and accessories	1	0.0	0.0	0.48	0.48	0.48	IN	-	-	
114	General electric work	1	0.0	0.0	0.47	0.49	0.5	IN	32.6	24.45	
115	Men ' s clothing (occidental style)	1	0.0	0.0	0.5	0.47	0.48	IN	78.57	28.45	
116	Non-ferrous metal forgings	1	0.0	0.0	0.43	0.48	0.48	IN	85.01	24.65	
117	Metal cutting machine tools	1	0.0	0.0	0.45	0.46	0.48	IN	2.26	36.23	
118	Plastic profile extrusions	1	0.0	0.0	0.44	0.49	0.5	IN	26.44	30.96	
119	Robots	1	0.0	0.0	0.44	0.47	0.5	IN	104.27	54.88	
120	Iron castings, except cast iron pipes and malleable iron castings	1	0.0	0.0	0.58	0.48	0.49	IN	46.44	24.12	

121	Custom software services	1	0.0	0.0	0.5	0.49	0.5	IN	10.13	11.38
122	Electrical machinery, equipment and supplies, except electrical household appliances	1	0.0	0.0	0.47	0.48	0.47	IN	93.85	56.45
123	Telecommunication work, except Cable TV broadcasting equipment installation work	1	0.0	0.0	0.56	0.48	0.47	IN	-	11.0
124	Stamped and pressed metal products, except aluminum and aluminum alloys	1	0.0	0.0	0.48	0.47	0.49	IN	13.5	10.38
125	Abrasive cloth and paper	1	0.0	0.0	0.55	0.48	0.5	IN	-	-
126	Fabrication of finished plastic products, n.e.c.	1	0.0	0.0	0.48	0.48	0.49	IN	25.03	20.86
127	Robots	1	0.0	0.0	0.43	0.45	0.46	IN	-	-
128	Stamped and pressed aluminum products and aluminum alloys	1	0.0	0.0	0.45	0.47	0.49	IN	25.0	9.26
129	General civil engineering work and building work	1	0.0	0.0	0.47	0.48	0.49	IN	28.74	63.87
130	Miscellaneous industry machinery and equipment	1	0.0	0.0	0.46	0.49	0.49	IN	46.69	163.42
131	Miscellaneous treatment of metal surface	1	0.0	0.0	0.48	0.5	0.5	IN	-	-
132	Miscellaneous industry machinery and equipment	1	0.0	0.0	0.51	0.47	0.49	IN	26.42	58.13

S6 TABLE: the firms' information of the civil engineering industry (subcommunity 1-115). "-" in some fields indicates missing information

Firm no.	Sector	Degree	Betweenness	Clustering	R_{lr}	D_{lr}	B_{lr}	Bow-tie component (Kyoto)	Profitability	Productivity
1	Miscellaneous building materials	63	0.3178	0.0017	0.97	0.9	0.98	SCC	469.01	226.13
2	Building work, except wooden building work	6	0.0007	0.125	0.64	0.46	0.73	SCC	36.15	36.32
3	General civil engineering work and building work	5	0.0079	0.25	0.57	0.46	0.67	SCC	13.85	52.89
4	Wooden building work	3	0.0088	0.0	0.49	0.38	0.5	SCC	-	23.17
5	Steel-frame work	3	0.0088	0.0	0.48	0.35	0.5	SCC	8.17	16.34
6	Building work, except wooden building work	3	0.0	0.5	0.56	0.46	0.6	SCC	36.79	173.13
7	Steel-frame work	3	0.0	0.25	0.51	0.65	0.6	SCC	176.91	70.76
8	Wooden building work	2	0.0	0.0	0.47	0.32	0.52	OUT	-	-
9	Building work, except wooden building work	2	0.0	0.25	0.6	0.46	0.63	OUT	2.58	9.29

10	Scaffolding work	2	0.0	0.5	0.51	0.65	0.61	IN	139.47	139.47
11	Steel-frame work	2	0.0	0.25	0.53	0.46	0.63	IN	-	-
12	Building work, except wooden building work	2	0.0	0.25	0.57	0.47	0.63	OUT	38.98	124.74
13	Steel-frame work	2	0.0	0.0	0.48	0.5	0.49	SCC	97.8	39.12
14	Building work, except wooden building work	2	0.0	0.0	0.55	0.48	0.49	SCC	25.17	31.46
15	Building work, except wooden building work	2	0.0	0.0	0.6	0.49	0.5	SCC	48.15	22.76
16	Steel-frame work	2	0.0	0.0	0.48	0.41	0.53	IN	-	30.5
17	Steel-frame work	2	0.0	0.0	0.48	0.49	0.5	SCC	68.11	54.49
18	Steel-frame work	2	0.0111	0.0	0.44	0.3	0.51	SCC	27.08	6.91
19	Wooden building work	1	0.0	0.0	0.58	0.47	0.5	OUT	29.08	26.44
20	Sheet-metal work	1	0.0	0.0	0.55	0.49	0.5	IN	20.33	18.48
21	Steel-frame work	1	0.0	0.0	0.58	0.49	0.48	IN	19.95	33.26
22	Metal furnishing work	1	0.0	0.0	0.48	0.47	0.5	IN	19.42	32.36
23	Water-proofing work	1	0.0	0.0	0.53	0.49	0.49	IN	50.8	111.77
24	Steel-frame work	1	0.0	0.0	0.53	0.5	0.49	IN	20.81	20.81
25	Iron framework	1	0.0	0.0	0.46	0.5	0.5	IN	270.0	23.14
26	Miscellaneous household electric appliances	1	0.0	0.0	0.46	0.48	0.5	IN	24.56	61.4
27	Construction work by specialist contractor, n.e.c.	1	0.0	0.0	0.51	0.5	0.5	IN	4.43	22.14
28	Steel-frame work	1	0.0	0.0	0.47	0.49	0.51	IN	3.19	12.58
29	Iron framework	1	0.0	0.0	0.47	0.48	0.49	IN	35.22	39.13
30	Electrical machinery, equipment and supplies, except electrical household appliances	1	0.0	0.0	0.47	0.47	0.5	IN	34.69	115.63
31	Building work, except wooden building work	1	0.0	0.0	0.56	0.5	0.51	IN	-	-
32	Steel-frame work	1	0.0	0.0	0.61	0.5	0.49	IN	60.69	31.09
33	Roofing work, except metal roofing work	1	0.0	0.0	0.34	0.31	0.35	IN	-	10.67
34	Scaffolding work	1	0.0	0.0	0.58	0.48	0.5	IN	-	47.93
35	Building work, except wooden building work	1	0.0	0.0	0.49	0.5	0.5	OUT	43.78	57.61
36	Building work, except wooden building work	1	0.0	0.0	0.51	0.48	0.49	OUT	105.94	102.52
37	Steel-frame work	1	0.0	0.0	0.5	0.48	0.5	IN	164.0	71.31
38	Iron framework	1	0.0	0.0	0.52	0.48	0.5	IN	37.3	26.64
39	Scaffolding work	1	0.0	0.0	0.4	0.28	0.34	IN	9.46	9.46
40	Steel-frame work	1	0.0	0.0	0.46	0.48	0.5	IN	72.29	65.72
41	Miscellaneous building materials	1	0.0	0.0	0.56	0.5	0.49	OUT	-	-
42	Steel-frame work	1	0.0	0.0	0.48	0.48	0.5	IN	3.73	9.31
43	Miscellaneous wholesale trade, general merchandise	1	0.0	0.0	0.63	0.49	0.49	OUT	3.02	10.08
44	Steel-frame work	1	0.0	0.0	0.54	0.51	0.49	OUT	9.37	16.87

45	Miscellaneous secondary smelting and refining of non-ferrous metals, including non-ferrous alloys	1	0.0	0.0	0.46	0.49	0.5	OUT	31.6	48.61
46	Secondary smelting and refining of lead, including lead alloys	1	0.0	0.0	0.46	0.49	0.51	SCC	-	-
47	Secondary smelting and refining of lead, including lead alloys	1	0.0	0.0	0.45	0.48	0.49	OUT	116.75	36.46
48	Building work, except wooden building work	1	0.0	0.0	0.49	0.49	0.49	OUT	68.62	162.52
49	Talc mining	1	0.0	0.0	0.56	0.49	0.49	OUT	6.56	6.56
50	Steel-frame work	1	0.0	0.0	0.52	0.5	0.5	OUT	1.01	3.36
51	Miscellaneous food and beverages	1	0.0	0.0	0.46	0.49	0.5	OUT	12.7	33.57
52	Building work, except wooden building work	1	0.0	0.0	0.51	0.51	0.49	OUT	109.92	113.47
53	Pallets	1	0.0	0.0	0.3	0.25	0.34	OUT	10.75	6.32
54	Steel reinforcement work	1	0.0	0.0	0.5	0.48	0.49	OUT	6.47	12.94
55	Miscellaneous building materials	1	0.0	0.0	0.34	0.27	0.35	IN	38.36	47.73
56	Steel-frame work	1	0.0	0.0	0.47	0.49	0.49	IN	66.73	60.67
57	Steel-frame work	1	0.0	0.0	0.48	0.49	0.5	IN	32.39	53.99
58	Architectural design services	1	0.0	0.0	0.45	0.48	0.49	IN	-	-
59	Sheet-metal work	1	0.0	0.0	0.5	0.49	0.49	IN	27.55	68.87
60	Civil engineering work, except otherwise classified	1	0.0	0.0	0.34	0.36	0.34	TUBE	7.52	22.56

S7 TABLE: Regression of R_{lr} for the Nishijin silk fabric industry
Multiple R-squared: 0.7784, Adjusted R-squared: 0.7661
F-statistic: 63.24 on 7 and 126 DF, p-value: $< 2.2 \times 10^{-16}$

Variable	Estimate	Std. Error	t value	$Pr(>\|t\|)$ 0 ‘ *** ’ 0.001 ‘ ** ’ 0.01 ‘ * ’ 0.05 ‘ . ’ 0.1 ‘ ’ 1
(Intercept)	3.873×10^{-1}	1.672×10^{-2}	23.157	$< 2 \times 10^{-16}$ ***
Degree	2.405×10^{-2}	2.070×10^{-3}	11.618	$< 2 \times 10^{-16}$ ***
Betweenness	$-2.573\times10^{+1}$	$3.551\times10^{+0}$	-7.245	3.82×10^{-11} ***
Clustering	8.031×10^{-2}	1.847×10^{-1}	0.435	0.665
OUT	-6.389×10^{-3}	1.914×10^{-2}	-0.334	0.739
SCC	8.940×10^{-2}	1.788×10^{-2}	5.000	1.88×10^{-6} ***
Profitability	-3.221×10^{-4}	3.451×10^{-4}	-0.933	0.352
Productivity	-9.097×10^{-5}	1.077×10^{-4}	-0.845	0.400

S8 TABLE: Regression of D_{lr} for the Nishijin silk fabric industry
Multiple R-squared: 0.2646, Adjusted R-squared: 0.2237
F-statistic: 6.476 on 7 and 126 DF, p-value: 1.591×10^{-6}

Variable	Estimate	Std. Error	t value	$Pr(>\|t\|)$ 0 ‘ *** ’ 0.001 ‘ ** ’ 0.01 ‘ * ’ 0.05 ‘ . ’ 0.1 ‘ ’ 1
(Intercept)	0.3254863	0.0309020	10.533	$<2\times10^{-16}$ ***
Degree	0.0093391	0.0038258	2.441	0.0160 *
Betweenness	-1.7137814	6.5619534	-0.261	0.7944

Clustering	0.7499660	0.3413918	2.197	0.0299 *
OUT	-0.0408236	0.0353599	-1.155	0.2505
SCC	-0.0402361	0.0330421	-1.218	0.2256
Profitability	-0.0002884	0.0006377	-0.452	0.6519
Productivity	0.0004442	0.0001990	2.232	0.0274 *

S9 TABLE: Regression of B_{lr} for the Nishijin silk fabric industry
Multiple R-squared: 0.7617, Adjusted R-squared: 0.7484
F-statistic: 57.53 on 7 and 126 DF, p-value: $< 2.2{\times}10^{-16}$

Variable	Estimate	Std. Error	t value	$Pr(>\|t\|)$ 0 ‘ *** ’ 0.001 ‘ ** ’ 0.01 ‘ * ’ 0.05 ‘ . ’ 0.1 ‘ ’ 1
(Intercept)	$3.289{\times}10^{-1}$	$2.322{\times}10^{-2}$	14.164	$< 2{\times}10^{-16}$ ***
Degree	$3.161{\times}10^{-2}$	$2.875{\times}10^{-3}$	10.994	$< 2{\times}10^{-16}$ ***
Betweenness	$-3.756{\times}10^{+1}$	4.931	-7.617	$5.39{\times}10^{-12}$ ***
Clustering	$1.264{\times}10^{-1}$	$2.565{\times}10^{-1}$	0.493	0.623
OUT	$8.032{\times}10^{-3}$	$2.657{\times}10^{-2}$	0.302	0.763
SCC	$1.502{\times}10^{-1}$	$2.483{\times}10^{-2}$	6.048	$1.55{\times}10^{-8}$ ***
Profitability	$-4.626{\times}10^{-5}$	$4.792{\times}10^{-4}$	-0.097	0.923
Productivity	$-9.125{\times}10^{-5}$	$1.496{\times}10^{-4}$	-0.610	0.543

S10 TABLE: Regression of the Profitability for the Nishijin silk fabric industry
Multiple R-squared: 0.1089, Adjusted R-squared: 0.05191
F-statistic: 1.91 on 8 and 125 DF, p-value: 0.06402

Variable	Estimate	Std. Error	t value	$Pr(>\|t\|)$ 0 ‘ *** ’ 0.001 ‘ ** ’ 0.01 ‘ * ’ 0.05 ‘ . ’ 0.1 ‘ ’ 1
(Intercept)	25.4095	11.5727	2.196	0.02996 *
Degree	2.0674	0.7815	2.645	0.00921 **
Betweenness	-2589.7753	1128.8850	-2.294	0.02345 *
Clustering	-18.3333	51.1317	-0.359	0.72054
R_{lr}	-108.3822	49.5928	-2.185	0.03072 *
D_{lr}	8.9764	13.0564	0.688	0.49303
B_{lr}	61.2557	35.7431	1.714	0.08905 .
OUT	6.2632	5.2273	1.198	0.23312
SCC	2.3357	5.5922	0.418	0.67691

S11 TABLE: Regression of the Productivity for the Nishijin silk fabric industry
Multiple R-squared: 0.1326, Adjusted R-squared: 0.07706
F-statistic: 2.388 on 8 and 125 DF, p-value: 0.0198

Variable	Estimate	Std. Error	t value	$Pr(>\|t\|)$ 0 ‘ *** ’ 0.001 ‘ ** ’ 0.01 ‘ * ’ 0.05 ‘ . ’ 0.1 ‘ ’ 1
(Intercept)	51.037	36.552	1.396	0.16511
Degree	6.795	2.468	2.753	0.00679 **
Betweenness	-10291.450	3565.539	-2.886	0.00459 **
Clustering	-103.287	161.497	-0.640	0.52363
R_{lr}	-263.346	156.637	-1.681	0.09521 .
D_{lr}	104.714	41.238	2.539	0.01233 *
B_{lr}	110.577	112.893	0.979	0.32923
OUT	10.675	16.510	0.647	0.51910
SCC	1.020	17.663	0.058	0.95405

S12 TABLE: Regression of R_{lr} for the Kyoyuzen dyeing industry
Multiple R-squared: 0.7407, Adjusted R-squared: 0.7168
F-statistic: 31.01 on 7 and 76 DF, p-value: $< 2.2{\times}10^{-16}$

Variable	Estimate	Std. Error	t value	$Pr(>\|t\|)$ 0 '***' 0.001 '**' 0.01 '*' 0.05 '.' 0.1 ' ' 1
(Intercept)	4.338×10^{-1}	1.759×10^{-2}	24.661	$< 2\times10^{-16}$ ***
Degree	2.022×10^{-2}	4.055×10^{-3}	4.986	3.78×10^{-6} ***
Betweenness	-2.824	9.046×10^{-1}	-3.122	0.00254 **
Clustering	1.003×10^{-1}	8.644×10^{-2}	1.161	0.24947
OUT	-1.641×10^{-3}	2.005×10^{-2}	-0.082	0.93499
SCC	6.259×10^{-2}	1.889×10^{-2}	3.313	0.00142 **
Profitability	6.698×10^{-5}	2.939×10^{-4}	0.228	0.82035
Productivity	-1.600×10^{-4}	2.876×10^{-4}	-0.556	0.57971

S13 TABLE: Regression of D_{lr} for the Kyoyuzen dyeing industry
Multiple R-squared: 0.6799, Adjusted R-squared: 0.6505
F-statistic: 23.06 on 7 and 76 DF, p-value: $< 2.2\times10^{-16}$

Variable	Estimate	Std. Error	t value	$Pr(>\|t\|)$ 0 '***' 0.001 '**' 0.01 '*' 0.05 '.' 0.1 ' ' 1
(Intercept)	0.4246674	0.0211910	20.040	$< 2\times10^{-16}$ ***
Degree	0.0281553	0.0048852	5.763	1.67×10^{-7} ***
Betweenness	-4.6213008	1.0896899	-4.241	6.21×10^{-5} ***
Clustering	0.5697714	0.1041223	5.472	5.49×10^{-7} ***
OUT	0.0092761	0.0241463	0.384	0.7019
SCC	0.0225196	0.0227588	0.989	0.3256
Profitability	0.0006647	0.0003540	1.877	0.0643 .
Productivity	-0.0008240	0.0003465	-2.378	0.0199 *

S14 TABLE: Regression of B_{lr} for the Kyoyuzen dyeing industry
Multiple R-squared: 0.6019, Adjusted R-squared: 0.5652
F-statistic: 16.41 on 7 and 76 DF, p-value: 5.562×10^{-13}

Variable	Estimate	Std. Error	t value	$Pr(>\|t\|)$ 0 '***' 0.001 '**' 0.01 '*' 0.05 '.' 0.1 ' ' 1
(Intercept)	0.4012113	0.0277567	14.455	$< 2\times10^{-16}$ ***
Degree	0.0208448	0.0063988	3.258	0.00168 **
Betweenness	-3.2087506	1.4273124	-2.248	0.02747 *
Clustering	0.1381658	0.1363828	1.013	0.31424
OUT	0.0168711	0.0316277	0.533	0.59529
SCC	0.1242260	0.0298102	4.167	8.08×10^{-5} ***
Profitability	-0.0003690	0.0004637	-0.796	0.42866
Productivity	0.0001733	0.0004538	0.382	0.70355

S15 TABLE: Regression of the Profitability for the Kyoyuzen dyeing industry
Multiple R-squared: 0.2361, Adjusted R-squared: 0.1546
F-statistic: 2.898 on 8 and 75 DF, p-value: 0.007195

Variable	Estimate	Std. Error	t value	$Pr(>\|t\|)$ 0 '***' 0.001 '**' 0.01 '*' 0.05 '.' 0.1 ' ' 1
(Intercept)	2.8292	26.1673	0.108	0.914
Degree	0.5077	2.0370	0.249	0.804
Betweenness	171.0691	423.8309	0.404	0.688
Clustering	-56.0693	47.5851	-1.178	0.242
R_{lr}	31.2419	84.3490	0.370	0.712
D_{lr}	48.9140	49.7570	0.983	0.329
B_{lr}	-56.3255	51.5341	-1.093	0.278
OUT	7.3753	9.2471	0.798	0.428
SCC	5.0594	9.7249	0.520	0.604

S16 TABLE: Regression of the Productivity for the Kyoyuzen dyeing industry
Multiple R-squared: 0.4294, Adjusted R-squared: 0.3686
F-statistic: 7.056 on 8 and 75 DF, p-value: 6.561×10^{-7}

Variable	Estimate	Std. Error	t value	$Pr(>\|t\|)$<br>0 ‘ *** ’ 0.001 ‘ ** ’ 0.01 ‘ * ’ 0.05 ‘ . ’ 0.1 ‘ ’ 1
(Intercept)	$4.135\times10^{+1}$	$2.653\times10^{+1}$	1.559	0.123257
Degree	8.198	2.065	3.970	0.000164 ***
Betweenness	$-1.305\times10^{+3}$	$4.297\times10^{+2}$	-3.038	0.003281 **
Clustering	$-1.513\times10^{+1}$	$4.824\times10^{+1}$	-0.314	0.754737
R_{lr}	$-2.028\times10^{+1}$	$8.552\times10^{+1}$	-0.237	0.813223
D_{lr}	$-8.137\times10^{+1}$	$5.045\times10^{+1}$	-1.613	0.110961
B_{lr}	$3.762\times10^{+1}$	$5.225\times10^{+1}$	0.720	0.473746
OUT	7.818	9.375	0.834	0.406972
SCC	-2.633×10^{-2}	9.860	-0.003	0.997876

S17 TABLE: Regression of R_{lr} for the Kyoto doll industry
Multiple R-squared: 0.8273, Adjusted R-squared: 0.8054
F-statistic: 37.65 on 7 and 55 DF, p-value: $< 2.2\times10^{-16}$

Variable	Estimate	Std. Error	t value	$Pr(>\|t\|)$<br>0 ‘ *** ’ 0.001 ‘ ** ’ 0.01 ‘ * ’ 0.05 ‘ . ’ 0.1 ‘ ’ 1
(Intercept)	3.599×10^{-1}	2.541×10^{-2}	14.162	$< 2\times10^{-16}$ ***
Degree	2.717×10^{-2}	2.859×10^{-3}	9.503	3.35×10^{-13} ***
Betweenness	-8.140	1.603	-5.079	4.68×10^{-6} ***
Clustering	2.942×10^{-1}	9.364×10^{-2}	3.142	0.0027 **
OUT	6.188×10^{-2}	2.601×10^{-2}	2.379	0.0208 *
SCC	6.736×10^{-2}	3.427×10^{-2}	1.966	0.0544 .
Profitability	-6.412×10^{-5}	1.308×10^{-4}	-0.490	0.6259
Productivity	1.825×10^{-4}	3.468×10^{-4}	0.526	0.6009

S18 TABLE: Regression of D_{lr} for the Kyoto doll industry
Multiple R-squared: 0.2816, Adjusted R-squared: 0.1901
F-statistic: 3.079 on 7 and 55 DF, p-value: 0.008251

Variable	Estimate	Std. Error	t value	$Pr(>\|t\|)$<br>0 ‘ *** ’ 0.001 ‘ ** ’ 0.01 ‘ * ’ 0.05 ‘ . ’ 0.1 ‘ ’ 1
(Intercept)	0.2647251	0.0785548	3.370	0.00138 **
Degree	0.0012835	0.0088391	0.145	0.88508
Betweenness	5.0751270	4.9546444	1.024	0.31017
Clustering	-0.8661953	0.2894756	-2.992	0.00414 **
OUT	0.0507075	0.0803910	0.631	0.53081
SCC	-0.0867599	0.1059379	-0.819	0.41634
Profitability	0.0001476	0.0004042	0.365	0.71651
Productivity	0.0014969	0.0010720	1.396	0.16820

S19 TABLE: Regression of B_{lr} for the Kyoto doll industry
Multiple R-squared: 0.7572, Adjusted R-squared: 0.7262
F-statistic: 24.5 on 7 and 55 DF, p-value: 8.919×10^{-15}

Variable	Estimate	Std. Error	t value	$Pr(>\|t\|)$<br>0 ‘ *** ’ 0.001 ‘ ** ’ 0.01 ‘ * ’ 0.05 ‘ . ’ 0.1 ‘ ’ 1

(Intercept)	3.117×10^{-1}	4.032×10^{-2}	7.731	2.40×10^{-10} * **
Degree	4.347×10^{-2}	4.537×10^{-3}	9.580	2.54×10^{-13} * **
Betweenness	$-1.841\times10^{+1}$	2.543	-7.239	1.53×10^{-9} * **
Clustering	6.126×10^{-1}	1.486×10^{-1}	4.123	0.000127 ***
OUT	6.449×10^{-2}	4.127×10^{-2}	1.563	0.123849
SCC	1.311×10^{-1}	5.438×10^{-2}	2.411	0.019273 *
Profitability	-1.734×10^{-4}	2.075×10^{-4}	-0.836	0.406904
Productivity	1.298×10^{-4}	5.503×10^{-4}	0.236	0.814373

S20 TABLE: Regression of the Profitability for the Kyoto doll industry
Multiple R-squared: 0.1092, Adjusted R-squared: -0.02282
F-statistic: 0.8271 on 8 and 54 DF, p-value: 0.5826

Variable	Estimate	Std. Error	t value	$Pr(>\|t\|)$<br>0 '***' 0.001 '**' 0.01 '*' 0.05 '.' 0.1 ' ' 1
(Intercept)	1.481	63.225	0.023	0.981
Degree	8.200	5.227	1.569	0.123
Betweenness	-4116.376	2444.521	-1.684	0.098 .
Clustering	62.356	127.636	0.489	0.627
R_{lr}	80.725	243.017	0.332	0.741
D_{lr}	47.936	47.952	1.000	0.322
B_{lr}	-133.884	150.690	-0.888	0.378
OUT	25.718	29.506	0.872	0.387
SCC	29.000	38.848	0.747	0.459

S21 TABLE: Regression of the Productivity for the Kyoto doll industry
Multiple R-squared: 0.117, Adjusted R-squared: -0.01378
F-statistic: 0.8947 on 8 and 54 DF, p-value: 0.5275

Variable	Estimate	Std. Error	t value	$Pr(>\|t\|)$<br>0 '***' 0.001 '**' 0.01 '*' 0.05 '.' 0.1 ' ' 1
(Intercept)	9.279	23.627	0.393	0.696
Degree	1.461	1.953	0.748	0.458
Betweenness	-1177.856	913.513	-1.289	0.203
Clustering	48.844	47.697	1.024	0.310
R_{lr}	40.075	90.815	0.441	0.661
D_{lr}	27.913	17.919	1.558	0.125
B_{lr}	-33.236	56.313	-0.590	0.558
OUT	-1.471	11.026	-0.133	0.894
SCC	23.759	14.517	1.637	0.108

S22 TABLE: Regression of R_{lr} for the consumer games industry
Multiple R-squared: 0.6447, Adjusted R-squared: 0.6285
F-statistic: 39.66 on 7 and 153 DF, p-value: $< 2.2\times10^{-16}$

Variable	Estimate	Std. Error	t value	$Pr(>\|t\|)$<br>0 '***' 0.001 '**' 0.01 '*' 0.05 '.' 0.1 ' ' 1
(Intercept)	4.513×10^{-1}	8.546×10^{-3}	52.811	$< 2\times10^{-16}$ * **
Degree	3.134×10^{-2}	3.360×10^{-3}	9.326	$< 2\times10^{-16}$ * **
Betweenness	$-2.425\times10^{+1}$	2.778	-8.730	4.1e-15 ***
Clustering	4.319×10^{-2}	2.513×10^{-2}	1.719	0.0876 .
IN	-8.095×10^{-3}	8.393×10^{-3}	-0.964	0.3363
SCC	-2.943×10^{-2}	1.710×10^{-2}	-1.721	0.0872 .
Profitability	-1.030×10^{-6}	1.471×10^{-5}	-0.070	0.9443
Productivity	-5.702×10^{-6}	3.486×10^{-5}	-0.164	0.8703

S23 TABLE: Regression of D_{lr} for the consumer games industry
Multiple R-squared: 0.8069, Adjusted R-squared: 0.798
F-statistic: 91.31 on 7 and 153 DF, p-value: $< 2.2\times10^{-16}$

Variable	Estimate	Std. Error	t value	$Pr(>\|t\|)$ 0 '***' 0.001 '**' 0.01 '*' 0.05 '.' 0.1 ' ' 1
(Intercept)	4.116×10^{-1}	8.672×10^{-3}	47.459	$<2\times10^{-16}$ ***
Degree	3.495×10^{-2}	3.410×10^{-3}	10.250	$<2\times10^{-16}$ ***
Betweenness	$-2.720\times10^{+1}$	2.819	-9.650	$<2\times10^{-16}$ ***
Clustering	3.378×10^{-1}	2.550×10^{-2}	13.250	$<2\times10^{-16}$ ***
IN	-9.784×10^{-3}	8.517×10^{-3}	-1.149	0.252
SCC	-2.702×10^{-2}	1.735×10^{-2}	-1.558	0.121
Profitability	-4.009×10^{-6}	1.493×10^{-5}	-0.269	0.789
Productivity	3.383×10^{-6}	3.537×10^{-5}	0.096	0.924

S24 TABLE: Regression of B_{lr} for the consumer games industry
Multiple R-squared: 0.927, Adjusted R-squared: 0.9237
F-statistic: 277.6 on 7 and 153 DF, p-value: $< 2.2\times10^{-16}$

Variable	Estimate	Std. Error	t value	$Pr(>\|t\|)$ 0 '***' 0.001 '**' 0.01 '*' 0.05 '.' 0.1 ' ' 1
(Intercept)	3.974×10^{-1}	5.112×10^{-3}	77.740	$< 2\times10^{-16}$ ***
Degree	5.301×10^{-2}	2.010×10^{-3}	26.374	$< 2\times10^{-16}$ ***
Betweenness	$-4.224\times10^{+1}$	1.661	-25.422	$< 2\times10^{-16}$ ***
Clustering	1.726×10^{-1}	1.503×10^{-2}	11.480	$< 2\times10^{-16}$ ***
IN	-5.186×10^{-3}	5.021×10^{-3}	-1.033	0.303
SCC	-4.460×10^{-2}	1.023×10^{-2}	-4.361	2.37×10^{-5} ***
Profitability	-9.267×10^{-6}	8.800×10^{-6}	-1.053	0.294
Productivity	1.615×10^{-5}	2.085×10^{-5}	0.775	0.440

S25 TABLE: Regression of the Profitability for the consumer games industry
Multiple R-squared: 0.04992, Adjusted R-squared: -8.445×10^{-5}
$F - statistic : 0.9983 on 8 and 152 DF, p - value : 0.4398$

Variable	Estimate	Std. Error	t value	$Pr(>\|t\|)$ 0 '***' 0.001 '**' 0.01 '*' 0.05 '.' 0.1 ' ' 1
(Intercept)	$1.091\times10^{+3}$	$8.458\times10^{+2}$	1.290	0.1991
Degree	$1.182\times10^{+2}$	$1.131\times10^{+2}$	1.045	0.2979
Betweenness	$-9.464\times10^{+4}$	$9.077\times10^{+4}$	-1.043	0.2988
Clustering	5.475×10^{-1}	$5.424\times10^{+2}$	0.001	0.9992
R_{lr}	$-4.422\times10^{+2}$	$1.179\times10^{+3}$	-0.375	0.7081
D_{lr}	$-3.963\times10^{+1}$	$1.332\times10^{+3}$	-0.030	0.9763
B_{lr}	$-1.448\times10^{+3}$	$2.310\times10^{+3}$	-0.627	0.5316
IN	$-2.847\times10^{+2}$	$1.157\times10^{+2}$	-2.461	0.0150 *
SCC	$-4.375\times10^{+2}$	$2.503\times10^{+2}$	-1.748	0.0825 .

S26 TABLE: Regression of the Productivity for the consumer games industry
Multiple R-squared: 0.09426, Adjusted R-squared: 0.04659
F-statistic: 1.977 on 8 and 152 DF, p-value: 0.05283

Variable	Estimate	Std. Error	t value	$Pr(>\|t\|)$ 0 '***' 0.001 '**' 0.01 '*' 0.05 '.' 0.1 ' ' 1
(Intercept)	372.20	357.36	1.042	0.29929
Degree	63.74	47.80	1.334	0.18435
Betweenness	-51281.47	38351.43	-1.337	0.18317
Clustering	-52.47	229.18	-0.229	0.81921

R_{lr}	-245.34	498.10	-0.493	0.62304
D_{lr}	-101.20	562.65	-0.180	0.85750
B_{lr}	-217.16	975.98	-0.223	0.82422
IN	-151.79	48.88	-3.105	0.00227 **
SCC	-270.10	105.76	-2.554	0.01164 *

S27 TABLE: Regression of R_{lr} for the electric machinery industry
Multiple R-squared: 0.6045, Adjusted R-squared: 0.5765
F-statistic: 21.61 on 7 and 99 DF, p-value: $< 2.2\times10^{-16}$

Variable	Estimate	Std. Error	t value	$Pr(>\|t\|)$ 0 ‘ *** ’ 0.001 ‘ ** ’ 0.01 ‘ * ’ 0.05 ‘ . ’ 0.1 ‘ ’ 1
(Intercept)	4.941×10^{-1}	1.707×10^{-2}	28.943	$< 2\times10^{-16}$ ***
Degree	1.282×10^{-2}	3.887×10^{-3}	3.299	0.00135 **
Betweenness	-8.604	3.658	-2.352	0.02065 *
Clustering	1.653×10^{-1}	5.506×10^{-2}	3.003	0.00338 **
IN	-9.824×10^{-3}	1.636×10^{-2}	-0.601	0.54952
SCC	2.115×10^{-2}	1.927×10^{-2}	1.098	0.27487
Profitability	-8.920×10^{-5}	8.290×10^{-5}	-1.076	0.28451
Productivity	-7.448×10^{-5}	1.041×10^{-4}	-0.715	0.47606

S28 TABLE: Regression of D_{lr} for the electric machinery industry
Multiple R-squared: 0.8052, Adjusted R-squared: 0.7914
F-statistic: 58.45 on 7 and 99 DF, p-value: $< 2.2\times10^{-16}$

Variable	Estimate	Std. Error	t value	$Pr(>\|t\|)$ 0 ‘ *** ’ 0.001 ‘ ** ’ 0.01 ‘ * ’ 0.05 ‘ . ’ 0.1 ‘ ’ 1
(Intercept)	4.186×10^{-1}	1.577×10^{-2}	26.539	$< 2\times10^{-16}$ ***
Degree	2.048×10^{-2}	3.591×10^{-3}	5.703	1.23×10^{-7} ***
Betweenness	$-1.568\times10^{+1}$	3.379	-4.641	1.06×10^{-5} ***
Clustering	7.421×10^{-1}	5.086×10^{-2}	14.591	$< 2\times10^{-16}$ ***
IN	3.178×10^{-2}	1.511×10^{-2}	2.103	0.038 *
SCC	1.757×10^{-2}	1.780×10^{-2}	0.987	0.326
Profitability	5.639×10^{-6}	7.658×10^{-5}	0.074	0.941
Productivity	1.111×10^{-4}	9.618×10^{-5}	1.155	0.251

S29 TABLE: Regression of B_{lr} for the electric machinery industry
Multiple R-squared: 0.8594, Adjusted R-squared: 0.8495
F-statistic: 86.44 on 7 and 99 DF, p-value: $< 2.2\times10^{-16}$

Variable	Estimate	Std. Error	t value	$Pr(>\|t\|)$ 0 ‘ *** ’ 0.001 ‘ ** ’ 0.01 ‘ * ’ 0.05 ‘ . ’ 0.1 ‘ ’ 1
(Intercept)	4.731×10^{-1}	1.040×10^{-2}	45.490	$< 2\times10^{-16}$ ***
Degree	1.279×10^{-2}	2.368×10^{-3}	5.403	4.52e-07 ***
Betweenness	-8.558	2.228	-3.841	0.000216 ***
Clustering	4.173×10^{-1}	3.354×10^{-2}	12.442	$< 2\times10^{-16}$ ***
IN	-5.172×10^{-4}	9.964×10^{-3}	-0.052	0.958709
SCC	3.680×10^{-2}	1.174×10^{-2}	3.136	0.002256 **
Profitability	-2.399×10^{-5}	5.050×10^{-5}	-0.475	0.635718
Productivity	-1.723×10^{-5}	6.342×10^{-5}	-0.272	0.786404

S30 TABLE: Regression of the Profitability for the electric machinery industry
Multiple R-squared: 0.1585, Adjusted R-squared: 0.08981
F-statistic: 2.307 on 8 and 98 DF, p-value: 0.02604

Variable	Estimate	Std. Error	t value	$Pr(>\|t\|)$<br>0 '***' 0.001 '**' 0.01 '*' 0.05 '.' 0.1 ' ' 1
(Intercept)	207.293	109.011	1.902	0.060165 .
Degree	4.760	6.164	0.772	0.441885
Betweenness	-4449.388	5427.321	-0.820	0.414312
Clustering	-67.361	137.746	-0.489	0.625915
R_{lr}	-219.851	141.896	-1.549	0.124514
D_{lr}	218.405	166.602	1.311	0.192941
B_{lr}	-186.096	261.989	-0.710	0.479192
IN	-73.045	20.479	-3.567	0.000561 ***
SCC	-25.700	26.903	-0.955	0.341774

S31 TABLE: Regression of the Productivity for the electric machinery industry
Multiple R-squared: 0.2303, Adjusted R-squared: 0.1675
F-statistic: 3.666 on 8 and 98 DF, p-value: 0.0008908

Variable	Estimate	Std. Error	t value	$Pr(>\|t\|)$<br>0 '***' 0.001 '**' 0.01 '*' 0.05 '.' 0.1 ' ' 1
(Intercept)	153.087	86.079	1.778	0.0784 .
Degree	-1.308	4.868	-0.269	0.7887
Betweenness	1743.356	4285.571	0.407	0.6850
Clustering	-135.306	108.768	-1.244	0.2165
R_{lr}	-161.913	112.045	-1.445	0.1516
D_{lr}	264.689	131.554	2.012	0.0470 *
B_{lr}	-202.060	206.874	-0.977	0.3311
IN	-67.491	16.171	-4.174	6.5×10^{-5} ***
SCC	-11.131	21.243	-0.524	0.6015

S32 TABLE: regression of R_{lr} for the civil engineering industry
Multiple R-squared: 0.5871, Adjusted R-squared: 0.5183
F-statistic: 8.532 on 7 and 42 DF, p-value: 1.817×10^{-6}

Variable	Estimate	Std. Error	t value	$Pr(>\|t\|)$<br>0 '***' 0.001 '**' 0.01 '*' 0.05 '.' 0.1 ' ' 1
(Intercept)	4.634×10^{-1}	2.549×10^{-2}	18.182	$<2\times10^{-16}$ ***
Degree	3.634×10^{-2}	1.550×10^{-2}	2.344	0.0239 *
Betweenness	-5.372	2.989	-1.797	0.0795 .
Clustering	3.192×10^{-2}	1.125×10^{-1}	0.284	0.7781
IN	-5.843×10^{-3}	2.159×10^{-2}	-0.271	0.7880
SCC	-2.314×10^{-2}	3.233×10^{-2}	-0.716	0.4781
Profitability	-1.115×10^{-4}	1.925×10^{-4}	-0.579	0.5656
Productivity	-8.861×10^{-6}	2.806×10^{-4}	-0.032	0.9750

S33 TABLE: regression of D_{lr} for the civil engineering industry
Multiple R-squared: 0.5565, Adjusted R-squared: 0.4826
F-statistic: 7.529 on 7 and 42 DF, p-value: 7.212×10^{-6}

Variable	Estimate	Std. Error	t value	$Pr(>\|t\|)$<br>0 '***' 0.001 '**' 0.01 '*' 0.05 '.' 0.1 ' ' 1
(Intercept)	0.4157265	0.0263774	15.761	$<2\times10^{-16}$ ***
Degree	0.0205427	0.0160438	1.280	0.2074
Betweenness	-3.2426330	3.0937912	-1.048	0.3006
Clustering	0.0749734	0.1164724	0.644	0.5233
IN	0.0027093	0.0223450	0.121	0.9041
SCC	-0.0462793	0.0334608	-1.383	0.1739
Profitability	0.0004729	0.0001992	2.374	0.0223 *
Productivity	0.0001750	0.0002904	0.603	0.5500

S34 TABLE: Regression of B_{lr} for the civil engineering industry
Multiple R-squared: 0.7834, Adjusted R-squared: 0.7473
F-statistic: 21.71 on 7 and 42 DF, p-value: 4.639×10^{-12}

Variable	Estimate	Std. Error	t value	$Pr(>\|t\|)$ 0 ‘ *** ’ 0.001 ‘ ** ’ 0.01 ‘ * ’ 0.05 ‘ . ’ 0.1 ‘ ’ 1
(Intercept)	4.340×10^{-1}	1.901×10^{-2}	22.831	$< 2\times10^{-16}$ * **
Degree	4.387×10^{-2}	1.156×10^{-2}	3.794	0.000469 ***
Betweenness	-7.017	2.230	-3.147	0.003029 **
Clustering	1.926×10^{-1}	8.394×10^{-2}	2.295	0.026818 *
IN	-4.328×10^{-4}	1.610×10^{-2}	-0.027	0.978686
SCC	-1.268×10^{-2}	2.411×10^{-2}	-0.526	0.601657
Profitability	4.629×10^{-5}	1.435×10^{-4}	0.322	0.748718
Productivity	3.021×10^{-5}	2.093×10^{-4}	0.144	0.885935

S35 TABLE: Regression of the Profitability for the civil engineering industry
Multiple R-squared: 0.693, Adjusted R-squared: 0.6331
F-statistic: 11.57 on 8 and 41 DF, p-value: 2.053×10^{-8}

Variable	Estimate	Std. Error	t value	$Pr(>\|t\|)$ 0 ‘ *** ’ 0.001 ‘ ** ’ 0.01 ‘ * ’ 0.05 ‘ . ’ 0.1 ‘ ’ 1
(Intercept)	-0.4813	69.1796	-0.007	0.994483
Degree	4.6291	13.2628	0.349	0.728854
Betweenness	315.9318	2444.9332	0.129	0.897816
Clustering	19.8840	81.3387	0.244	0.808095
R_{lr}	-351.6565	152.3294	-2.309	0.026088 *
D_{lr}	468.9897	125.7835	3.729	0.000583 ***
B_{lr}	-24.7446	208.8180	-0.118	0.906251
IN	9.3169	15.7436	0.592	0.557243
SCC	20.2672	23.8685	0.849	0.400746

S36 TABLE: Regression of the Productivity for the civil engineering industry
Multiple R-squared: 0.5051, Adjusted R-squared: 0.4085
F-statistic: 5.23 on 8 and 41 DF, p-value: 0.0001536

Variable	Estimate	Std. Error	t value	$Pr(>\|t\|)$ 0 ‘ *** ’ 0.001 ‘ ** ’ 0.01 ‘ * ’ 0.05 ‘ . ’ 0.1 ‘ ’ 1
(Intercept)	27.4134	53.7556	0.510	0.61281
Degree	-1.0647	10.3058	-0.103	0.91822
Betweenness	765.2164	1899.8197	0.403	0.68920
Clustering	184.8086	63.2037	2.924	0.00561 **
R_{lr}	-118.3182	118.3666	-1.000	0.32337
D_{lr}	167.5490	97.7392	1.714	0.09404 .
B_{lr}	-7.7012	162.2607	-0.047	0.96238
IN	0.5076	12.2335	0.041	0.96710
SCC	-7.2750	18.5469	-0.392	0.69690
